# Dual lipid modulation overcomes ferroptosis resistance in high-risk neuroblastoma

**DOI:** 10.1038/s41418-025-01623-3

**Published:** 2025-11-26

**Authors:** Ine Koeken, Magali Walravens, Roberto Fernández-Acosta, Ruben Van Hoyweghen, Iuliana Vintea, Yingyi Kong, Bianka Golba, Jonas Dehairs, Ali Talebi, Johannes V. Swinnen, Kaat Durinck, Adriana Mañas, Shinya Toyokuni, Gerben Menschaert, Maria Fedorova, Bruno G. De Geest, Behrouz Hassannia, Tom Vanden Berghe

**Affiliations:** 1 Cell death Signaling lab, Infla-Med Centre of Excellence, Department of Biomedical Sciences, University of Antwerp, Antwerp, Belgium; 2 Biobix, Lab of Bioinformatics and Computational Genomics, Department of Mathematical Modelling, Statistics and Bioinformatics, Ghent University, Ghent, Belgium; 3 Department of Pathology and Biological Responses, Nagoya University Graduate School of Medicine, Nagoya, Japan; 4 Department of Pharmaceutics, Ghent University, Ghent, Belgium; 5 Laboratory of Lipid Metabolism and Cancer, Department of Oncology, LKI – Leuven Cancer Institute, KU Leuven, Leuven, Belgium; 6 Department of Biomolecular Medicine, Ghent University, Ghent, Belgium; 7 Translational Research in Pediatric Oncology, Hematopoietic Transplantation and Cell Therapy, IdiPAZ Research Center, University Hospital La Paz, Madrid, Spain; 8 Pediatric Onco-hematology Clinical Unit IdiPAZ-CNIO, National Cancer Research Center (CNIO), Madrid, Spain; 9 Center for Low-temperature Plasma Sciences, Nagoya University, Nagoya, Japan; 10 Center for Integrated Sciences of Low-temperature Plasma Core Research (iPlasma Core), Tokai National Higher Education and Research System, Nagoya, Japan; 11 Center of Membrane Biochemistry and Lipid Research, University Hospital and Faculty of Medicine Carl Gustav Carus, Technische Universität Dresden, Dresden, Germany; 12 Department of Biomedical Molecular Biology, Ghent University, Ghent, Belgium

**Keywords:** Paediatric cancer, Cancer metabolism

## Abstract

Ferroptosis—an iron-dependent form of cell death triggered by phospholipid peroxidation—has emerged as a promising therapeutic avenue in cancer treatment. Although neuroblastoma (NB) has been identified as a ferroptosis susceptible cancer, our studies reveal striking heterogeneity in ferroptosis sensitivity across high-risk NB models. Through a targeted metabolic compound screen, we identified stearoyl-CoA desaturase 1 (SCD1)—a key enzyme in monounsaturated fatty acid (MUFA) synthesis—as a robust ferroptosis-sensitizing target. Genetic and pharmacological inhibition of SCD1 restored ferroptosis sensitivity in resistant NB cells. Notably, high SCD1 expression correlates with poor patient prognosis. Co-treatment with arachidonic acid (AA), a polyunsaturated fatty acid (PUFA), further enhanced ferroptotic cell death via increased PUFA/MUFA ratio. Nevertheless, neither baseline lipidomic profiles nor transcriptomes of key ferroptosis regulators reliably predicted ferroptosis sensitivity. To overcome AA’s poor solubility, we engineered AA-loaded lipid nanoparticles (AA-LNPs), which selectively accumulated in high-risk NB tumors and synergized with SCD1 inhibition. This dual-sensitization strategy, termed LipidSens, significantly suppressed tumor growth and induced ferroptosis in cell-derived xenograft mouse models without systemic toxicity. Together, these findings establish MUFA synthesis blockade and PUFA enrichment as a tumor-targeted, ferroptosis-enhancing strategy, and offer a nanomedicine-based therapeutic platform for high-risk NB.

## Highlights

- Functional screen identifies stearoyl-CoA desaturase-1 (SCD1) as a key ferroptosis-sensitizing target.
- Lipidome or transcriptome of major ferroptosis regulators fail to predict ferroptosis sensitivity in NB models.
- Arachidonic acid (AA) supplementation outperforms other strategies to induce ferroptosis sensitivity.
- Lipid nanoparticle (LNP) formulation enables tumor-targeted delivery of AA.
- Dual lipid-modulating strategy combining AA-loaded LNPs (AA-LNPs) and SCD1 inhibition enhances phospholipid unsaturation and sensitizes tumors to ferroptosis.

## Introduction

Ferroptosis has emerged as promising anti-cancer strategy, particularly for eradicating cancer cells that escape conventional therapies [1–3]. This regulated form of cell death is iron-dependent and triggered by excessive peroxidation of poly-unsaturated fatty acids (PUFAs) in cellular membranes [4]. Recent studies, including our own, have demonstrated the therapeutic potential of ferroptosis induction in high-risk neuroblastoma models [5–8].

Neuroblastoma (NB) is an extracranial solid tumor of early childhood, accounting for 8–10% of all pediatric cancers and approximately 15% of pediatric cancer-related mortality [9–11]. Clinically, NB displays remarkable heterogeneity, with outcomes ranging from spontaneous regression to aggressive metastatic disease. Nearly half of the cases are classified as high-risk, characterized by genetic alterations such as MYCN amplifications and poor therapeutic response, often culminating in relapse [12]. Despite multi-modal treatment protocols, the 5 year-event-free survival rate remains around 50% [13], emphasizing the need for novel therapeutic approaches.

Although NB has been classified as a ferroptosis-susceptible cancer type in a large-scale pharmacotranscriptomics screen [14], ferroptosis responsiveness varies significantly across NB models [5]. This heterogeneity in sensitivity poses a critical obstacle to clinical translation of ferroptosis-based therapies. Ferroptosis susceptibility is determined by a complex metabolic landscape that orchestrates both the initiation of lipid peroxidation and the cellular capacity to suppress it [15]. Membrane phospholipid composition is a central determinant of ferroptotic vulnerability [16]. PUFAs are particularly prone to peroxidation due to their multiple double bonds and reactive bis-allylic hydrogen atoms, whereas mono-unsaturated fatty acids (MUFAs) are more resistant to oxidation and act to limit lipid peroxidation [17].

Many cancer cells, including NB, exhibit increased de novo fatty acids (FAs) synthesis to support rapid proliferation and membrane biogenesis [18–20]. However, due to the lack of the Δ12 desaturase enzyme, mammalian cells rely on exogenous PUFA uptake, leading to MUFA- and saturated fatty acid (SFA)-rich membrane profiles [15]. This shift toward lipid saturation contributes to ferroptosis resistance [17]. In parallel, the cellular defense against lipid peroxidation is reinforced by diverse metabolic pathways tied to redox balance, iron handling and amino acid metabolism [21, 22].

In this study, we aimed to uncover metabolic vulnerabilities that could be exploited to overcome ferroptosis resistance in high-risk NB. A focused metabolic compound screen identified the inhibition of stearoyl-CoA 9-desaturase 1 (SCD1), a key enzyme in MUFA-synthesis, as a potent ferroptosis-sensitizing strategy. Building on this, we increased PUFA availability through exogenous supplementation with arachidonic acid (AA), a highly peroxidation-prone PUFA. To enhance the in vivo applicability and tumor selectivity, we formulated AA into lipid nanoparticles (AA-LNPs) for solubilization of AA and tumor-targeted delivery through the enhanced permeation and retention (EPR) effect [23]. This dual lipid-modulating approach—targeting both MUFA suppression and PUFA enrichment—successfully increased membrane unsaturation and restored ferroptosis sensitivity in resistant NB models both in vitro and in vivo. Our findings provide a rational framework for future ferroptosis-based nanotherapeutic strategies in high-risk neuroblastoma.

## Material and methods

Additional methods are provided in the supplementary materials.

### Cell Lines

IMR-32 and SK-N-BE(2 C) cells were cultured in RPMI 1640 medium (21875-034, Gibco) supplemented with 10% fetal bovine serum (FBS, Gibco), 2 mM L-glutamine (25030-081, Gibco), and 100 U/mL Penicillin-Streptomycin (15140-122, Gibco). SH-EP cells were cultured in DMEM medium (41965-039, Gibco) supplemented with 10% FBS and 100 U/mL Penicillin-Streptomycin. SH-SY5Y cells were cultured in DMEM medium, supplemented with 10% FBS, 2mM L-glutamine, 1% of sodium Bicarbonate (25080-094, Gibco), 1 mM sodium pyruvate (S8636, Sigma-Aldrich), 1% of MEM Non-essential Amino Acid Solution (100x, M7145, Sigma-Aldrich). IMR-32 was obtained from Jo Vandesompele, Ghent University Hospital, Medical Research Building, Ghent, Belgium. SK-N-BE (2). C and SH-EP cells were obtained from Paul G. Ekert, Cancer Research, Murdoch Children’s Research Institute, Royal Children’s Hospital. SH-SY5Y cells were purchased (94030304, Sigma). Mycoplasma contamination testing was performed regularly using the Mycoplasma Detection Kit (rep-pt1, InvivoGen).

### In vivo mouse studies

All animal experiments presented were approved by the Ethical Committee of Animal Experimentation of University of Antwerp (Protocol Numbers: 2019-81) and performed according to institutional, national, and European animal regulations. Mice were housed at specific pathogen-free conditions in temperature-controlled (21 °C) animal facilities in 12 h light-dark cycles and provided cage enrichment, water, and fed ad libitum. Female BALB/cAnN-Foxn1nu/Rj or Rj:NMRI-Foxn1nu/nu were purchased from Janvier Labs (France) and acclimatized for 1 week. Cell-derived xenograft (CDX) models were established by injecting cells at a density of 8 × 10^6^, 10 × 10^6^ or 1 × 10^6^ cells in DPBS mixed with Matrigel Matrix (1:1) (356231, Corning) for IMR-32, SH-SY5Y or SK-N-BE (2). C CDX models respectively. A cell suspension of 100 µL was injected subcutaneously. Tumor size was measured by electronic caliper in 3 dimensions (length, width, height) with the formula V = 1/2 × W × H × L, where V is the tumor volume, L is the length, W is the width and H is the height. Tumors for baseline experiments were collected at 100–400mm^3^. For all other following studies, treatment was initiated when tumor volumes reached 100 ± 20 mm³ and intratumoral injection volumes were adjusted based on tumor size: 50 µL for tumors between 100–200 mm³ and 100 µL for tumors exceeding 200 mm³. Note, no injections were given when tumor volumes smaller than 100 mm³ were reached. In RSL3 monotherapy experiments, mice were treated intratumorally every other day with either vehicle (PEG400:Saline solution, 1:1) or RSL3 (1.5 mg/mL in PEG400:Saline solution, 1:1). The ferroptosis induction study with IKE, was performed by daily intraperitoneal injection of vehicle (5% DMSO, 40% PEG300, 5% TWEEN80, 50% ddH2O) or IKE (40 mg/kg in vehicle). We attempted to follow the protocol described by Zhang et al., in which IKE was dissolved in 5% DMSO/95% Hank’s Balanced Salt Solution (HBSS, pH 4) [24]. However, IKE exhibited poor solubility under these conditions and consistently precipitated, even after sonication. We therefore used the alternative formulation recommended by the supplier (SelleckChem), which yielded a stable solution suitable for in vivo administration. The initial combination experiment using AA and MF-438, involved 3 daily intratumoral injections of either vehicle (10% DMSO, 40% PEG300, 2% Tween80, 48% Saline solution) or 5 mM AA and 1 mM MF-438 in vehicle. This was followed by four alternate-day RSL3 injections (as described above). For the LipidSens combination therapy experiments, mice received either vehicle (10% DMSO in DPBS) or LipidSens (Cy7-AA-LNP in DPBS containing 13.2 mM AA and MF-438 was added at 0.5 mM, reaching 10% DMSO). See “LNP formulation” section for more info on the nanoparticles. LipidSens or vehicle injections were alternated daily with intratumoral RSL3 injections (as described above). Treatment continued until tumors reached the humane endpoint of 1500 mm³ or until day 40. To control for systemic toxicity during the treatment period, body weight and physical status of the animals was monitored until they were judged to be in discomfort. When systemic toxicity (weight gain/loss of 20% or more) or maximum tumor volume (1500 mm³) was reached, the animal was euthanized by cervical dislocation. To assess tumor targeting, mice were injected intravenously or intraperitoneally with the Cy7-AA-LNP formulation (200 µL, containing 3.3 mM AA and 6 µM 18:1 Cy7 PE) in DPBS or non-encapsulated 18:1 Cyanine7 PE (6 µM in DPBS containing 5% DMSO). In vivo imaging was performed 72 h post-injection using the Newton FT500 (Vilber, France) or the IVIS Spectrum In Vivo Imaging System (PerkinElmer, USA). Ex vivo images of organs were analyzed by drawing consistent regions of interest (ROIs) around each organ using Quant software for Newton images or Living Image v4.3.1 for IVIS images. Fluorescent signal intensities were normalized to the kidney ROI. For the kinetic imaging experiment, mice received two injections. The first injection was followed by imaging after 72 h, showing minimal signal. Next, a second injection was administered the following day for continued kinetic monitoring.

### Statistics

Sample sizes were determined using G power 3.9.1.7 software and statistical tests were performed using GraphPad Prism 10 (GraphPad Software, USA) or R 4.1.0 in RStudio 2023.12.1.402 (RStudio, USA). Data are presented as mean ± SD or mean ± SEM. Statistical analysis between the two groups was assessed using a two-tailed unpaired t-test. Assumption of variance was validated by an F-test. In cases of more than two groups and/or conditions, one- or two-way ANOVA was used with multiple comparison correction as indicated in figure legends. The compound screen data was analyzed using the Hotelling T2 test (Hotelling v1.3-0).

## Results

### Blocking MUFA-synthesis enzyme SCD1 reverts ferroptosis-resistance

**Fig. 1:**
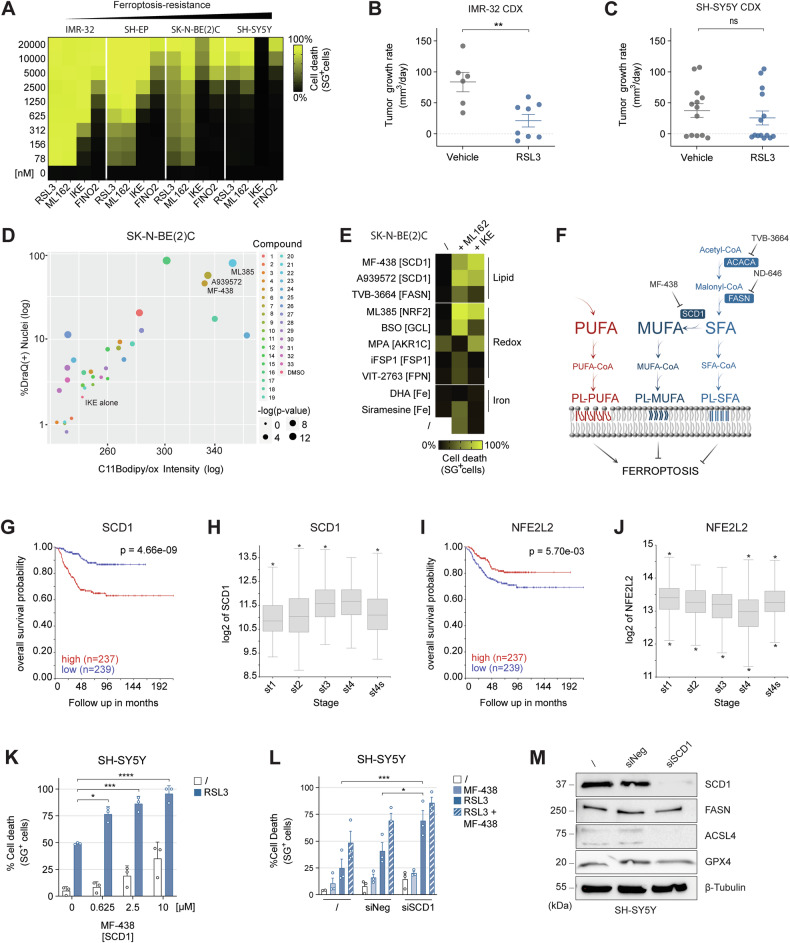
Metabolic compound screen reveals SCD1 as a candidate sensitizing strategy. **A** Heatmap showing mean cell death (%) in high-risk NB cell lines following 24 h treatment with various ferroptosis inducers (*n* = 3; Table S1). **B**, **C** Tumor growth rate (mm³/day) in IMR-32 (vehicle n = 6, RSL3 n = 8) and SH-SY5Y (vehicle n = 12, RSL3 n = 15) cell-derived xenograft (CDX) mice treated with intratumoral RSL3 injections every other day. **D** Percentage of cell death (Draq7-positive cells) and oxidized C11BODIPY intensity in living (Drag7-negative) SK-N-BE (2). C cells treated with 3.5 µM IKE for 11 h following 24 h pretreatment with 1 µM of a custom compound library (Table S2). **E** Heatmap depicting mean cell death (%) of SK-N-BE2C cells pretreated for 24 h with library compounds, followed by 24 h exposure to either ML162 (n = 1) or IKE (n = 2). See also Table S3. **F** Schematic overview of de novo FA synthesis and polyunsaturated fatty acid (PUFA) uptake. **G–J** Kaplan–Meier survival curves and boxplots showing that high SCD1 and low NFE2L2 expression correlate with poorer overall survival and advanced diagnostic stage in the Kocak neuroblastoma patient cohort (n = 649; hgserver2.amc.nl). **K** Percentage of cell death in SH-SY5Y cells after 48 h pretreatment with MF-438 followed by 24 h RSL3 (5 µM) treatment (n = 3). **L** Percentage of cell death in SH-SY5Y cells pretreated with MF-438 (1 µM, 24 h) and then treated with RSL3 (5 µM, 24 h) following 72 h SCD1 siRNA transfection (n = 2). **M** Western blot confirming SCD1 knockdown via siRNA and protein levels of FASN, ACSL4 and GPX4. Data represented as mean ± SEM. Two-tailed unpaired T-test (**B**,**C**). Hotteling T2 test (**D**). Log-rank test (**G**, **I**). One-way ANOVA (**H**,**J**). Two-way Anova with Tukey’s multiple comparison (**K**, **L**). (*p ≤ 0.05, **p ≤ 0.01, ***p ≤ 0.001, ****p ≤ 0.0001). ACACA acetyl-CoA carboxylase, ACSL4 acyl-CoA synthetase long-chain family member 4, AKR1C aldo-keto reductase family 1C, CDX cell-derived xenograft, CoA coenzyme A, FASN fatty acid synthase, Fe iron pool modulating enzyme, FPN ferroportin, FSP1 ferroptosis suppressor protein 1, GCL glutamate cysteine ligase, GPX4 glutathion peroxidase 4, MUFA mono-unsaturated fatty acid, NFE2L2 or NRF2 nuclear factor erythroid 2-related factor, PL phospholipid, PUFA poly-unsaturated fatty acid, SCD1 stearoyl-CoA desaturase 1, SFA saturated fatty acid, SG sytox green.

Although NB has been classified as a ferroptosis-susceptible cancer type in a large-scale pharmacotranscriptomics screens [14], we observed heterogeneity in ferroptosis sensitivity across different high-risk NB models, both in vitro (Fig. 1A and Table S1) and in vivo (Figs. 1B, C and S1A–D). Notably, in vivo SH-SY5Y tumors displayed inconsistency in growth kinetics, with some tumors in both vehicle- and RSL3-treated groups showing minimal growth despite reaching the 100 mm³ threshold at treatment initiation. Intra-tumoral (I.T.) RSL3 (GPX4 inhibitor, class I ferroptosis inducer (FIN)) injection was required to induce ferroptosis in vivo, as intraperitoneal (I.P.) IKE (system X_c_^–^ inhibitor, class II FIN) administration did not yield consistent results (Fig. S1E, F), in contrast to prior findings [24].

To identify strategies to overcome ferroptosis-resistance, we performed a targeted compound screen focusing on enzymes involved in iron, redox and lipid metabolism (Table S2). SK-N-BE (2). C cells were selected as an intermediate ferroptosis-resistant model and not ferroptosis-sensitive IMR-32 nor SH-EP cells. Subsequent sensitization experiments were then carried out in SH-SY5Y cells, which represent the most resistant line. Hits (potential ferroptosis sensitizers) were identified based on increased lipid peroxidation and cell death following 24-h pretreatment prior to ferroptosis induction through IKE exposure (Fig. 1D). Of the top 10 validated hits, stearoyl-CoA 9-desaturase enzyme (SCD1) and nuclear factor erythroid 2-related factor (NRF2) emerged as the most potent ferroptosis sensitizing targets for both class I and class II ferroptosis inducing compounds (FINs) (Fig. 1E, Fig S2A and Tables S3 and S4).

SCD1 catalyzes the conversion of saturated fatty acids (SFAs) to mono-unsaturated fatty acids (MUFAs), thereby enriching membranes with non-oxidizable lipid species (Fig. 1F). This process mitigates lipid peroxidation and suppresses ferroptosis [17]. Kaplan-Meier analysis in the Kocak NB patient dataset revealed that high SCD1 expression is associated with poor over-all survival (Fig. 1G) and advanced disease stage (Fig. 1H), unlike NRF2, which showed a contrary association (Fig. 1I, J). Interestingly, high expression of ferroptosis regulators GPX4 and system X_c_^–^ subunit SLC3A2 was significantly associated with poor overall survival, whereas low expression of acyl-CoA synthetase long-chain family member 3 (ACSL3) correlated with poor outcome (Fig. S2B-G).

We further evaluated the effect of targeting de novo fatty acid synthesis enzymes (Fig. 1F), fatty acid synthase (FASN) and acetyl-CoA carboxylase (ACACA). Although high expression of these enzymes also correlated with worse prognosis (Fig. S2H-K), their inhibition failed to sensitize ferroptosis-resistant SH-SY5Y cells (Fig. S2L-M). In contrast, SCD1 inhibition robustly restored ferroptosis sensitivity in these resistant cells (Fig. 1K). siRNA-mediated SCD1 knock-down confirmed the on-target activity of inhibitor MF-438 (Fig. 1L), with a concomitant reduction of ACSL4, but unchanged GPX4 and FASN levels (Fig. 1M). As previously reported, we also observed some ferroptosis sensitization due to transfection procedure [25]. Collectively, these findings establish that inhibiting MUFA synthesis via SCD1 reverses ferroptosis resistance in these high-risk NB models.

### Arachidonic acid potentiates SCD1 inhibition to enhance ferroptosis

**Fig. 2:**
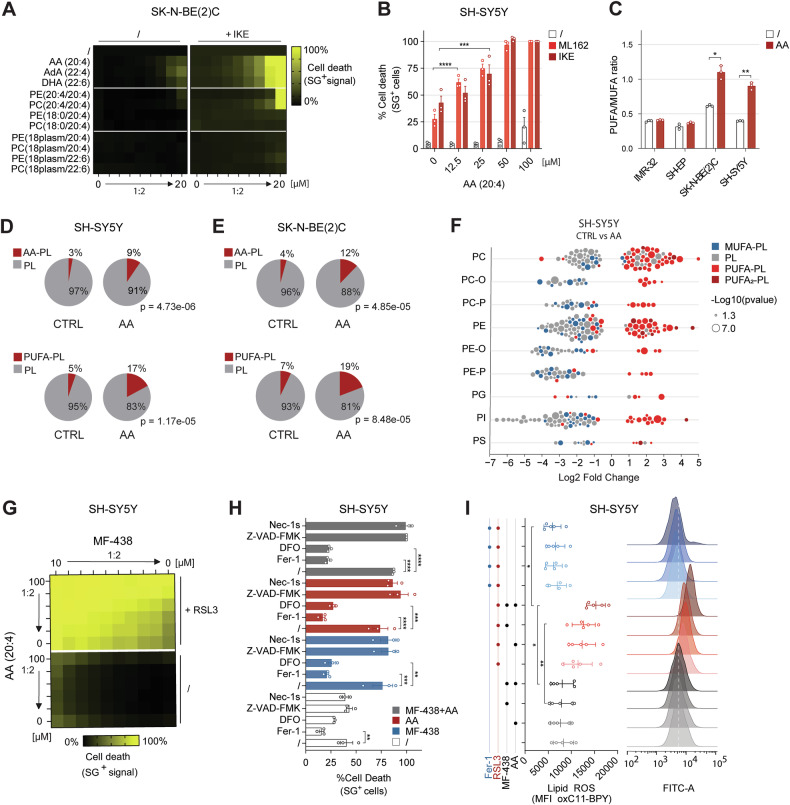
Dual phospholipid-unsaturation strategy by targeting SCD1 along AA supplementation strongly enhances ferroptosis responsiveness. **A** Heatmap presenting mean cell death percentage in SK-N-BE(2) C cells pretreated 72 h with PUFAs or PUFA-(e)PLs, followed by 24 h IKE (10 µM) in 2% FBS media conditions (n = 3; Table S5). **B** Cell death (%) induced in SH-SY5Y cells after 72 h AA pretreatment, followed by 24 h ML162 (0.5 µM, n = 3) or IKE (20 µM, n = 3). **C–E** PUFA/MUFA ratio, PL-AA or PL-PUFA (unsaturation ≥4) represented as % of phospholipids, in high-risk NB cells treated 72 h with vehicle or AA (1.25 µM in IMR-32, 5 µM in SH-EP, 5 µM in SK-N-BE(2) C, 20 µM in SH-SY5Y) in 2% FBS (n = 3). **F** Log2 fold change of individual lipid species after 72 h vehicle or 20 µM AA treatment at 2% FBS media conditions. **G** Heatmap showing mean cell death (%) induced after combination pretreatment of AA and MF-438 at different concentrations in SH-SY5Y cells, followed by 24 h RSL3 (5 µM, n = 3, Table S10) exposure. **H** Cell death (%) induced in SH-SY5Y cells, 48 h pretreated with AA (20 µM), MF-438 (1 µM) or their combination and the corresponding cell death inhibitor Ferrostatin- 1 (Fer-1, 10 µM), DFO (50 µM), Z-VAD-FMK (10 µM) or Nec-1s (10 µM), followed by 24 h RSL3 (4 µM) (n = 3). **I** Median fluorescent intensity (MFI) and corresponding histogram of oxidized BODIPY 581/591 C11 (oxC11-BPY) measured in the FITC-A channel, in SH-SY5Y cells after 48 h pretreated with AA (20 µM), MF-438 (1 µM) or their combination followed by 1 h Fer-1 (10 µM) and subsequent 24 h RSL3 (4 µM) treatment (n = 5). Data represented as mean ± SEM. Two-way ANOVA with Dunnett’s multiple comparison (**B**). Two-tailed unpaired T-test (**C**–**F**). One-way Anova with Dunnet’s multiple comparison (**H**). One-way Anova with Tukey’s multiple comparison (**I**). (*p ≤ 0.05, **p ≤ 0.01, ***p ≤ 0.001, ****p ≤ 0.0001). AA arachidonic acid, AdA adrenic acid, CTRL control, DFO deferoxamine, DHA docosahexaenoic acid, Fer-1 Ferrostatin-1, MUFA mono-unsaturated fatty acid, PC phosphatidylcholine, PC-O 1-alkyl,2-acylphosphatidylcholine, PC-P 1-alkenyl,2-acylphosphatidylcholine, PE phosphatidylethanolamine, PE-O 1-alkyl,2-acylphosphatidylethanolamine, PE-P 1-alkenyl,2-acylphosphatidylethanolamine, PG phosphatidylglycerol, PI phosphatidylinositol, PL phospholipid, PUFA poly-unsaturated fatty acid, PUFA_2_-PL phospholipid containing two poly-unsaturated fatty acids, PS phosphatidylserine, SG sytox green.

Given that poly-unsaturated fatty acid (PUFA) supplementation enhances ferroptotic cell death by enriching membranes with oxidizable lipids [26, 27], we hypothesized that cotreatment with PUFAs might intensify the effect of SCD1 inhibition. Indeed, free PUFAs—particularly arachidonic acid (AA; C20:4)—potently sensitized cells to ferroptosis, whereas PUFA-containing phospholipids (PUFA-PLs) and ether-linked PUFA-PLs had limited effects (Fig. 2A, B, Fig. S3 and Table S5–S9). Lipidomics revealed a significant increase in the PUFA/MUFA ratio following AA treatment (Fig. 2C), driven by increased PUFA-PLs (Fig. 2D, E) and decreased MUFA-PLs in resistant cell lines (Fig. 2F and Fig. S4A-C). A PUFA-PL increase was also observed in SH-EP cells, but not in IMR-32 cells (Fig. S4D-G). Lipid supplementation experiments were performed in 2% FBS to minimize background serum lipids, while avoiding dialyzed FBS, which also depletes amino acids, hormones, and antioxidants that influence ferroptosis. Under serum-reduced conditions, cells were more vulnerable to ferroptosis inducers, as 20 µM IKE and 0.5 µM ML162 induced ~50% and ~25% cell death (Fig. 2B), respectively, compared with no cell death at 10% FBS (Fig. 1A). Importantly, AA retained its sensitizing potential even after shorter pretreatments (24–48 h) and under lipid-rich conditions (10% FBS) (Figure S5).

Combining AA supplementation with SCD1 inhibition yielded a dose-dependent enhancement of ferroptosis in the most resistant SH-SY5Y cells (Fig. 2G and Table S10), surpassing effects observed with inhibitors of FASN, ACACA, or FSP1 (Fig. S6 and Tables S11–S15). Notably, under serum reducing conditions de novo FA synthesis inhibitors sensitized SK-N-BE (2). C cells together with AA supplementation, but not with SCD1 inhibition (Figure S7). Cell death induced by RSL3 in SH-SY5Y cells pretreated with MF-438, AA, or their combination was rescued by ferroptosis inhibitors (Ferrostatin-1 and deferoxamine), but not by apoptosis or necroptosis inhibitors (Z-VAD-FMK or Nec-1s) (Fig. 2H). Cell death was accompanied by increased lipid peroxidation, as shown by C11-BODIPY oxidation, which was suppressed by Fer-1 (Fig. 2I). These results establish PUFA supplementation, especially with AA, as a powerful strategy to synergize with MUFA synthesis blockade in ferroptosis-resistant NB cells.

### Lipidome and transcriptome profiles predict ferroptosis sensitivity in high-risk NB

**Fig. 3:**
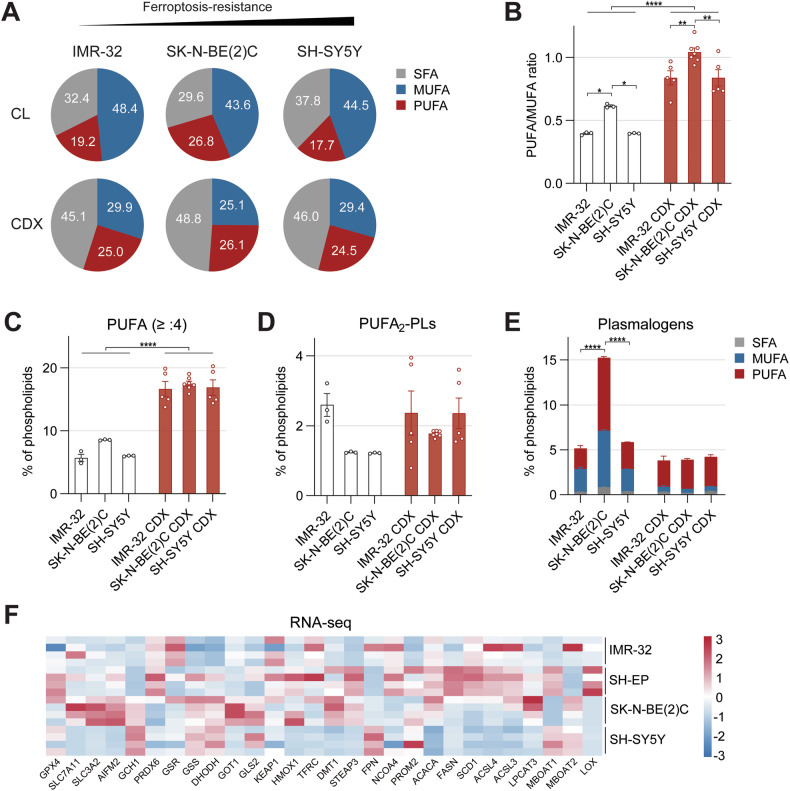
Ferroptosis sensitivity is not reflected in the lipidome or transcriptome of NB models. **A** Distribution of lipid unsaturation in untreated high-risk NB cell lines (CL) and matched CDX tumors as percentage of total phospholipids. The PUFA/MUFA ratio (**B**), phospholipids with unsaturation ≥4 (**C**) and PUFA_2_-PLs (**D**) presented as percentage of total phospholipids in white for high-risk NB cell lines and red for the matched CDX tumor samples. **E** The percentage of plasmalogens (alkenyl-ether-linked phospholipids, PL-P) of the total phospholipids detected, including the distribution based on saturation (SFA, MUFA, PUFA) in high-risk NB cell lines and matched CDX tumor. **F** Expression levels (Log2 FC) of key ferroptosis players between all four cell lines (n = 4). For all lipidomics experiments: n = 3 for cell lines, n = 5 for IMR-32 CDX, n = 7 for SK-N-BE (2). C CDX, n = 5 for SH-SY5Y CDX). Data represented as mean ± SEM. Two-way Anova with Tukey’s multiple comparison. (*p ≤ 0.05, **p ≤ 0.01, ***p ≤ 0.001, ****p ≤ 0.0001). CL cell line, CDX cell-derived xenograft, MUFA mono-unsaturated fatty acid, PUFA poly-unsaturated fatty acid, PUFA_2_-PL phospholipid containing two poly-unsaturated fatty acids, SFA saturated fatty acid.

To understand how intrinsic lipid compositions influence ferroptosis response, we performed comparative lipidomics across NB cell lines and their corresponding cell-derived xenograft (CDX) tumors in vivo. This revealed consistent differences between in vitro and in vivo models (Fig. S8 and 9), with CDX tumors exhibiting higher SFA content and significantly elevated PUFA/MUFA ratios (Fig. 3A, B). Note that SH-EP CDX tumors could not be established. Additionally, PLs with ≥4 double bonds and PLs containing two PUFAs (PUFA_2_-PLs) were enriched in CDXs (Fig. 3C, D and Fig. S10). Ether-linked PUFA-PLs, more specifically the alkenyl-ether-linked plasmalogens (PL-P), were similarly enriched (Fig. 3E and Fig. S11). Yet none of these lipidomic features reliably predicted ferroptosis sensitivity. Likewise, transcriptomic analysis of the four cell lines revealed no clear, consistent correlation between expression of key ferroptosis-regulating genes and sensitivity (Fig. 3F), indicating a dissociation between lipidome saturation state, RNA-expression profiles, and ferroptotic potential in high-risk NB.

### LNP-based delivery of AA enhances in vivo applicability and tumor targeting

**Fig. 4:**
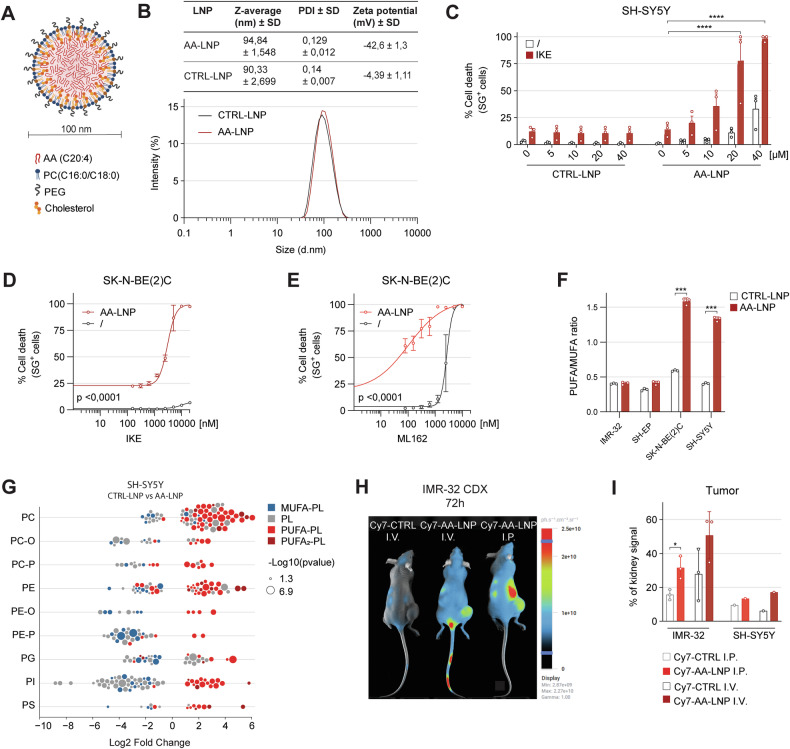
AA-LNP enhances ferroptosis by promoting PUFA incorporation and exhibits tumor targeting properties in IMR-32 CDX models. **A** Visual representation of AA-LNP and its components. **B** Summarizing table of the biophysical properties and intensity-based size distribution curves of AA-LNP and CTRL-LNP formulations (technical n = 3). **C** Cell death (%) induced in SH-SY5Y cells pretreated with CTRL-LNP or AA-LNP for 72 h, followed by 24 h IKE (20 µM) treatment at 2% FBS media conditions (n = 3). **D–E** Cell death percentage in SK-N-BE (2) C cells pretreated 72 h with 10 µM AA-LNP, followed by 24 h treatment of RSL3 or ML162 at 2% FBS media conditions (n = 2). **F** PUFA/MUFA ratio’s in high-risk NB cells treated 72 h with CTRL-LNP or AA-LNP (1.25 µM in IMR-32, 5 µM in SH-EP, 10 µM in SK-N-BE (2). C, 20 µM in SH-SY5Y) in 2% FBS (n = 3). **G** Log2 fold change of individual lipid species in SH-SY5Y cells comparing 72 h CTRL-LNP versus AA-LNP (20 µM AA) treatment at 2% FBS media conditions (n = 3). **H** In vivo fluorescence images of IMR-32 CDX mice, 72 h post-injection with non-encapsulated 18:1 Cy7-PE (I.V., Cy7-CTRL) or Cy7-AA-LNP (I.P. or I.V.). **I** Ex vivo quantification of tumor fluorescence (normalized to kidney signal) in IMR-32 (n = 3) and SH-SY5Y (n = 1) CDX mice. Data represented as mean ± SEM, except for SH-SY5Y tumor fluorescence: mean ± SD (**I**). Two-way Anova with Dunnett’s multiple comparison (**C**). Two-way Anova with Fisher’s LSD multiple comparison, showing p value of comparison at 1.25 µM trigger (**D**, **E**). Two-tailed unpaired T-test (**F**, **G**, **I**). (*p ≤ 0.05, **p ≤ 0.01, ***p ≤ 0.001, ****p ≤ 0.0001). AA arachidonic acid, AA-LNP arachidonic acid-loaded lipid nanoparticle, CTRL control, CTRL-LNP control lipid nanoparticle, LNP lipid nanoparticle, MUFA mono-unsaturated fatty acid, PC phosphatidylcholine, PC-O 1-alkyl,2-acylphosphatidylcholine, PC-P 1-alkenyl,2-acylphosphatidylcholine, PDI polydispersity index, PE phosphatidylethanolamine, PEG polyethylene glycol, PE-O 1-alkyl,2-acylphosphatidylethanolamine, PE-P 1-alkenyl,2-acylphosphatidylethanolamine, PG phosphatidylglycerol, PI phosphatidylinositol, PL phospholipid, PUFA poly-unsaturated fatty acid, PUFA_2_-PL phospholipid containing two poly-unsaturated fatty acids, PS phosphatidylserine, SG sytox green.

AA is hydrophobic and insufficiently soluble in water for pharmaceutical application. To overcome the solubility limitations of free AA and enable in vivo application, we developed an AA-loaded lipid nanoparticle (AA-LNP) formulation (Fig. 4A). This approach avoided the use of toxic solvents (Fig. S12) and incorporated phospholipids and cholesterol, yielding particles with uniform size (95 ± 10 nm, PDI < 0.3) (Fig. 4B). Fluorescently Cy5-labeled AA-LNPs confirmed cellular uptake (Fig. S13), and AA-LNPs dramatically increased ferroptosis sensitivity in resistant cells (Fig. 4C–E and Fig. S14). LNP formulations of AA-containing PLs were also developed but showed no strong ferroptosis-sensitization (Fig. S15).

AA-LNPs more effectively elevated the PUFA/MUFA ratio (Fig. 4F) and modulated lipid composition (Fig. 4G and Fig. S16A-C). The effects were consistent in SH-EP but not IMR-32 cells (Fig. S16D-G). Importantly, following I.V. and I.P. injections, Cy7-labeled AA-LNPs accumulated selectively in IMR-32 CDX tumors, considering the control Cy7-linked phospholipid signal in non-tumor organs. Tumor targeting effect was absent in SH-SY5Y tumors (Fig. 4H, I and Fig. S17), suggesting enhanced permeability and retention (EPR) effect-mediated targeting is model-dependent.

### Combination therapy of AA-LNP and SCD1 inhibition induces in vivo ferroptosis sensitization

**Fig. 5:**
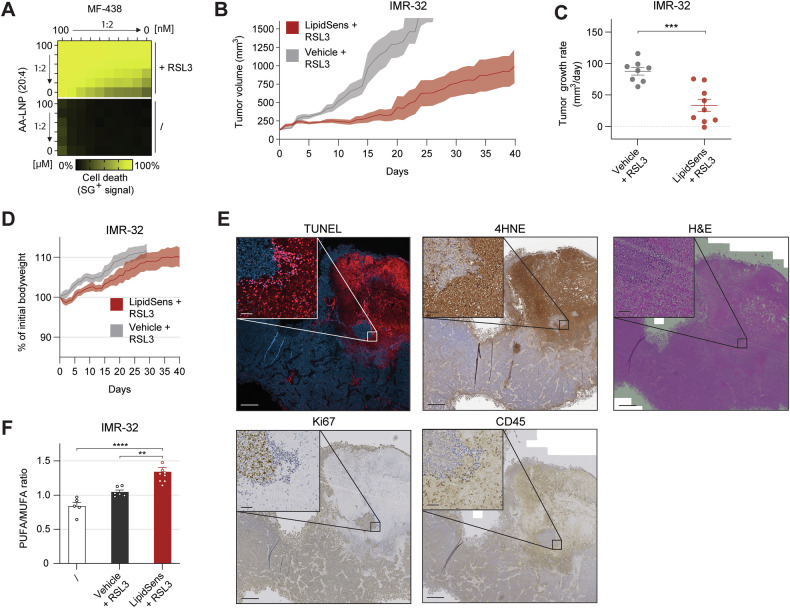
AA-LNP and SCD1 targeting treatment increases unsaturation and enhances ferroptosis response in IMR-32 CDX tumors. **A** Heatmap representing mean cell death (%) induced after combination pretreatment of AA-LNP and MF-438 at different concentrations in SH-SY5Y cells, followed by 24 h RSL3 exposure (2.5 µM, n = 3, Table S16). **B–D** Tumor volume (mm3), tumor growth rate (mm^3^/day) and % of initial bodyweight of IMR-32 CDX mice treated with I.T. vehicle (n = 8, 10%DMSO in DPBS) or LipidSens (n = 9, Cy7-AA-LNP in DPBS containing 13.2 mM AA + 0.5 mM MF-438 reaching 10% DMSO) alternated daily with I.T. RSL3 injections. **E** Representative sections of best-responding LipidSens + RSL3–treated IMR-32 CDX tumor, showing TUNEL staining for DNA fragmentation, 4HNE immunohistochemistry staining for lipid peroxidation product deposits, Ki67 immunohistochemistry for proliferation, CD45 immunohistochemistry for immune-cell infiltration, and H&E staining. Scale bar 500 µm and zoom section 50 µm**. F** PUFA/MUFA ratio in untreated (/, n = 5), vehicle+RSL3 (n = 8) or Lipidsens+RSL3 (n = 9) treated IMR-32 CDX tumors. Data represented as mean ± SEM. Two-tailed unpaired T-test (**C**). Two-way ANOVA with Tukey’s multiple comparison (**F**). (*p ≤ 0.05, **p ≤ 0.01, ***p ≤ 0.001, ****p ≤ 0.0001). 4HNE 4-hydroxynonenal, AA-LNP arachidonic acid-loaded lipid nanoparticle, CD45 cluster of differentiation 45, H&E hematoxylin and eosin, I.T. intratumoral injection, LipidSens dual lipid-modifying treatment combining AA-LNP and MF-438, MUFA mono-unsaturated fatty acids, PUFA poly-unsaturated fatty acids, SCD1 stearoyl-CoA desaturase 1, SG sytox green, TUNEL terminal deoxynucleotidyl transferase dUTP nick end labeling.

Combining AA-LNP with SCD1 inhibitor MF-438 displayed a stronger sensitization in ferroptosis-resistant NB cells (Fig. 5A and Table S16). Next, we evaluated this dual-sensitization strategy—termed LipidSens—in vivo using IMR-32 CDX tumors, with the aim to further enhance the response to RSL3. Intratumoral co-treatment of AA-LNP and MF-438, alternating with RSL3, significantly inhibited tumor growth without adverse effects (Fig. 5B–D). Tumor sections from responders showed significant regions of cell death (TUNEL+), accumulation of the lipid peroxidation product 4-hydroxynonenal (4HNE), reduced proliferation (Ki67), and increased immune cell infiltration (CD45) (Fig. 5E and Fig. S18). Lipidomic analysis confirmed increased PUFA/MUFA ratio in treated tumors, mirroring in vitro findings (Fig. 5F and Fig. S19A-F). Epilipidomic profiling detected only few significantly altered oxidized phospholipids; however, increased oxidation was observed in two CDX samples following combination therapy (Fig. S19G). In contrast, SH-SY5Y CDX tumors remained unresponsive, potentially due to lower SCD1 expression in vivo (Fig. S20). Together, these results demonstrate that AA-LNP combined with SCD1 inhibition amplifies ferroptosis sensitivity in RSL3-responsive high-risk NB model.

## Discussion

Ferroptosis is a promising therapeutic avenue for treating aggressive and therapy-resistant cancers, including high-risk NB [1–3]. However, resistance to ferroptosis remains a major limitation [28]. Our study presents a dual-targeted lipid remodeling strategy, combining MUFA synthesis inhibition (via SCD1) with exogenous PUFA supplementation (via AA-LNPs), to overcome ferroptosis resistance.

SCD1 inhibition sensitized multiple high-risk NB models to ferroptosis, consistent with prior reports showing the ferroptosis-protective role of SCD1 in other malignancies [29–31]. Meanwhile, AA supplementation further potentiated ferroptosis by enriching membranes with oxidizable lipids. Importantly, PUFA-containing PL subclasses such as PUFA_2_-PLs and ether-linked PLs did not confer sensitivity in high-risk NB, in contrast to other cancer types [27, 32], highlighting context-specific lipid dependencies.

Mechanistically, high-risk NB cells rely on de novo FA synthesis to support rapid proliferation. However, the absence of Δ12-desaturase in mammalian cells prevents endogenous PUFA production and leads to SFA- and MUFA-enriched membranes. SCD1 inhibition shifts lipid composition away from MUFAs, sensitizing membranes to lipid peroxidation upon PUFA uptake—especially AA. This interplay underlies the synergistic effect of combined SCD1 blockade and AA supplementation.

Although inhibition of other FA synthesis enzymes (e.g., FASN, ACACA) show anti-cancer effects [19, 33, 34], even in context of ferroptosis [35, 36], their effects in high-risk NB were limited and context-dependent, particularly in vitro. However, the elevated SFA content in NB CDX tumors suggests that these inhibitors may be more effective in vivo.

To enable PUFA delivery in vivo, we developed an AA-loaded lipid nanoparticle (AA-LNP). AA-LNPs overcame solubility limitations, exhibited tumor-specific accumulation via the EPR effect, and significantly enhanced ferroptosis sensitivity when combined with SCD1 inhibition. However, inter-tumor variability in nanoparticle delivery remains a limitation, as SH-SY5Y CDX tumors showed no AA-LNP accumulation. This variability likely reflects differences in tumor vascularization and blood flow as the EPR effect relies on the accumulation of larger-sized nanoparticles in tumor tissue because of leaky vasculature and impaired lymphatic drainage [37, 38]. Further optimizations of our modular LNP design could improve therapeutic efficacy and facilitate clinical translation in high-risk NB. Integrating tumor-targeting moieties, such as disialoganglioside (GD2)-directed nanobodies, may enhance specificity and tumor accumulation, including at metastatic sites. Note, anti-GD2 antibodies are already part of immunotherapy regimens for high-risk NB patients [39–41].

Additional lipidomic analyses revealed substantial shifts in lipid composition during in vivo tumor establishment. The phospholipid unsaturation profiles observed in vitro did not correlate with ferroptosis sensitivity, and differences in lipid unsaturation became minimal across established CDX tumors. Notably, lipids containing ≥4 double bonds, including PUFA_2_-PLs, were significantly enriched in vivo but did not align with the ferroptosis-sensitivity profiles observed in our high-risk NB models [27, 32]. A similar pattern was seen for PUFA-containing alkenyl-ether-linked phospholipids (plasmalogens), despite a previous study linking their enrichment to increased ferroptosis vulnerability [42]. Together with the transcriptomics data, these findings suggest that ferroptosis susceptibility cannot be reliably estimated from transcriptomics and lipidomic profiles alone, as gene expression levels and lipid compositions are dynamically remodeled during tumor progression and may not directly predict therapeutic response. In addition, endogenous radical-trapping mechanisms—such as vitamin E, vitamin K hydroquinone, BH4, and reduced CoQ10—can override intrinsic ferroptosis sensitivity. Thus, a more comprehensive approach that integrates targeted metabolomics with other omics layers will likely be required to delineate the complex ferroptosis “fingerprint” [43].

Despite these complexities, our study is the first to demonstrate in vivo anti-tumor efficacy of a bidirectional lipid-modulating strategy that enhances ferroptosis via simultaneous inhibition of monounsaturated fatty acid (MUFA) synthesis and enrichment of polyunsaturated fatty acids (PUFAs). Prior studies have reported anti-cancer effects of α-eleostearic acid (C18:3) [44] and SCD1 inhibition in ovarian [29] and gastric cancer models [45], including in combination with erastin. However, our findings provide the first in vivo foundation for ferroptosis-potentiating nanomedicines based on dual lipid modulation. While RSL3 was used to induce ferroptosis and 4HNE was detected in responder tumors, it remains challenging to definitively attribute tumor regression to ferroptotic cell death in vivo. Future preclinical studies, ideally including a ferroptosis inhibitor rescue group, will be important to establish this more conclusively.

Furthermore, co-encapsulation of SCD1 inhibitors with other ferroptosis-inducing agents could enhance therapeutic efficacy, although this approach will require extensive formulation efforts. While clinically approved nanomedicines such as liposomal doxorubicin exist [46], none are currently indicated for high-risk NB. Albumin-bound paclitaxel nanoparticles (Nab-PTX) have shown limited efficacy in NB clinical trials [47]. Our strategy offers a flexible platform for advancing ferroptosis-based nanotherapies, allowing tumor-specific delivery through surface functionalization with disease-relevant ligands.

In conclusion, our lipid-sensitization strategy overcomes ferroptosis resistance by reprogramming membrane lipid compositions, thereby unlocking the therapeutic potential of ferroptosis in high-risk NB. Future work should address tumor heterogeneity in LNP delivery and explore combination strategies with immunotherapy or chemotherapy to further improve therapeutic efficacy.

## Supplementary information

Supplementary Data

Supplementary Western Blots

## Data Availability

The RNA-seq data generated during this study have been deposited in the European Nucleotide Archive (ENA) under accession number ERP180857 (https://www.ebi.ac.uk/ena/browser/view/ERP180857).
