# Supplementary material for: Dual lipid modulation overcomes ferroptosis resistance in high-risk neuroblastoma
Source: Cell Death Differ. 2025 Nov 26;33(5):903–13. doi: 10.1038/s41418-025-01623-3 (PMC13156318; doi:10.1038/s41418-025-01623-3)
Supplement: Supplementary file 1 — Supplementary Data [file 41418_2025_1623_MOESM1_ESM.docx]

Supplementary Information for

**Dual Lipid Modulation Overcomes Ferroptosis Resistance in High-Risk Neuroblastoma**

Ine Koeken, Magali Walravens^#^, Roberto Fernández-Acosta^#^, Ruben Van Hoyweghen, Iuliana Vintea, Yingy Kong, Bianka Golba, Jonas Dehairs, Ali Talebi, Johannes V. Swinnen, Kaat Durinck, Adriana Mañas, Shinya Toyokuni, Gerben Menschaert, Maria Fedorova, Bruno G. De Geest, Behrouz Hassannia, Tom Vanden Berghe*

* Corresponding author: Tom Vanden Berghe, tom.vandenberghe@uantwerpen.be

^#^ These authors contributed equally to this work.

**This file includes**

[Supplementary Methods 2](#_Toc211513620)

[Supplementary Figures 10](#_Toc211513621)

[Supplementary Tables 41](#_Toc211513622)

[Epilipidomics Inclusion List 66](#_Toc211513623)

[References 73](#_Toc211513624)

# Supplementary Methods

*Cell Death analysis*

Cell death was quantified by measuring SytoxGreen fluorescence intensity using the FLUOstar Omega plate reader (BMG Labtech GmbH, Germany) as previously described (1) . Briefly, cells were seeded in 96-well plates and pretreated with the desired lipid and/or compound for 24, 48, or 72 hours. For cell death rescue experiments, inhibitors were co-administered during the pretreatment phase at the concentrations specified in the figure legends. Note, some experiments were performed in 2% FBS media instead of 10% conditions, indicated in figure legends. Subsequently, a ferroptosis inducer was added at the desired concentrations in the presence of SytoxGreen (1.7 µM). After 24 hours of induction, fluorescence intensity was measured with excitation/emission filters of 485/520 nm. Each experimental condition was performed in triplicate. To establish 100% cell death, wells were treated with either a toxic concentration of the ferroptosis inducer or 0.05% Triton X-100 (at least 3 wells per experiment). Background fluorescence was determined using untreated wells (at least 3 wells per experiment). The percentage of the cell death was calculated by the formula (avg. SytoxGreen [well of interest]­ – avg. SytoxGreen [background]) /(avg. SytoxGreen [Toxic concentration inducer or Triton X-100] – avg. SytoxGreen [background])× 100. The graphs were plotted in GraphPad Prism 10. The following lipids were used as pretreatment: arachidonic acid (A3611, Sigma-Aldrich), adrenic acid (D3659, Sigma-Aldrich), docosahexaenoic acid (D2534, Sigma-Aldrich), PE(20:4/20:4) (850800C, Avanti Polar Lipids), PE(18:0/20:4) (850804C, Avanti Polar Lipids), PE(18plasm/20:4) (852804C, Avanti Polar Lipids), PE(18plasm/22:6) (852806C, Avanti Polar Lipids), PC(20:4/20:4) (850397C, Avanti Polar Lipids), PC(18:0/20:4) (850397C, Avanti Polar Lipids), PC(18plasm/20:4) (852469C, Avanti Polar Lipids) and PC(18plasm/22:6) (852472C, Avanti Polar Lipids). Ferroptosis stimuli that were used: RSL3 (HY-100218A, MedChemExpress), ML162 (S4452, Selleckchem), IKE (S8877, Selleckchem) and FINO2 (25096, Cayman Chemicals). Cell death inhibitors used in rescue experiments: Ferrostatin-1 (S7243, Selleckchem), deferoxamine (D9533-1G, Sigma-Aldrich), Z-VAD-FMK (HY-16658B, MedchemExpress) and Nec-1s (HY-14622A, MedchemExpress). For information on compounds used as pretreatment see Table S2. Note, iFSP1, viFSP1 and ciFSP1 were kindly gifted by Marcus Conrad Laboratory.

*LNP formulation*

Lipid nanoparticles (LNPs) were formulated by solvent displacement involving the addition of an ethanolic lipid solution to an aqueous solution of 10 mM acetate buffer (pH 4) at a volume ratio of 1:2 during high-speed vortex mixing. The ethanol solution of AA-LNP comprised arachidonic acid (AA) (A3611, Sigma-Aldrich), PC(16:0/18:1) (850457P, Avanti Polar Lipids), cholesterol (C3045, Sigma-Aldrich), and DMG-PEG (80151, Avanti Polar Lipids) at a mol% ratio of 50:10:38:2. The low pH was selected to avoid deprotonation of AA, increasing its hydrophobicity and loading in LNP. For preparation of control LNP (CTRL-LNP), the ethanol solution contained PC(16:0/18:1), cholesterol, and DMG-PEG at a mol% ratio of 68:30:2. To prepare fluorescently labelled LNPs, Cy5-AA-LNP and Cy7-AA-LNP, the AA-LNP formulation was supplemented with 0.1 mol% of 18:1 Cyanine 5 PC (850483, Avanti Polar Lipids) or 18:1 Cyanine7 PE (810337C, Avanti Polar Lipids), respectively. This resulted in a final mol% ratio of AA, PC(16:0/18:1), cholesterol, DMG-PEG, and fluorescently labeled lipid at 50:9.9:38:2:0.1. Following formulation, ethanol was removed from the LNP formulation by dialysis against MilliQ water or Dulbecco's phosphate-buffered saline (DPBS) using Slide-A-Lyzer cassettes (cut-off 3.5 kDa, 66333, Thermo Fisher). LNP formulations exceeding 3 mL were dialyzed using Spectra/Por dialysis membranes (cut-off 3.5 kDa, 131720). For *in vivo* experiments, AA-LNP solutions were concentrated 4-fold using Vivaspin Turbo PES tubes (cut-off 100 kDa, VS15T41, Sartorius). Particle size and polydispersity index (PDI) were determined using dynamic light scattering (DLS) with a Zetasizer Nano ZS (Malvern Instruments Ltd, Malvern, UK). The LNP samples were diluted 100-fold in 20 mM HEPES buffer (pH 7.4) and measured in triplicate. Zeta potential measurements were performed similarly, but with a 20-fold dilution instead of 100-fold. All measurements were conducted at room temperature, and each sample was measured in triplicate to ensure accuracy. LNP formulations were stored at 4°C.

*siRNA knock-down*

SH-SY5Y cells were seeded at a density of 15000 cells per well in a 12-well plate. After 24 hours, cells were transfected with 10 nM human SCD1 siRNA (siSCD1, 4390824 s12505, Invitrogen) or negative control siRNA (siNeg, 4390843, Invitrogen) using Lipofectamine® RNAiMAX Reagent (13778-150, Invitrogen), following the manufacturer's protocol (MAN0007825 Rev1.0). Transfection was carried out for 72 hours. Following transfection, cells were collected and reseeded in parallel: one set in a 12-well plate for knockdown validation by Western blot, and another set in a 96-well plate for cell death analysis.

*Flow cytometry measurements*

SH-SY5Y cells were seeded in 6-well plates. The following day, arachidonic acid (AA, 20 µM), MF-438 (1 µM), or their combination was added as pretreatment. After 48 h, cells were exposed to the ferroptosis inhibitor Ferrostatin-1 (10 µM, 1 h), followed by RSL3 (4 µM, 4 h). Fifteen minutes before collection, BODIPY 581/591 C11 (D3861, Thermo Fisher) was added at a final concentration of 2 µM. Cells were then trypsinized and resuspended in 2.5 mL medium containing Draq7 (0.3 µM). Flow cytometry was performed using a BD Accuri™ C6 Plus (BD Biosciences, USA). Live (Draq7-negative) cells were selected using the 640 nm laser and 675/25 filter, and oxidized C11-BODIPY was detected using the 488 nm laser and 533/30 filter. Data were analyzed with FlowJo software (version 10.9.0).

*Functional compound screen*

A custom-designed compound set of 33 compounds, was screened to identify ferroptosis-sensitizing compounds at the VIB Screening Core. SK-N-BE(2)C cells were seeded in 384-well phenoplates (6057302, Revvity) at a density of 3500 cells per well for the 1-hour pretreatment condition and 2500 cells per well for the 24-hour pretreatment condition. Cells were pretreated with each compound (Table S2) at two concentrations (1 µM and 10 µM) for either 1 hour or 24 hours. Subsequently, cells were treated with either IKE (3.5 µM, S8877, Selleckchem) or an equivalent volume of DMSO (0.0175%) as the untreated condition. Additionally, IKE-only and DMSO-only control wells were included to assess baseline responses. Together with IKE or DMSO, fluorescent dyes were added: C11-BODIPY581/591 (1 µM, D3861, Invitrogen) to measure lipid peroxidation and DRAQ7 (0.3 µM, DR71000, BioStatus) to detect dead cells. Each well was imaged every hour for 20 hours using the Opera Phenix HCS system (Revvity, USA) with 16 fields acquired per well in 3 planes at 20× water immersion objective. Images were processed using the Columbus Plus digital platform (Revvity, USA) to quantify the percentage of DRAQ7-positive cells and the oxidized C11-BODIPY intensity in living (DRAQ7-negative) cells.

*Western Blot*

Cells were lysed in RIPA buffer supplemented with protease (cOmplete™ Protease Inhibitor Cocktail, 04693116001, Roche) and phosphatase inhibitors (PhosSTOP, 04906845001, Roche). Tumor tissue was homogenized in Caspase lysis buffer (4 mM Tris-HCl, 200 mM NaCl, 10% glycerol, 1% Igepal, 5 mM EDTA) supplemented with aprotinin (0.15 µM, A1153, Sigma-Aldrich), PMSF (0.1 mM, P7626, Sigma-Aldrich), and leupeptin (2.1 µM, L2884, Sigma-Aldrich) using the Tissue Lyzer II (Qiagen, Netherlands). Samples were denatured by boiling in Laemmli buffer for 10 minutes. Proteins were separated by SDS-PAGE and transferred to nitrocellulose membranes (1620115, Bio-Rad) using wet transfer. Membranes were blocked in 5% non-fat dry milk in TBST (TBS with 0.05% Tween-20) and incubated overnight at 4°C with primary antibody against SCD1 (1:1000, ab236868, Abcam), FASN (1:1000, 3180S, Cell Signaling Technology), ACSL4 (1:10000, AB155282, Abcam) or GPX4 (1:1000, AB125066, Abcam). After washing, membranes were incubated for 1 hour at RT with polyclonal swine anti-rabbit IgG HRP-conjugated secondary antibody (1:5000, P0399, Dako). β-tubulin was used as a loading control and detected using an HRP-conjugated β-tubulin antibody (1:10000, BT7R, MA5-16308-HRP, Invitrogen) for 1 hour at RT. Detection was performed using Clarity Western ECL substrate (1705060, Bio-Rad), and signals were visualized using the Chemidoc Imaging System (Bio-Rad, USA).

*Immunohistochemistry*

Tumors were dissected and fixed in 4% formaldehyde for 24 hours, followed by transfer to 70% isopropanol for paraffin embedding. Sections (4–5 µm) were cut, dewaxed, and subjected to heat-mediated antigen retrieval using a citrate-based antigen unmasking solution (H-3300, Vector Laboratories). Non-specific binding sites were blocked with normal goat serum (20% in TBST, P30-1001, Pan Biotech) for 20 min at room temperature. Primary antibodies for Ki67 (1:1000, 4°C, 12202, Cell Signaling Technology) or CD45 (1:2000, RT, ab10558, Abcam) were incubated overnight. Endogenous peroxidase activity was suppressed with a 0.9% hydrogen peroxide solution, followed by incubation with biotinylated goat-anti-rabbit secondary antibody (PK-4001, Vector Laboratories). The avidin/biotinylated peroxidase complex (VECTASTAIN ABC kit, Vector Laboratories) was applied, and peroxidase activity was detected using diaminobenzidine (DAB) as the substrate. Slides were counterstained with hematoxylin for 10 min and mounted. Images were acquired using a Axioscan Z1 automated whole slide scanner (Zeiss, Oberkochen, Germany) with a 20x objective lens (Plan-Apochromat, N.A. 0.8) (pixel size: 0.22 x 0.22 µm). The 4-hydroxynonenal (4-HNE) staining was performed with anti-4HNE-modified proteins monoclonal antibodies HNEJ-1(2–4) by the fully automated staining system BOND MAX/III (Leica, Wetzlar, Germany) using BOND Polymer Refine Detection (ds9800; Leica) as described. Whole slide images were analyzed with QuPath (version 0.5.1) by manually delineating tissue regions and quantifying DAB-positive cells relative to total hematoxylin-stained nuclei for Ki67 or tissue area in case of CD45 and 4HNE.

*TUNEL assay*

Tumor slides were prepared as described in the Immunohistochemistry section. TUNEL assay was performed by following the manufacturer’s protocol of the *in-situ* cell death detection kit (TMR-red, Roche). The nuclei were counterstained with Hoechst, mounted and scanned with an Axio Scan Z1 using a 20x objective lens (Plan-Apochromat, N.A. 0.8) and Colibri 7 solid-state light source for excitation of Hoechst (385 nm) and TUNEL (590 nm). Whole slide images were analyzed with QuPath (version 0.5.1) by manually delineating tissue regions and quantifying TUNEL-positive cells relative to the total number of Hoechst-positive nuclei.

*Lipidomics*

Lipidomics on cell pellets and tumor tissue was performed as previously described by Talebi et al. (2023). Briefly, 700 µL of homogenized cells or tissue was mixed with 800 µL 1 N HCl:CH3OH (1:8, v/v), 900 µL CHCl3, 200 µg/mL of the antioxidant 2,6-di-tert-butyl-4-methylphenol, and 3 µL of Ultimate SPLASH™ ONE internal standard mix (330820, Avanti Research). The organic fraction was collected, evaporated, and the lipid pellet was stored at -20 °C under argon. For the lipidomics analysis, lipid pellets were reconstituted in 100% ethanol and analyzed by LC-ESI/MS/MS on a Nexera X2 UHPLC system (Shimadzu, Japan) coupled with a 6500+ QTRAP mass spectrometer (AB SCIEX). Chromatographic separation was performed on a XBridge amide column (150 mm × 4.6 mm, 3.5 µm; Waters Corp). Cholesterol esters and ceramides were measured in positive ion mode with fragments 369.4 and 264.4 respectively, triglycerides and diacylglycerides in positive ion mode with neutral loss fragments for the fatty acyl moiety, and phospholipids in negative ion mode by fatty acyl fragments. Lipid quantification was performed by scheduled multiple reaction monitoring, and peak integration was done with MultiQuant™ software (version 3.0.3). Signals were corrected for isotopic contributions (Python Molmass 2019.1.1) and quantified using internal standard signals, following the Lipidomics Standards Initiative guidelines. Lipidomics data was normalized to DNA concentration (nmol/mg) of the sample or to the total amount of lipid of its class (%). Zero values were replaced by 0.5 x the minimum values detected within the samples. For visualization relative percentages were used or log2 fold changes were generated using R 4.1.0 in RStudio 2023.12.1.402 (RStudio, USA) for beeswarm charts made in RAWGraph (<https://www.rawgraphs.io>) or heatmaps made in MetaboAnalyst online platform v6.0 (https://www.metaboanalyst.ca).

*Epilipidomics*

The same lipid extracts of the lipidomics experiment were used for epilipidomics analysis. Samples were dried under vacuum and resuspended in 100 µL isopropanol. Oxidized lipids were separated using an Accucore reverse phase C30 column (2.1 × 150 mm, 2.6 μm, 150 Å; Thermo Fisher Scientific) installed on a Vanquish Horizon UHPLC (Thermo Fisher Scientific) coupled online to Exploris 240 Hybrid Quadrupole Orbitrap mass spectrometers equipped with a H-ESI source (Thermo Fisher Scientific) Lipids were separated by gradient elution with solvent A (acetonitrile/water, 1:1, v/v) and B (isopropanol/acetonitrile/water, 85:10:5, v/v/v) both containing 5 mM NH_4_HCO_2_ and 0.1% (v/v) formic acid. Separation was performed at 50 °C with a flow rate of 0.3 ml/min using the following gradient: 0–20 min, 10-86% B; 20–24 min, 80-95% B; 24–27 min, 95-100%; 27–32min 100% (isocratic), 32.0-32.1 min 100-10%B; followed by 8 min column equilibration at 10% B. Mass spectra were acquired in negative ion mode with the following ESI source settings: spray voltage - static, negative ion spray voltage 2500 V, gas mode - static, sheath gas – 40 (arbitrary units), aux gas - 10 (arbitrary units), sweep gas –-1 (arbitrary units), ion transfer tube temperature − 300 °C, vaporizer temperature 370 °C, S-lens RF level – 35%. Optimization of oxidized complex lipid detection was performed using LPPtiger 2.0 as previously described(5,6)  with inclusion of the most abundant PE and PC species (Epilipidomics Inclusion List). For relative quantification, retention time scheduled parallel reaction monitoring using elemental composition of previously identified oxidized lipids as precursors was used in negative ion mode at the resolution of 17,500 at m/z 200, AGC target 1e5, maximum injection time of 200 ms. The isolation window for precursor selection was 1.5 m/z, number of microscans 2, and optimized collision energies (nCE 25). Data were acquired in profile mode. Acquired data was processed in Skyline 25.1(7)  considering fragment anions of oxidized fatty acyl chains as quantifiers. The obtained peak areas were normalized by appropriate lipid species from Ultimate SPLASH™ ONE internal standard mix (330820, Avanti Research). Zero values were replaced by 0.5 x the minimum values detected for a given oxidized lipid within the samples. For the epilipidomics heatmap, normalized peak area was further log-transformed and auto-scaled (mean-centered and divided by the standard deviation of each variable) in MetaboAnalyst online platform v6.0 (<https://www.metaboanalyst.ca/>).

*Transcriptomics*

Cells were grown on Petri dishes until 80–90% confluency, detached with a sterile scraper, washed with PBS, and pelleted by centrifugation. For RNA analysis, 1 × 10^6 cells were aliquoted, pelleted, and stored at −80 °C. Total RNA was extracted using the RNeasy® Micro Kit (74004, Qiagen), and poly(A)+ RNA was enriched with the NEBNext® Poly(A) mRNA Magnetic Isolation Module (E7490L, NEB). Strand-specific libraries were prepared with the NEBNext® Ultra™ II Directional RNA Library Prep Kit (E7760L, NEB) and sequenced on an Illumina platform. For RNA-seq analysis, reads were processed to obtain gene-level counts and analyzed in DESeq2 using R 4.1.0 in RStudio 2023.12.1.402 (RStudio, USA). Data were normalized with the median-of-ratios method and variance-stabilizing transformed for quality control and visualization. Differential expression between resistant (R) and sensitive (S) groups was tested with DESeq2, applying ASHR shrinkage for log2 fold changes and defining significance at false discovery rate (padj) ≤ 0.05 (Benjamini–Hochberg).

*Cy5-AA-LNP uptake validation*

To validate the cellular uptake of AA-LNP *in vitro*, SK-N-BE2C cells were seeded in 6-well plates and treated with Cy5-AA-LNP (no dialysis, diluted to reach 5µM AA per well). See ‘LNP formulation’ section for more info on the nanoparticle formulation. Next, cells were collected at 4-, 8-, 16-, 24- or 48-hour timepoints. Before collection, 2.5 nM SytoxGreen was added to each well for 30 min to stain death cells. Thereafter, cells were washed 3 times with 37°C DPBS and collected and analyzed by flow cytometry analysis (BD Accuri C6 Plus, BD Biosciences, USA). Live cell imaging detecting Cy5 signal, was performed using the Spark Cyto (Tecan, Switzerland).

# Supplementary Figures


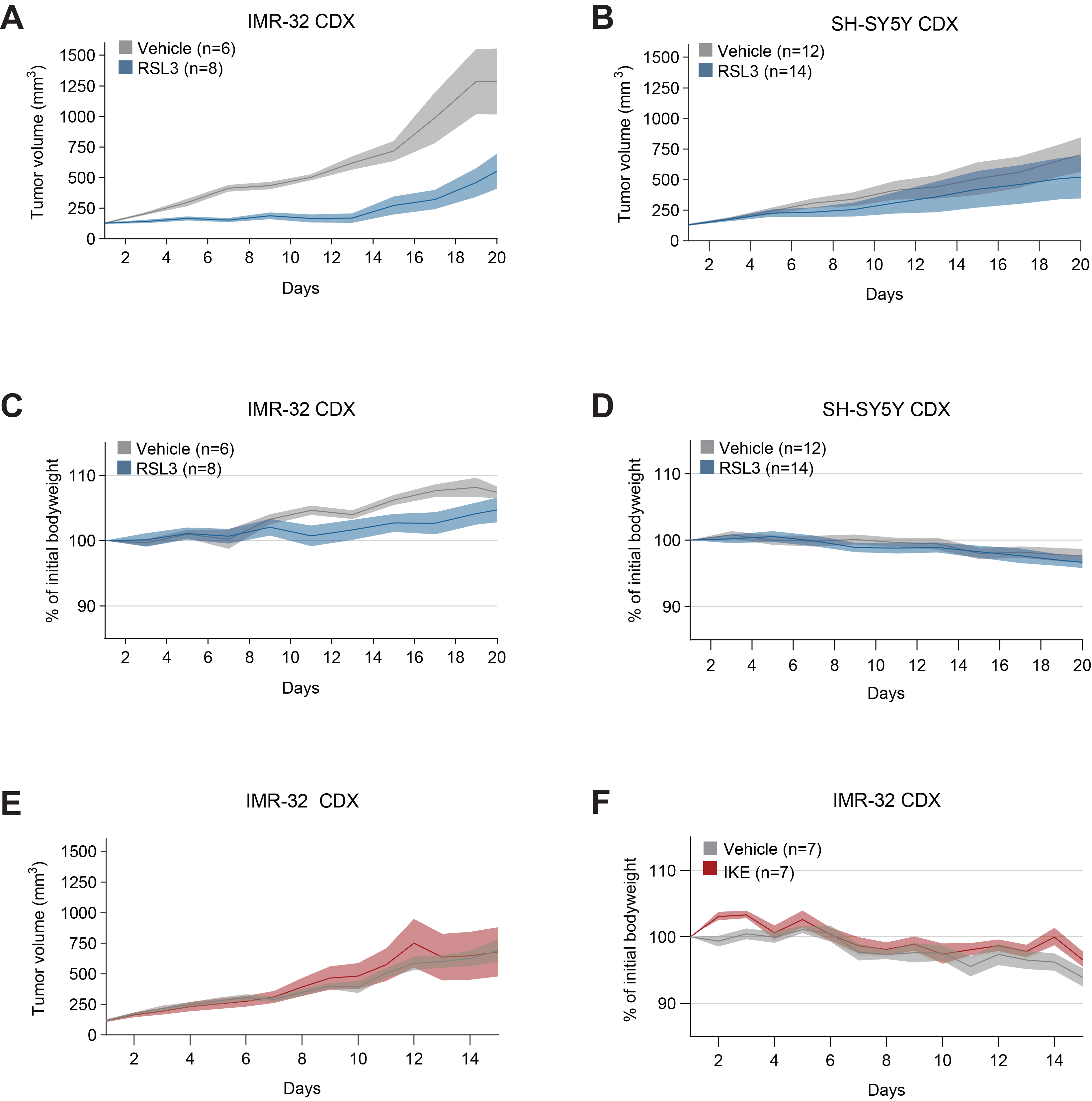


**Supplementary Fig. 1** **Heterogeneous ferroptosis responsiveness in CDX NB mouse models**. **A-D** Tumor volume (mm^3^) and percentage of initial bodyweight of IMR-32 (vehicle n = 6, RSL3 n = 8) and SH-SY5Y (vehicle n = 12, RSL3 n = 15) CDX mice receiving I.T. RSL3 injections every other day. **E,F** Tumor volume (mm^3^) and percentage of initial bodyweight of IMR-32 CDX mice receiving daily vehicle (n = 7) or IKE (n = 7) I.P. injections. CDX cell-derived xenograft.

**
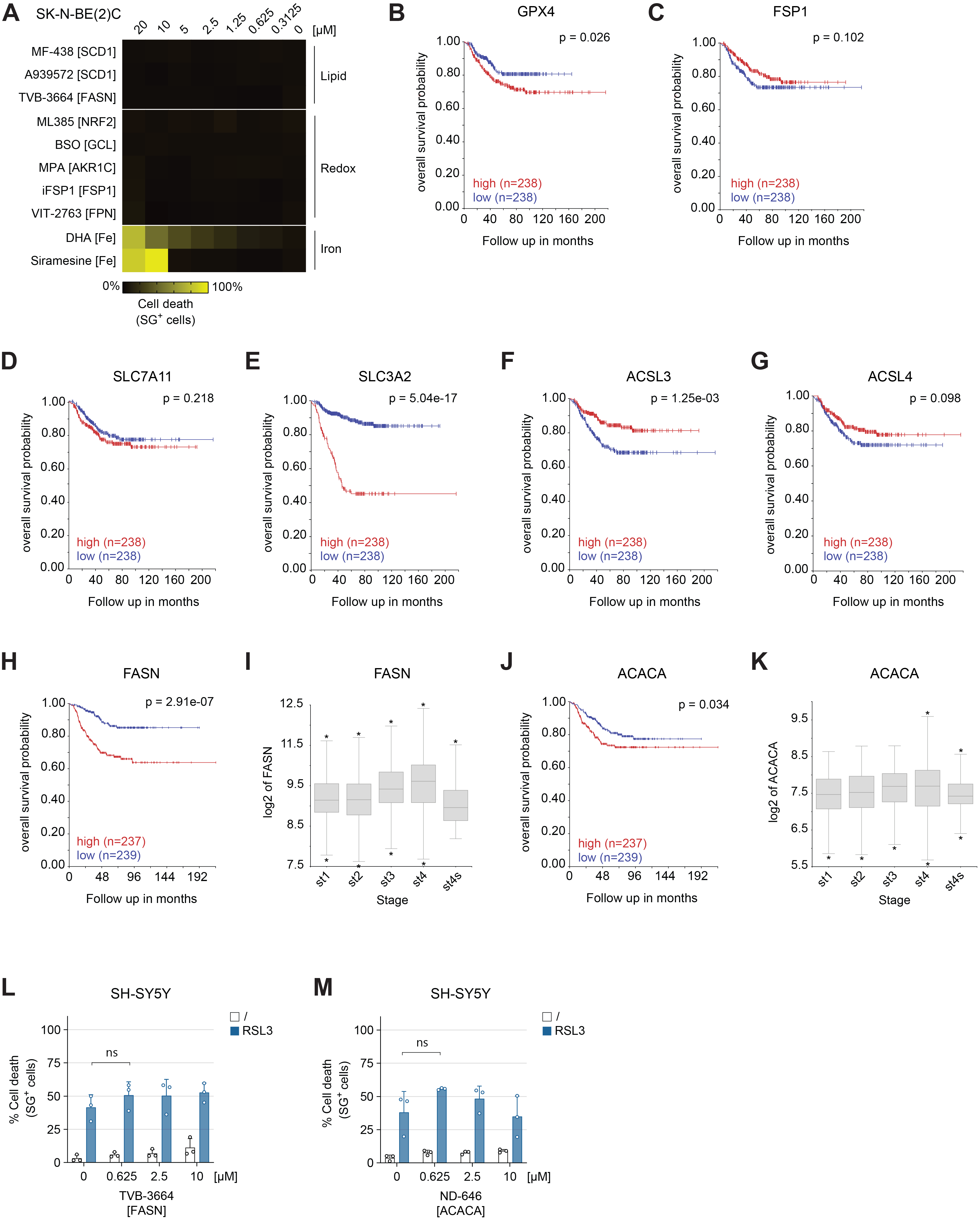
**

**Supplementary Fig. 2 Compound screen validation and selection. A** Heatmap showing mean cell death (%) of SK-N-BE(2)C cells treated 48h with 1µM corresponding compound (n = 2; Supplementary Table 4**). B-G** Kaplan–Meier survival curves of correlating the expression of key ferroptosis regulators with overall survival in the Kocak neuroblastoma patient cohort (n = 649; hgserver2.amc.nl) **H-K** Kaplan–Meier survival curves and boxplots showing that high FASN or ACACA expression correlate with poorer overall survival and advanced diagnostic stage in the Kocak neuroblastoma patient cohort (n = 649; hgserver2.amc.nl). **L, M** Percentage of cell death in SH-SY5Y cells pretreated 48h with TVB-3664 or ND-646, then 24h with RSL3 (5 µM) (n = 3). Data represented as mean ± SEM. Log-rank test (B-H,J). One-way ANOVA (I,K). Two-way Anova with Tukey’s multiple comparison (L,M). (*p ≤ 0.05).

**
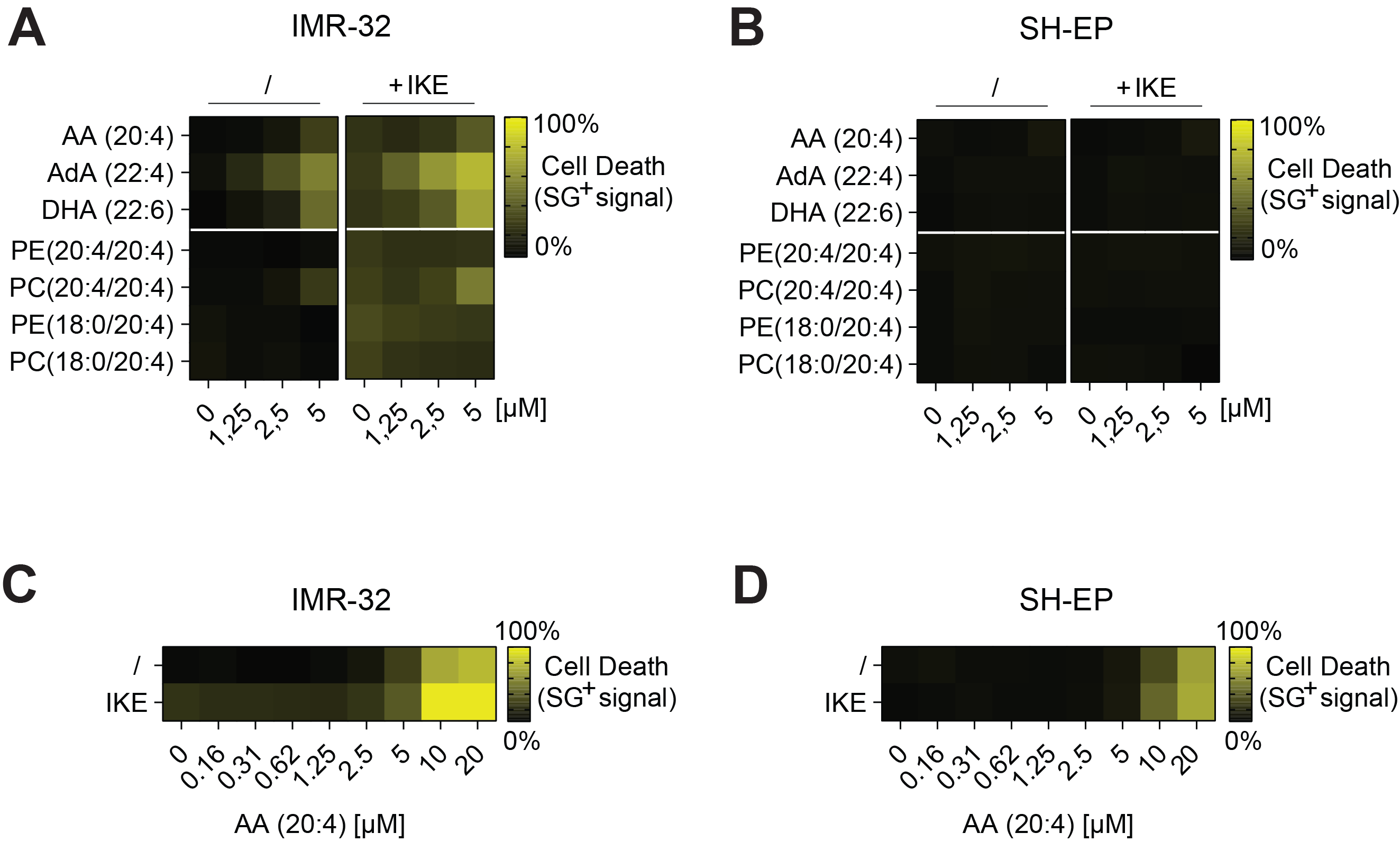
**

**Supplementary Fig. 3 Supplementation with PUFAs, but not PL-PUFAs, sensitizes high-risk NB cells. A-D** Heatmap showing mean cell death (%) in IMR-32 or SH-EP cells treated 72h with different PUFAs or PUFA-PLs, followed by IKE (10 µM in IMR-32, 0.5 µM in SH-EP) at 2% FBS media conditions (n = 3, Supplementary Table 6-9).

**
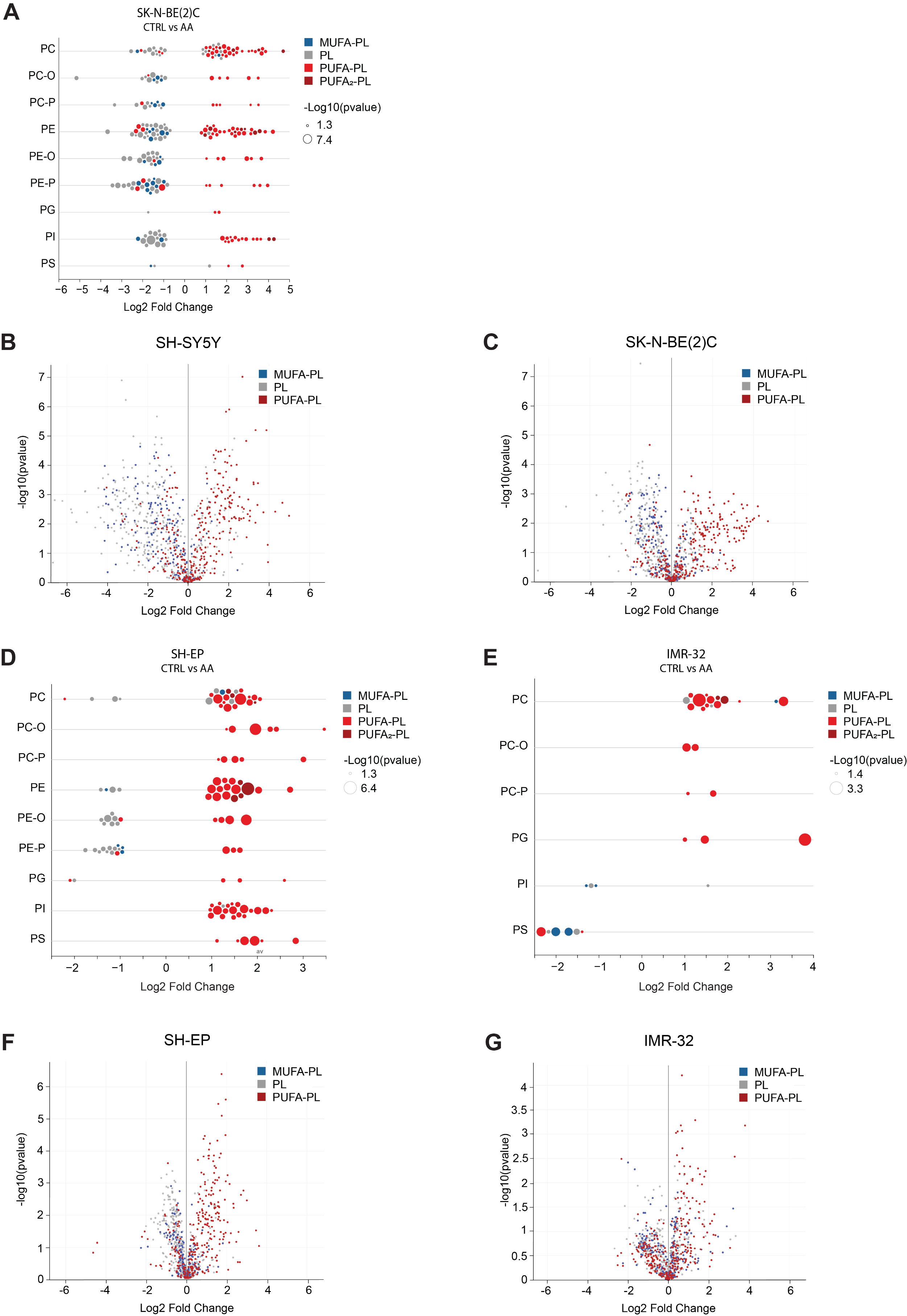
**

**Supplementary Fig. 4 AA supplementation boosts PL-unsaturation in SH-EP and SK-N-BE(2)C, but not IMR-32 NB cells. A-G** Log2 fold change of individual lipid species after 72h vehicle or AA treatment (1.25 µM in IMR-32, 5 µM in SH-EP, 5 µM in SK-N-BE(2)C, 20 µM in SH-SY5Y) at 2% FBS media conditions (n = 3). Two-tailed unpaired T-test.

**
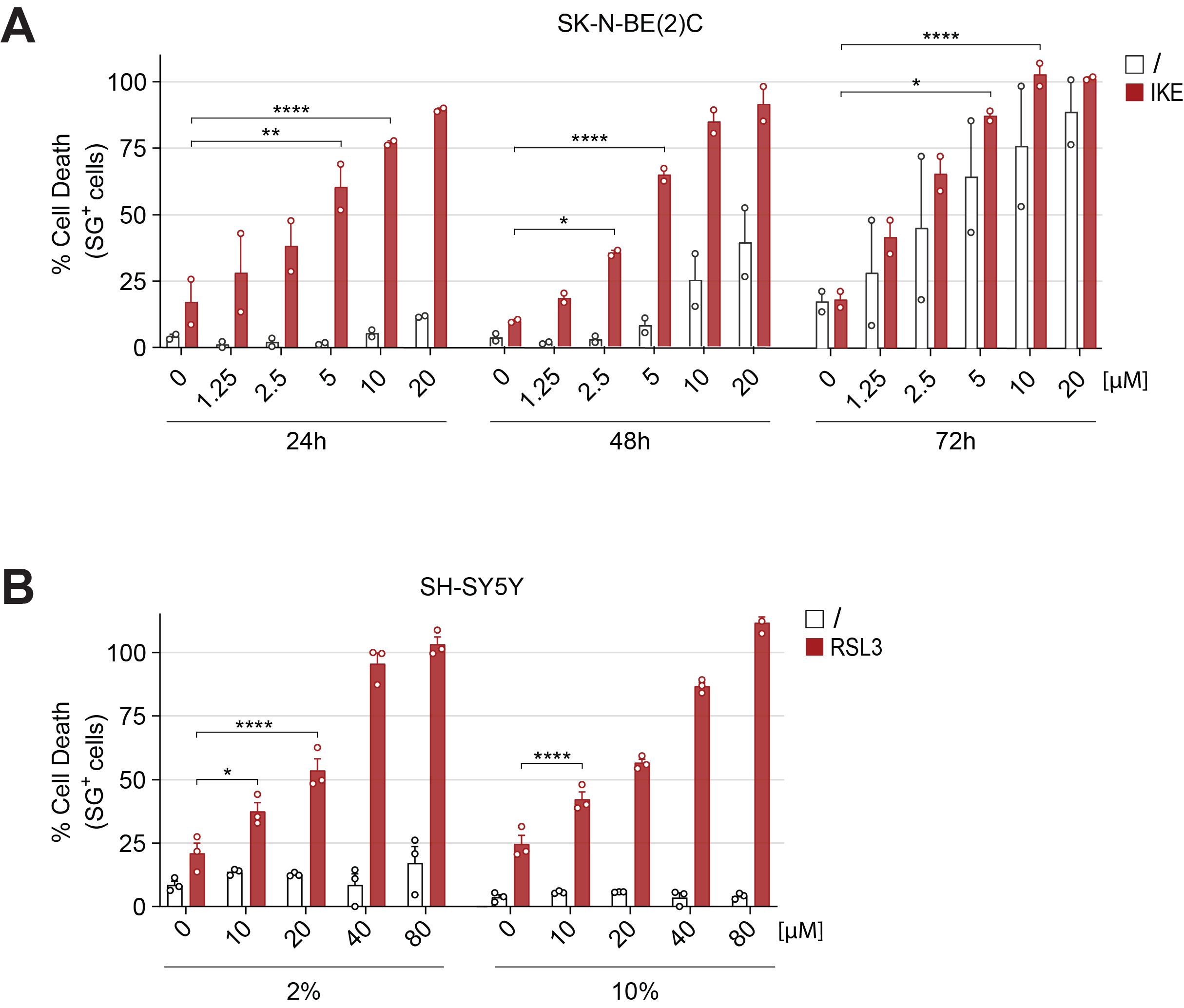
**

**Supplementary Fig. 5 AA supplementation remains a strong sensitizer after 24 hour pretreatment or 10% FBS culture conditions. A** Cell death (%) induced in SK-N-BE(2)C cells after 24h, 48h or 72h AA pretreatment, followed by 24h IKE (10µM, n = 2) exposure at 2% FBS media conditions. Data represented as mean ± SEM. **B** Cell death (%) induced in 2% vs 10% FBS media conditions in SH-SY5Y cells pretreated 48h with AA, followed by 24h RSL3 treatment (0.3 µM at 2% FBS, 2.5 µM at 10% FBS) (technical n = 3). Data represented as mean ± SD. Two-way Anova with Dunnett’s multiple comparison. (*p ≤ 0.05, **p ≤ 0.01, ***p ≤ 0.001, ****p ≤ 0.0001).

**
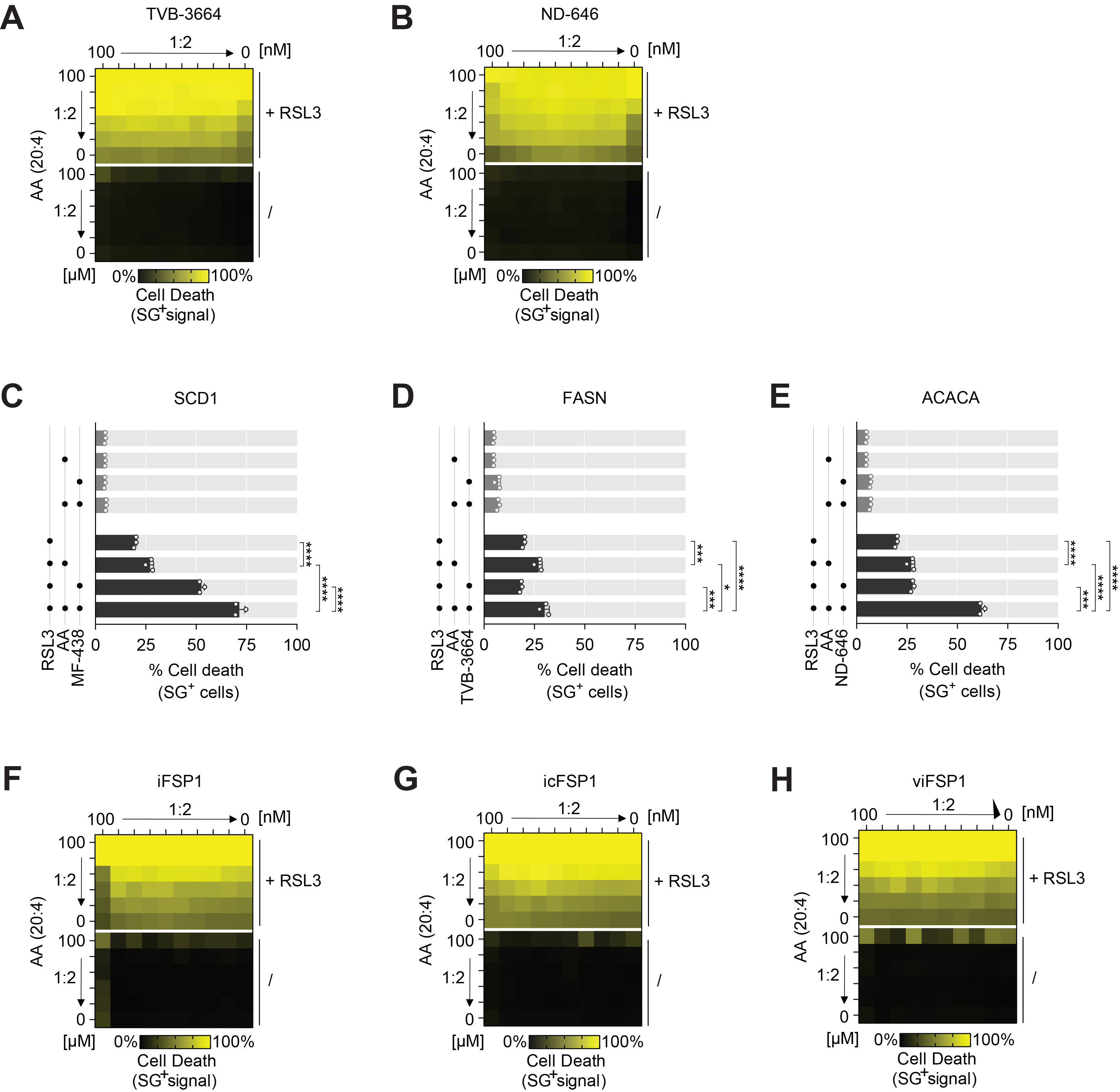
**

**Supplementary Fig. 6 *De novo* lipid synthesis or FSP1 inhibitors are less potent ferroptosis sensitizers compared to SCD1 inhibition in SH-SY5Y cells. A,B** Heatmap representing mean cell death (%) induced after combination pretreatment of AA and TVB-3664 or ND-646 at different concentrations in SH-SY5Y cells, followed by 24h RSL3 (5 µM, n = 3, Table S11 & S12) exposure. **C-E** Cell death percentage induced in SH-SY5Y cells after 48h combination pretreatment of AA (5µM) with compound (500 nM), followed by 24h RSL3 (0.5 µM, technical n = 3). Data represented as mean ± SD. **F-H** Heatmap showing mean cell death (%) after combination pretreatment of AA and iFSP1, icFSP1 or viFSP1 at different concentrations in SH-SY5Y cells, followed by 24h RSL3 (5 µM, technical n = 1, Supplementary Table 13-15) exposure. Two-way Anova with Tukey’s multiple comparison. (*p ≤ 0.05, **p ≤ 0.01, ***p ≤ 0.001, ****p ≤ 0.0001).

**
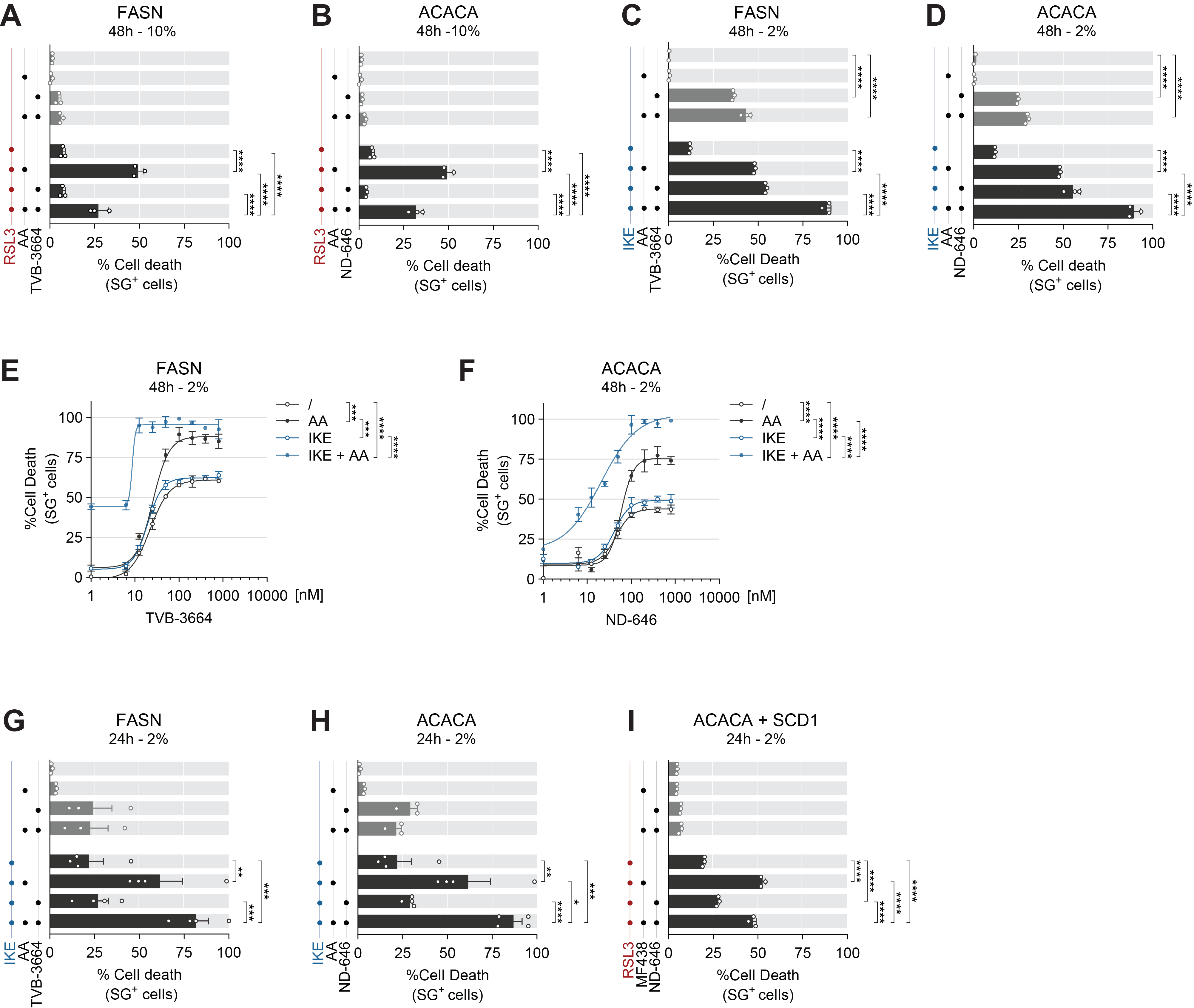
**

**Supplementary Fig. 7 2% FBS allows ferroptosis sensitization in SK-N-BE(2)C cells by *de novo* lipid synthesis inhibitors in presence of AA. A-D** Cell death (%) induced in SK-N-BE(2)C cells after 48h combination pretreatment of AA (2.5 µM) with compound (100nM), followed by 24h RSL3 (0.5 µM) at 10% FBS or 24h IKE (10 µM) at 2% FBS media conditions (technical n=3). Data represented as mean ± SD. **E,F** Cell death (%) induced in SK-N-BE(2)C cells after 48h pretreatment of compound ± AA (5 µM), followed by 24h IKE (10µM) exposure at 2% FBS media conditions (technical n =3 ). Data represented as mean ± SD. **G,H** Cell death (%) in SK-N-BE(2)C cells pretreated with AA (2,5µM) and/or compound (100 nM), followed by 24h IKE (10 µM) treatment in 2% FBS media (n = 4 for vehicle, n = 3 for IKE conditions). Data represented as mean ± SEM. **I** Cell death (%) in SK-N-BE(2)C cells pretreated with MF-438 (0.5 µM) and/or ND-646 (100 nM), followed by 24h RSL3 (50 nM) treatment (technical n = 1). Data represented as mean ± SD. Two-way Anova with Tukey’s multiple comparison. (*p ≤ 0.05, **p ≤ 0.01, ***p ≤ 0.001, ****p ≤ 0.0001).

**
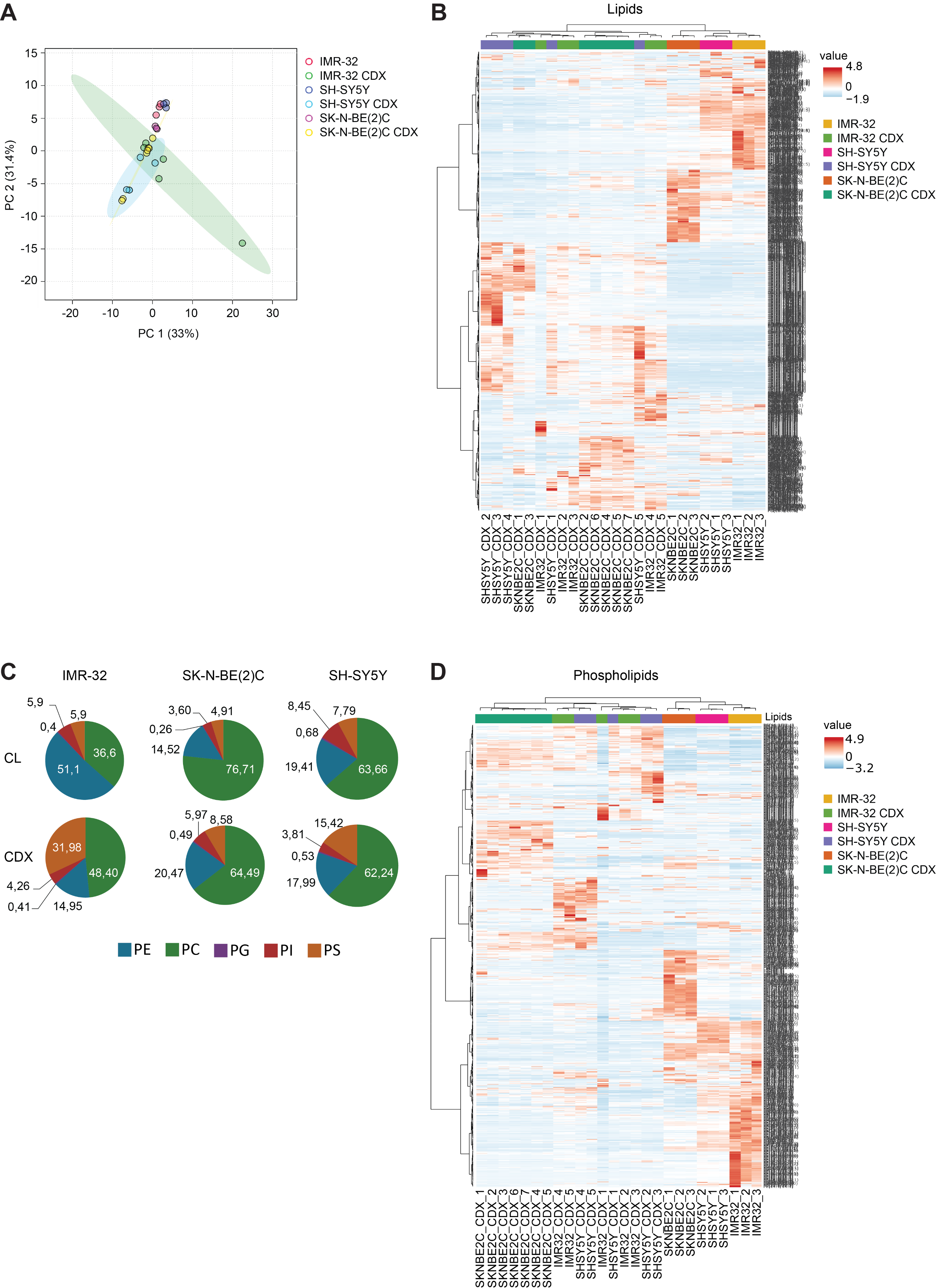
**

**Supplementary Fig. 8 Exploration of the lipidome and phospholipidome in high-risk NB cell lines and corresponding CDX models. A** Principal-component analysis of full lipidome (% of total lipids) in untreated high-risk NB cell lines (CL) and matched CDX tumors (n = 3 for cell lines, n = 5 for IMR-32 CDX, n = 7 for SK-N-BE(2)C CDX, n = 5 for SH-SY5Y CDX). **B** Hierarchical-clustering heatmap of all lipid species (% of total lipids). Data were row-scaled (z-scores), clustering by Euclidean distance with Ward’s linkage. **C-D** Phospholipid class distribution and hierarchical-clustering heatmap of phospholipids (normalized as % of total phospholipids).

**
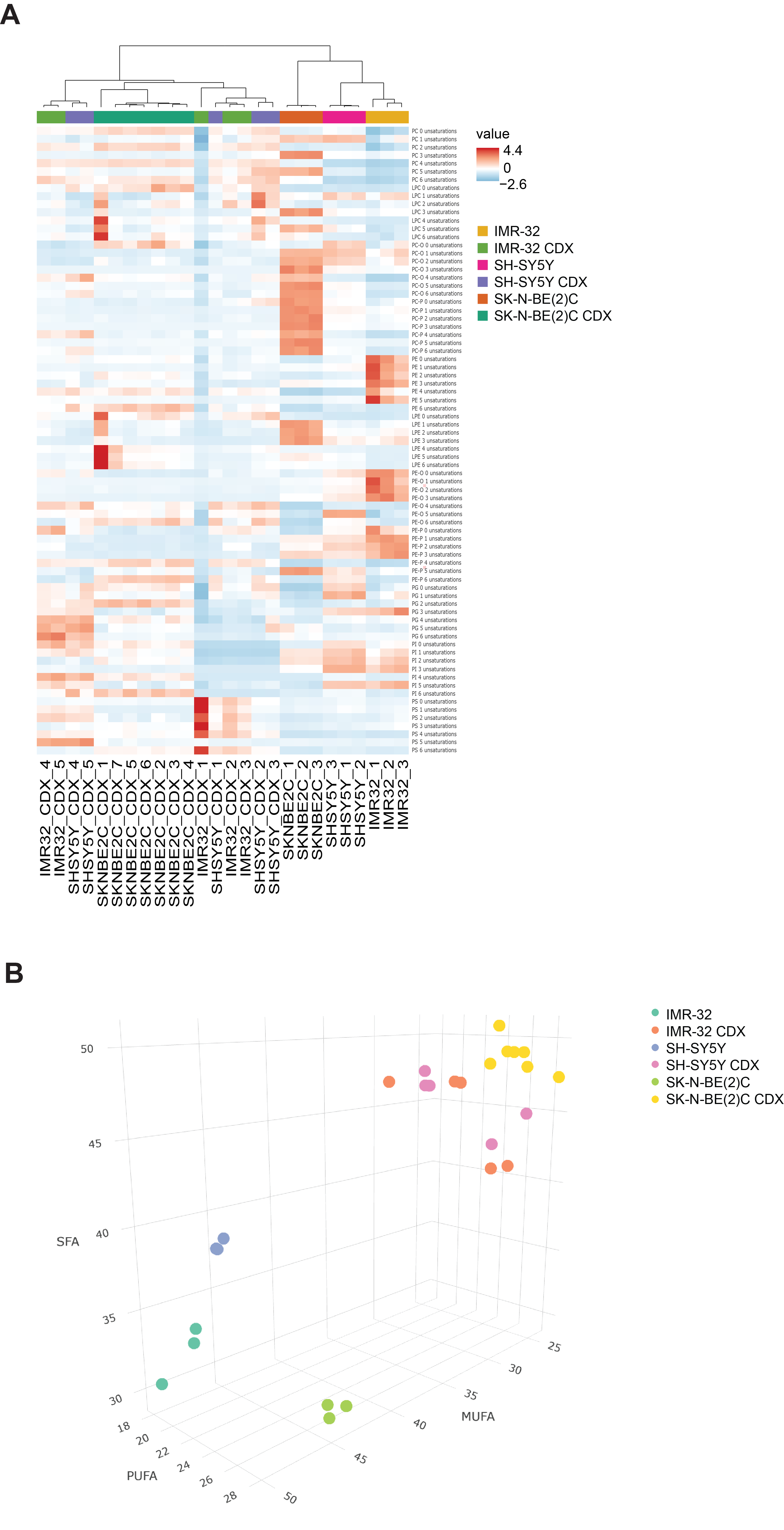
**

**Supplementary Fig. 9 Phospholipidome unsaturation comparison in high-risk NB cell lines and matched CDX tumors. A** Hierarchical-clustering (on samples only) heatmap of phospholipid unsaturation data (normalized for % of phospholipids) in untreated high-risk NB cell lines and matched CDX tumors (n = 3 for cell lines, n = 5 for IMR-32 CDX, n = 7 for SK-N-BE(2)C CDX, n = 5 for SH-SY5Y CDX). Data were row-scaled (z-scores), clustering by Euclidean distance with Ward’s linkage. **B** 3D-plot clustering high-risk NB cell lines and CDX tumors based on SFA, MUFA and PUFA percentages (data is % of phospholipids).

**
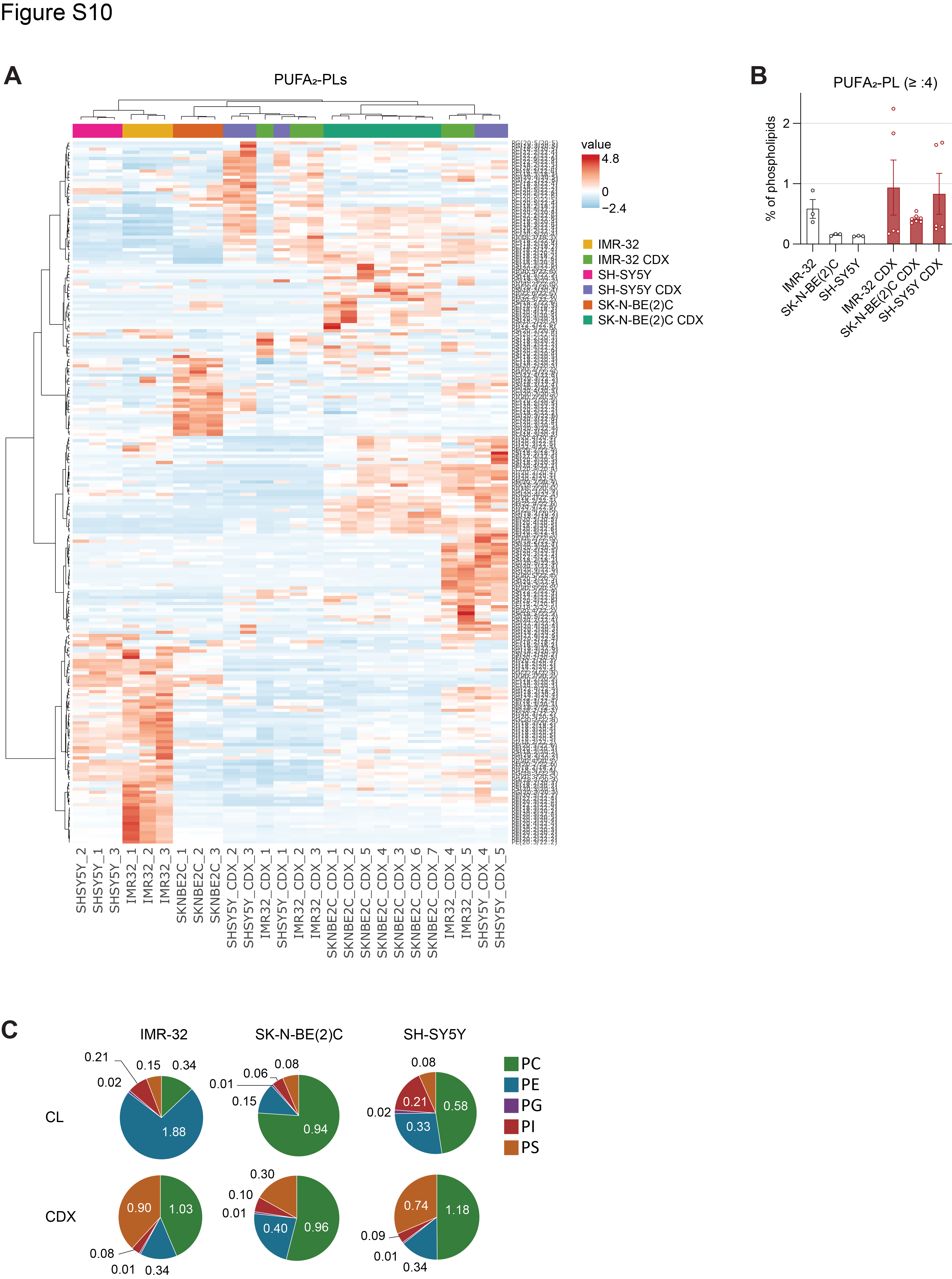
**

**Supplementary Fig. 10 PUFA_2_-PL analysis in high-risk NB cell lines and corresponding CDX models. A** Hierarchical-clustering heatmap of all PUFA_2_-PLs species (% of phospholipids) in untreated high-risk NB cell lines and matched CDX tumors (n = 3 for cell lines, n = 5 for IMR-32 CDX, n = 7 for SK-N-BE(2)C CDX, n = 5 for SH-SY5Y CDX). Data were row-scaled (z-scores), clustering by Euclidean distance with Ward’s linkage. **B** PUFA_2_-PLs with unsaturation ≥ 4 (% of phospholipids) in high-risk NB cell lines and matched CDX tumors. Data represented as mean ± SEM. Two-way Anova with Tukey’s multiple comparison. **C** Pie charts showing the phospholipid class distribution of PUFA_2_-PLs in high-risk NB cell lines (CL) and CDX tumors. Data represented as % of phospholipids.

**
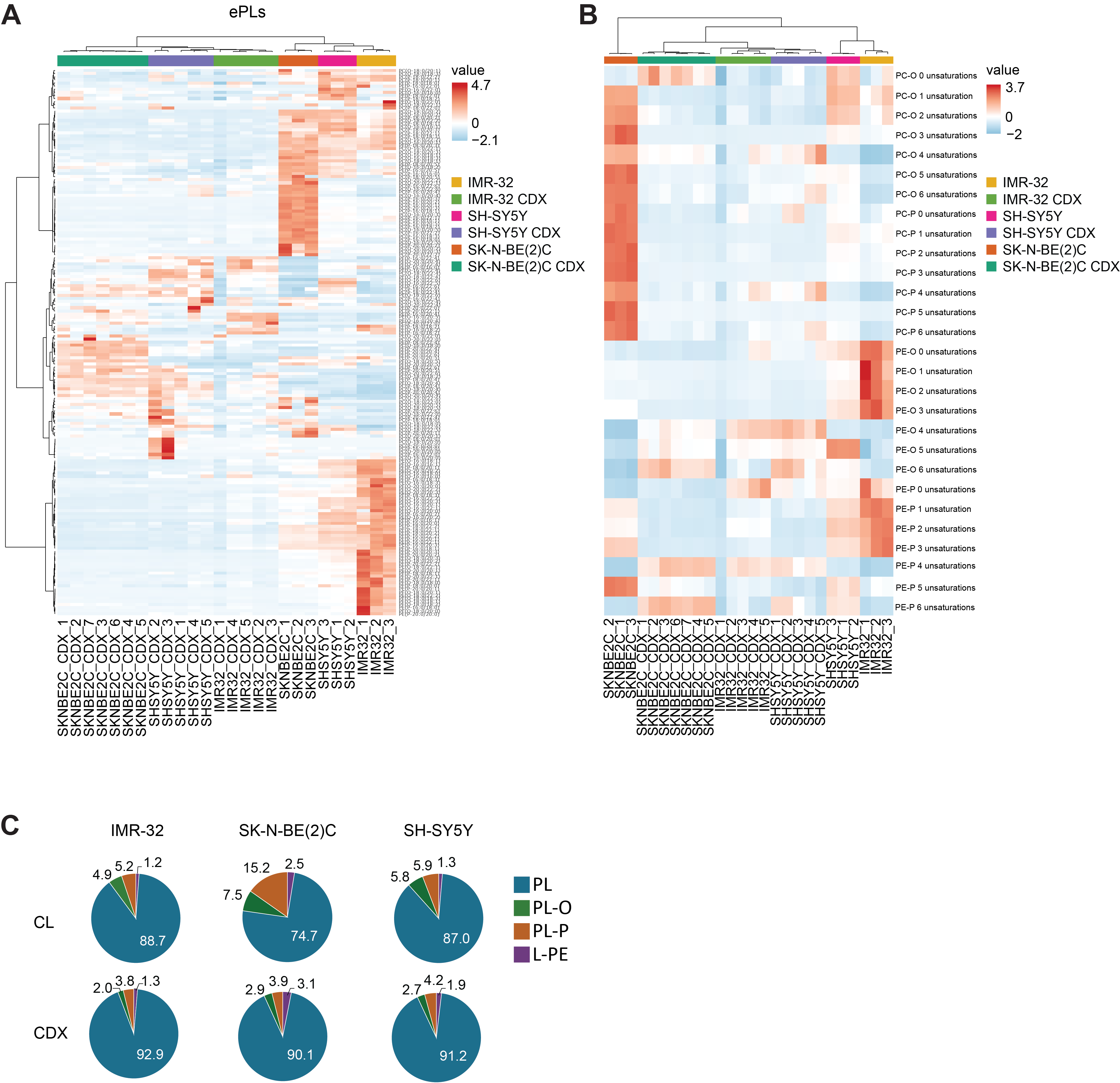
**

**Supplementary Fig. 11 Ether-linked PLs analysis in high-risk NB cell lines and matched CDX models. A** Hierarchical-clustering heatmap of all ether-linked PLs species (% of phospholipids) in untreated high-risk NB cell lines and matched CDX tumors (n = 3 for cell lines, n = 5 for IMR-32 CDX, n = 7 for SK-N-BE(2)C CDX, n = 5 for SH-SY5Y CDX). Data were row-scaled (z-scores), clustering by Euclidean distance with Ward’s linkage. **B** Hierarchical-clustering (on samples only) heatmap of all ether-linked PLs unsaturation levels (% of phospholipids). Data were row-scaled (z-scores), clustering by Euclidean distance with Ward’s linkage. **C** Pie charts showing ether-linked PL (PL-O and plasmalogens PL-P) distribution (% of phospholipids). PC-O 1-alkyl,2-acylphosphatidylcholine, PC-P 1-alkenyl,2-acylphosphatidylcholine, PE-O 1-alkyl,2-acylphosphatidylethanolamines, PE-P 1-alkenyl,2-acylphosphatidylethanolamines, CL cell lines, PL phospholipids, PL-O alkyl-ether-phospholipids, PL-P alkenyl-ether-phospholipids, L-PL lyso-phospholipids.

**
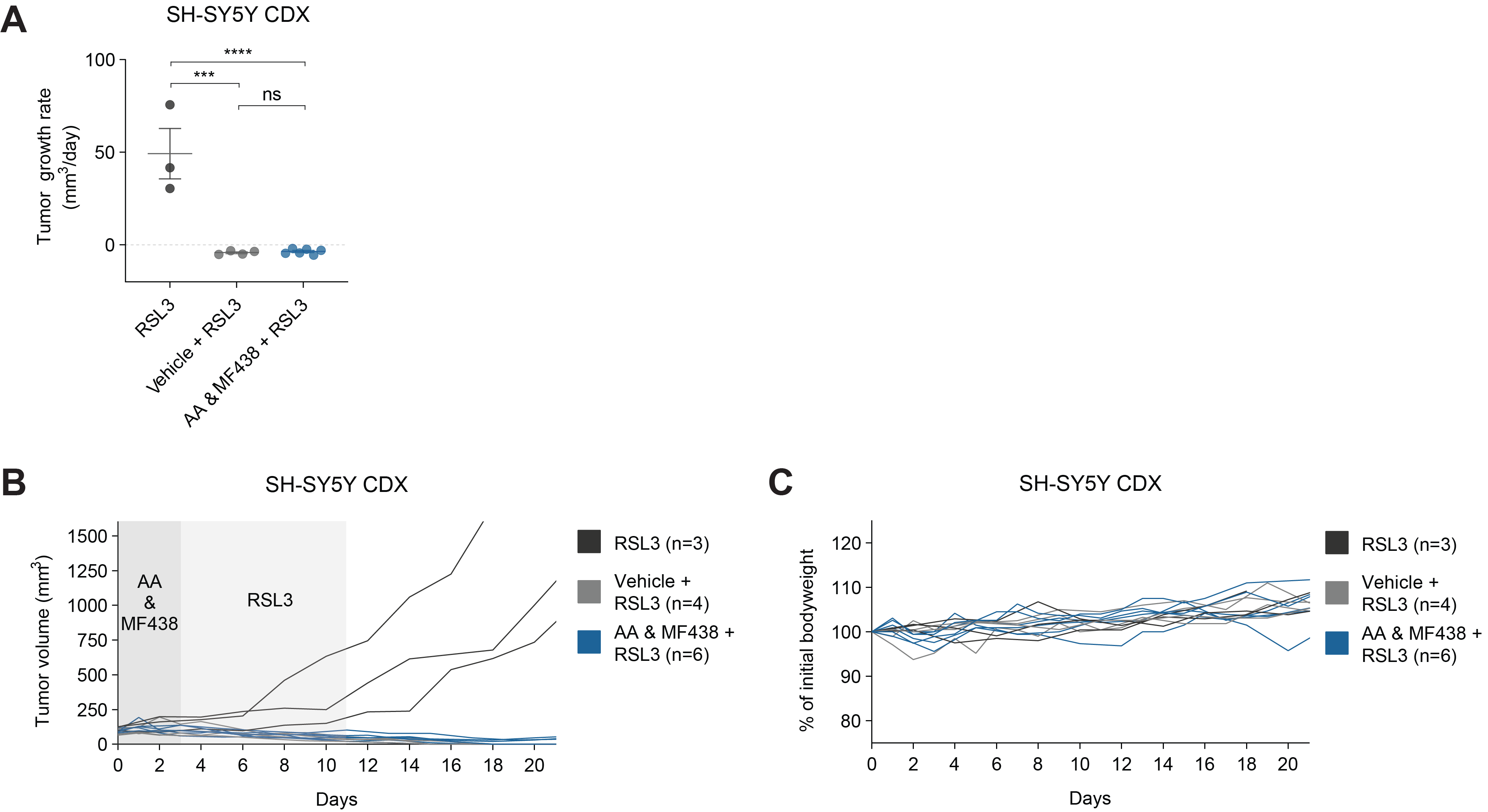
**

**Supplementary Fig. 12 Vehicle toxicity effect of AA and MF-438 solution *in vivo*. A-C** Tumor growth rate (mm3/day), tumor volume and % of initial bodyweight over time in SH-SY5Y CDX mice receiving no treatment (n = 3), 3 daily I.T. vehicle injections (n = 4, 10% DMSO, 40% PEG300, 2% Tween80, 48% Saline solution) or 5 mM AA and 1 mM MF-438 in vehicle (n = 6), followed in all groups by 4 alternate-day I.T. injections of RLS3. Tumor growth rate is presented as mean ± SEM. One-way Anova. (***p ≤ 0,001, ****p ≤ 0,0001)

**
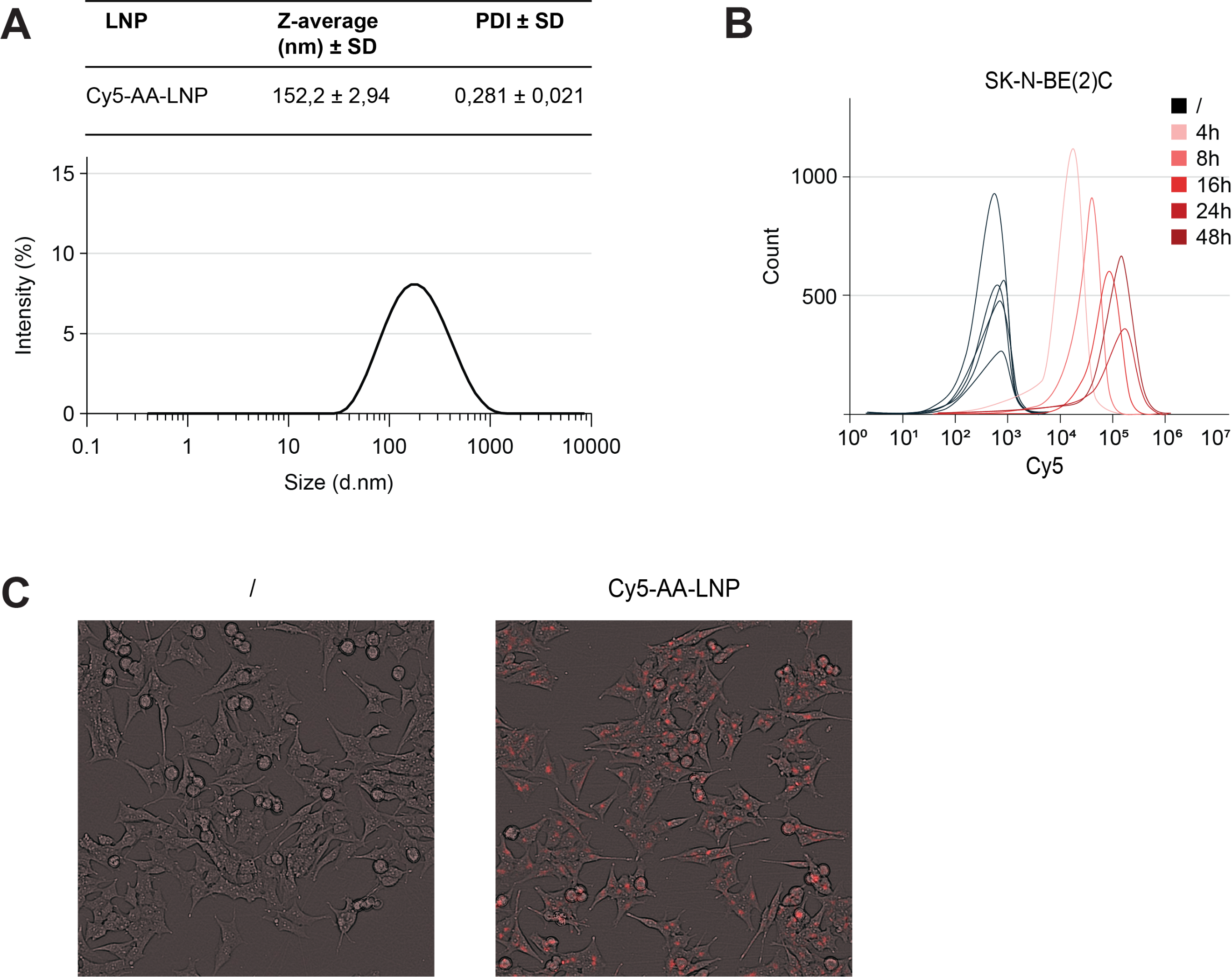
**

**Supplementary Fig. 13 Cy7 labelled analogue validates AA-LNP uptake. A** Biophysical properties and intensity-based size distribution curves of Cy5-AA-LNP formulation (technical n = 3). **B** Flow cytometric analysis of Cy5 signal on live-gated cells (SytoxGreen-negative cells) after 4h, 8h, 16h, 24h or 48h treatment of SK-N-BE(2)C cells with Cy5-AA-LNP (5 µM AA) (technical n = 1). **C** Fluorescence image of SK-N-BE(2)C cells after 24h treatment with or without Cy5-AA-LNP (5 µM AA).

**
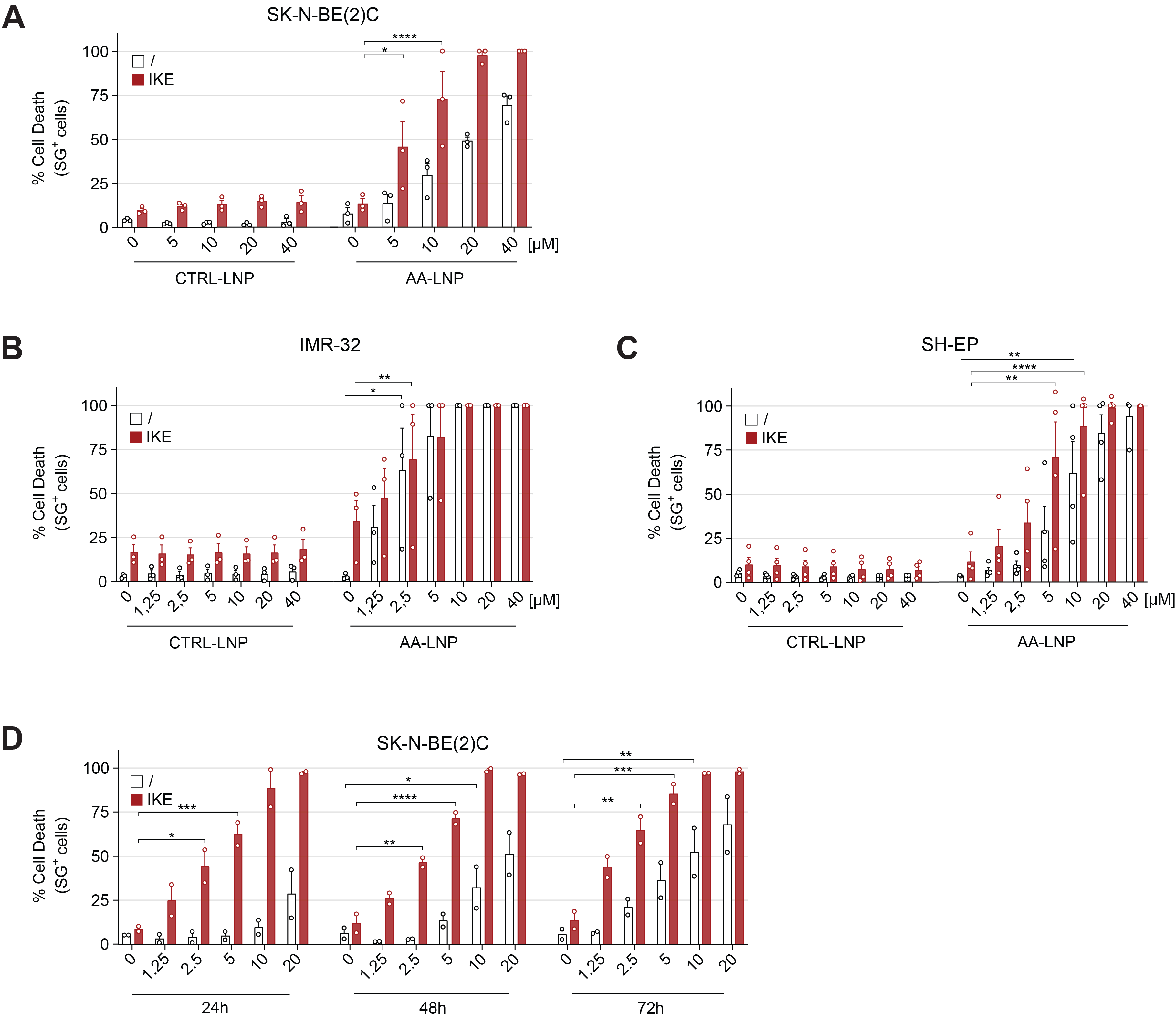
**

**Supplementary Fig. 14 Sensitizing effect of AA-LNP in other high-risk NB cells. A-C** Cell death (%) induced in SK-N-BE(2)C, IMR-32 or SH-EP cells pretreated with CTRL-LNP or AA-LNP for 72h, followed by 24h IKE (10 µM in SK-N-BE(2)C, 5 µM in IMR-32, 1 µM in SH-EP) treatment at 2% FBS media conditions (n = 3). **D** Cell death (%) induced in SK-N-BE(2)C cells after 24h, 48h or 72h AA-LNP pretreatment, followed by 24h IKE (10 µM) exposure at 2% FBS media conditions (n = 2). Data represented as mean ± SEM. Two-way Anova with Dunnett’s multiple comparison. (*p ≤ 0,05, **p ≤ 0,01, ***p ≤ 0,001, ****p ≤ 0,0001).

**
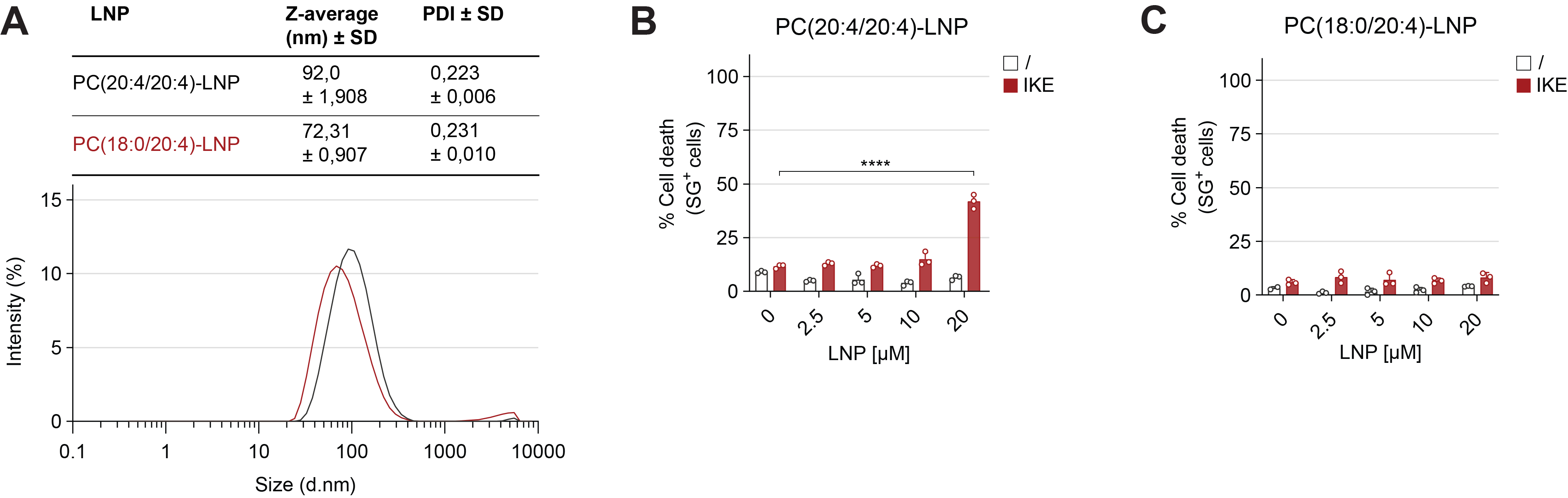
**

**Supplementary Fig. 15 PUFA-PL containing LNPs do not enhance ferroptosis induction. A** Summarizing table of the biophysical properties and intensity-based size distribution curves of PC(20:4/20:4)-LNP and PC(18:0/20:4)-LNP formulations (technical n = 3). **B,C** Cell death (%) induced in SK-N-BE(2)C cells after 72h LNP pretreatment, followed by 24h IKE (10 µM) exposure at 2% FBS media conditions (technical n = 3). Data represented as mean ± SD. Two-way Anova with Dunnett’s multiple comparison. (****p ≤ 0,0001).

**
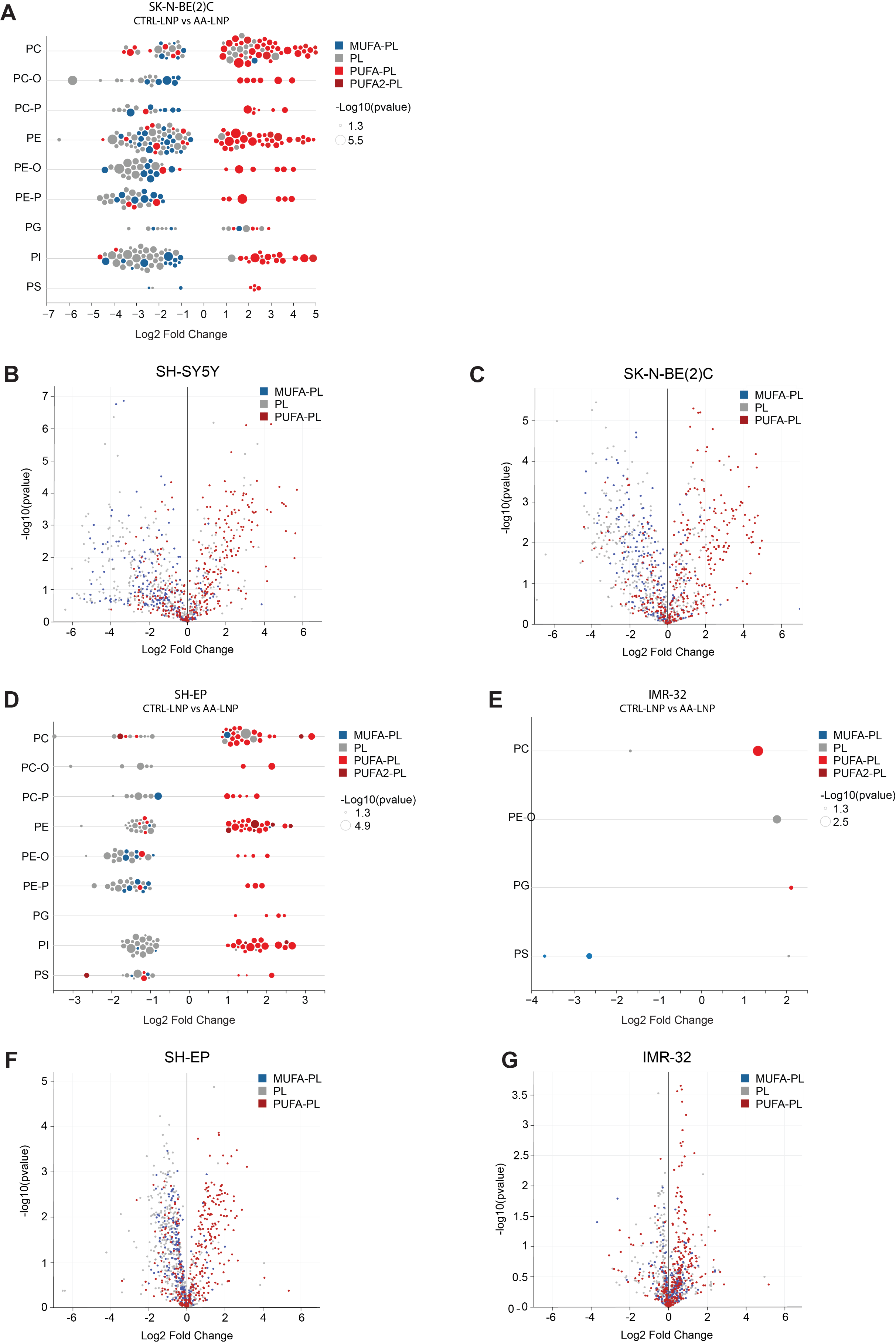
**

**Supplementary Fig. 16 AA-LNP enhances PUFA-enrichment in SH-EP and SKNBE2C, not in IMR-32 cells. A-G** Log2 fold change of individual lipid species comparing 72h CTRL-LNP versus AA-LNP treatment (10 µM AA in SK-N-BE(2)C, 20 µM AA in SH-SY5Y, 1.25 µM AA in IMR-32, 5 µM AA in SH-EP) at 2% FBS media conditions (n = 3). Two-tailed unpaired T-test.

**
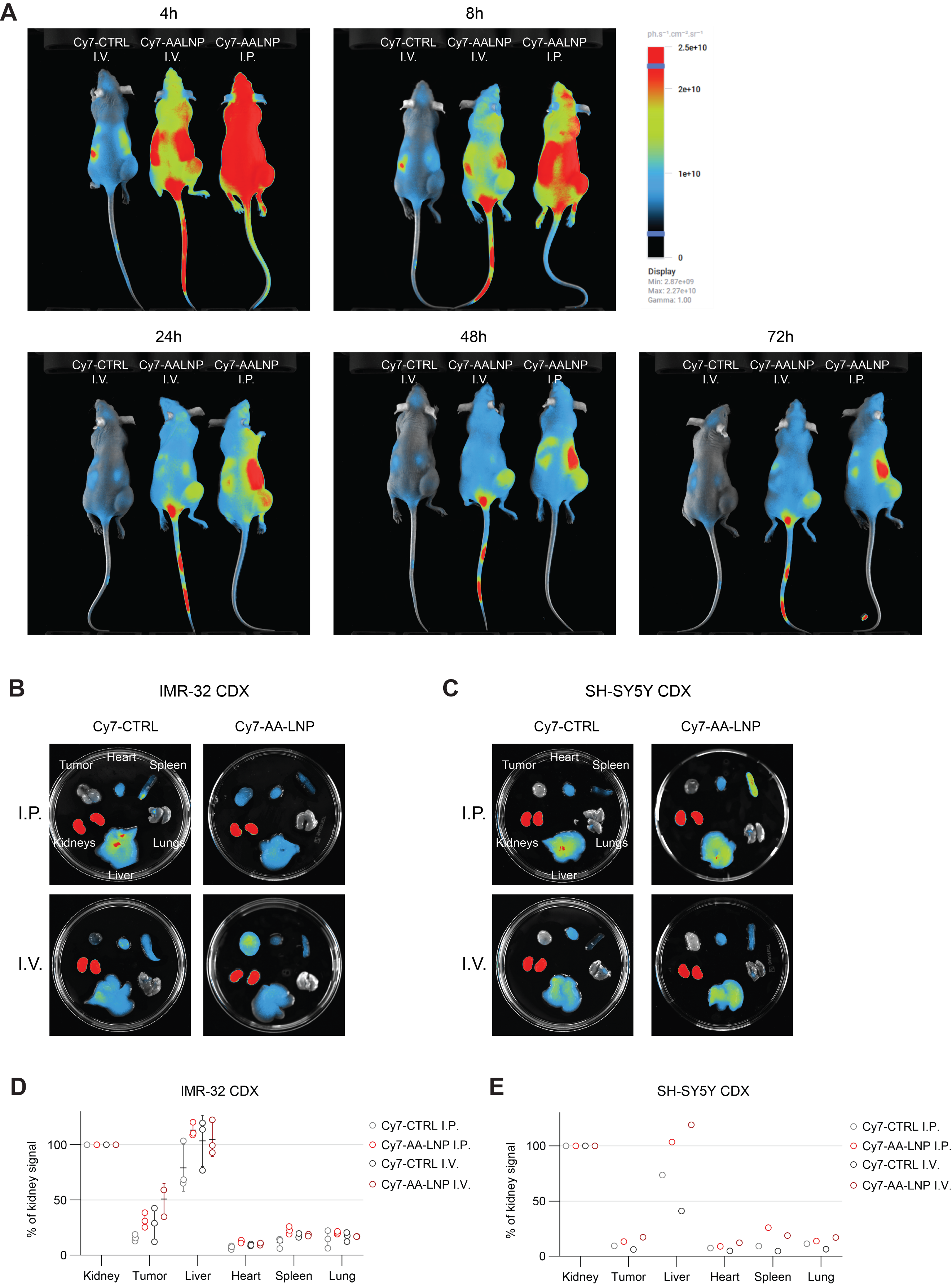
**

**Supplementary Fig. 17 AA-LNP demonstrates tumor targeting properties in IMR-32, not in SH-SY5Y CDX mice. A** *In vivo* fluorescence images of IMR-32 CDX mice 4h, 8h, 24h, 48h and 72h post-injection with non-encapsulated 18:1 Cy7-PE (I.V., Cy7-CTRL) or Cy7-AA-LNP (I.P. or I.V.). **B-E** *Ex vivo* fluorescence images and quantification of organ signal (normalized to kidney signal) in IMR-32 (n = 3) and SH-SY5Y (n = 1) CDX mice. Data represented as mean ± SEM for IMR-32 CDX data.

**
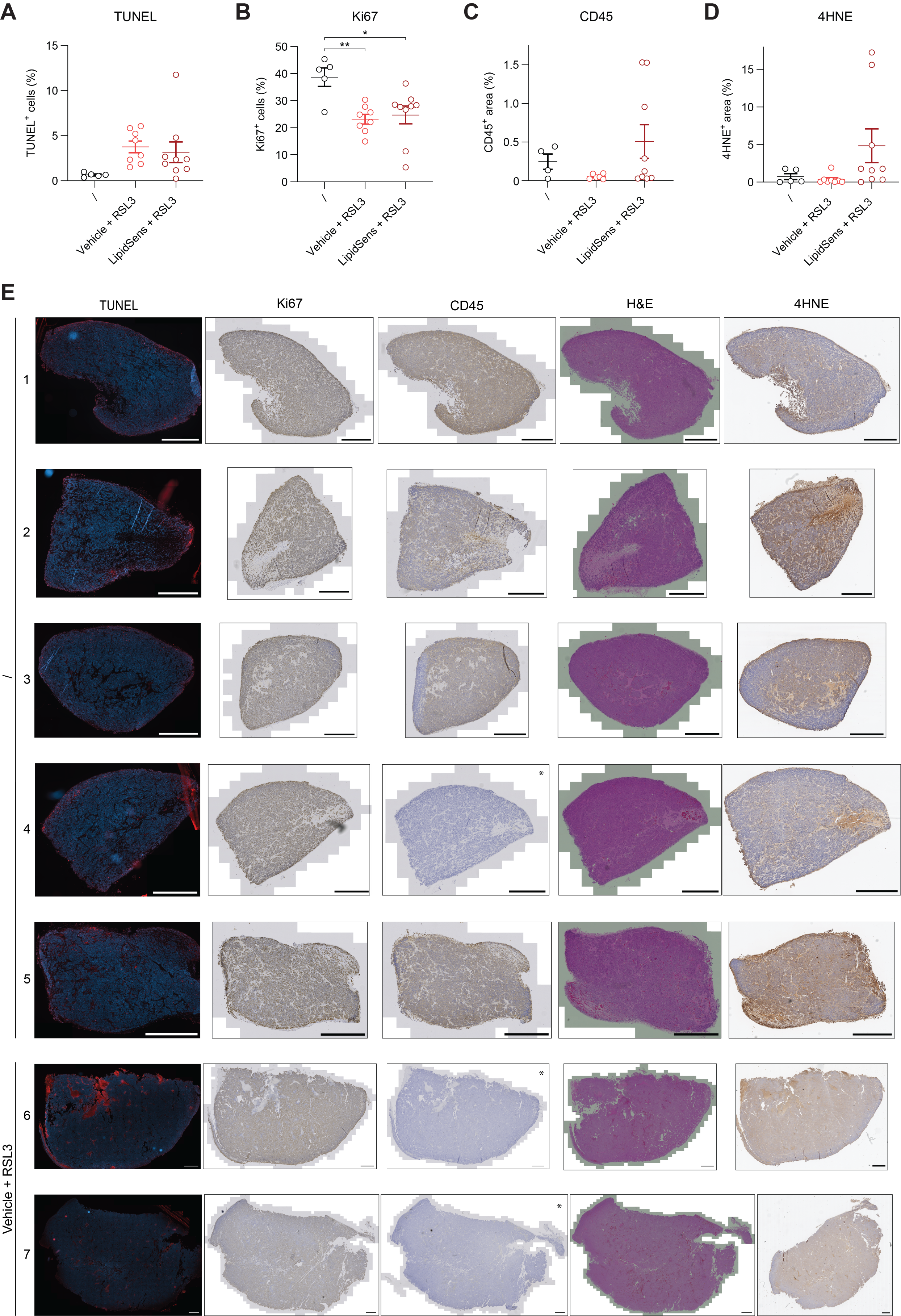
**

**
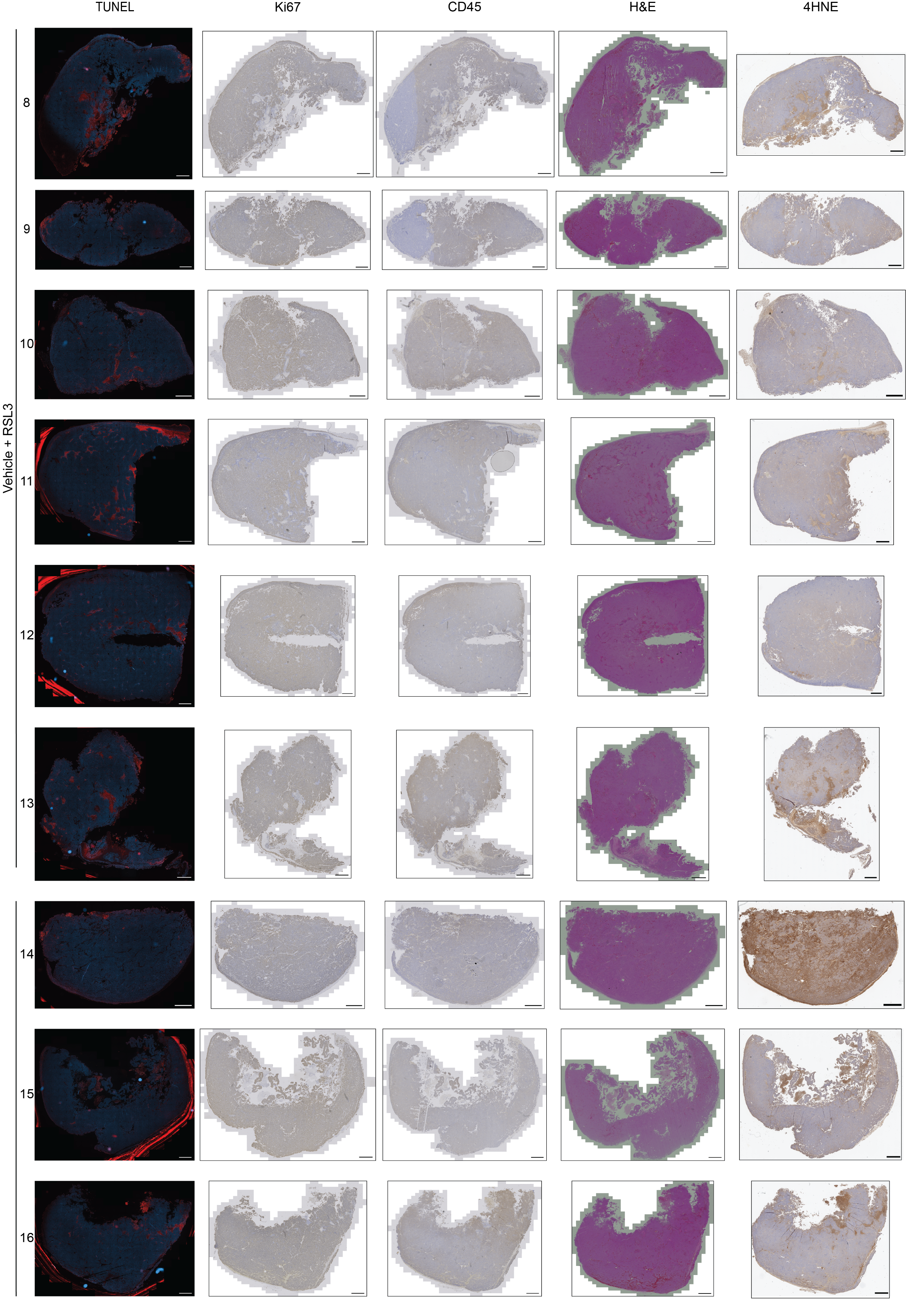
**

**
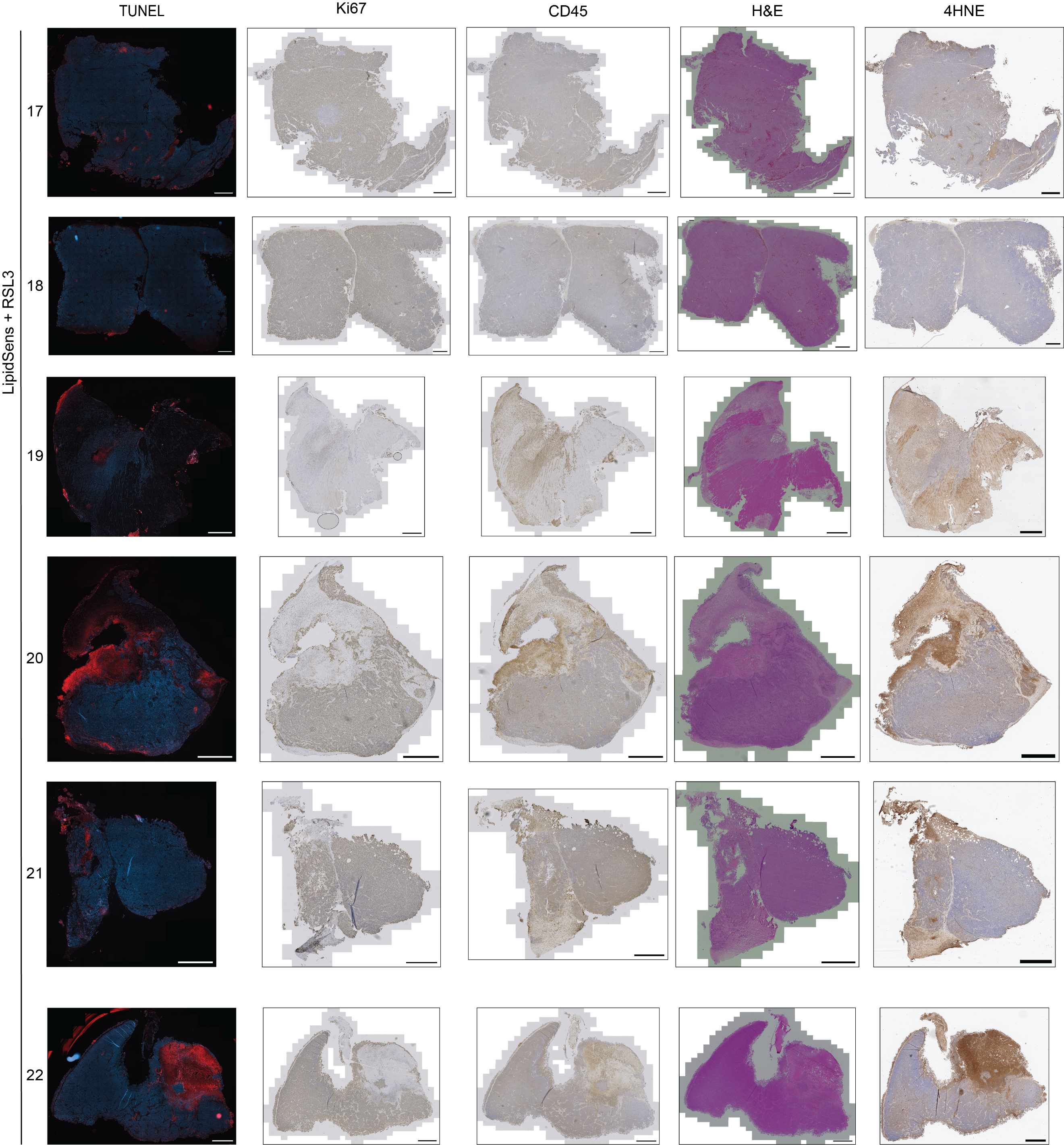
Supplementary Fig. 18 TUNEL, Ki67 and CD45 staining in treated IMR-32 CDX tumors. A-D** Quantification of TUNEL-positive cells and Ki67-positive cells (each expressed relative to hematoxylin-stained nuclei), CD45-positive area (relative to tissue area) and 4HNE-positive area (relative to tissue area) in whole-slide images of IMR-32 CDX tumors: untreated (n = 5), vehicle + RSL3 (n = 8) or LipidSens + RSL3 (n = 9). **E** Representative whole-slide images of IMR-32 CDX tumors showing TUNEL staining for DNA fragmentation, Ki67 immunohistochemistry for proliferation, CD45 immunohistochemistry for immune-cell infiltration, H&E staining and 4HNE immunohistochemistry for lipid peroxidation product deposits. Scale bar 1mm. Data represented as mean ± SEM. One-way ANOVA with Tukey’s multiple comparison. (*p ≤ 0.05, **p ≤ 0.01)

**
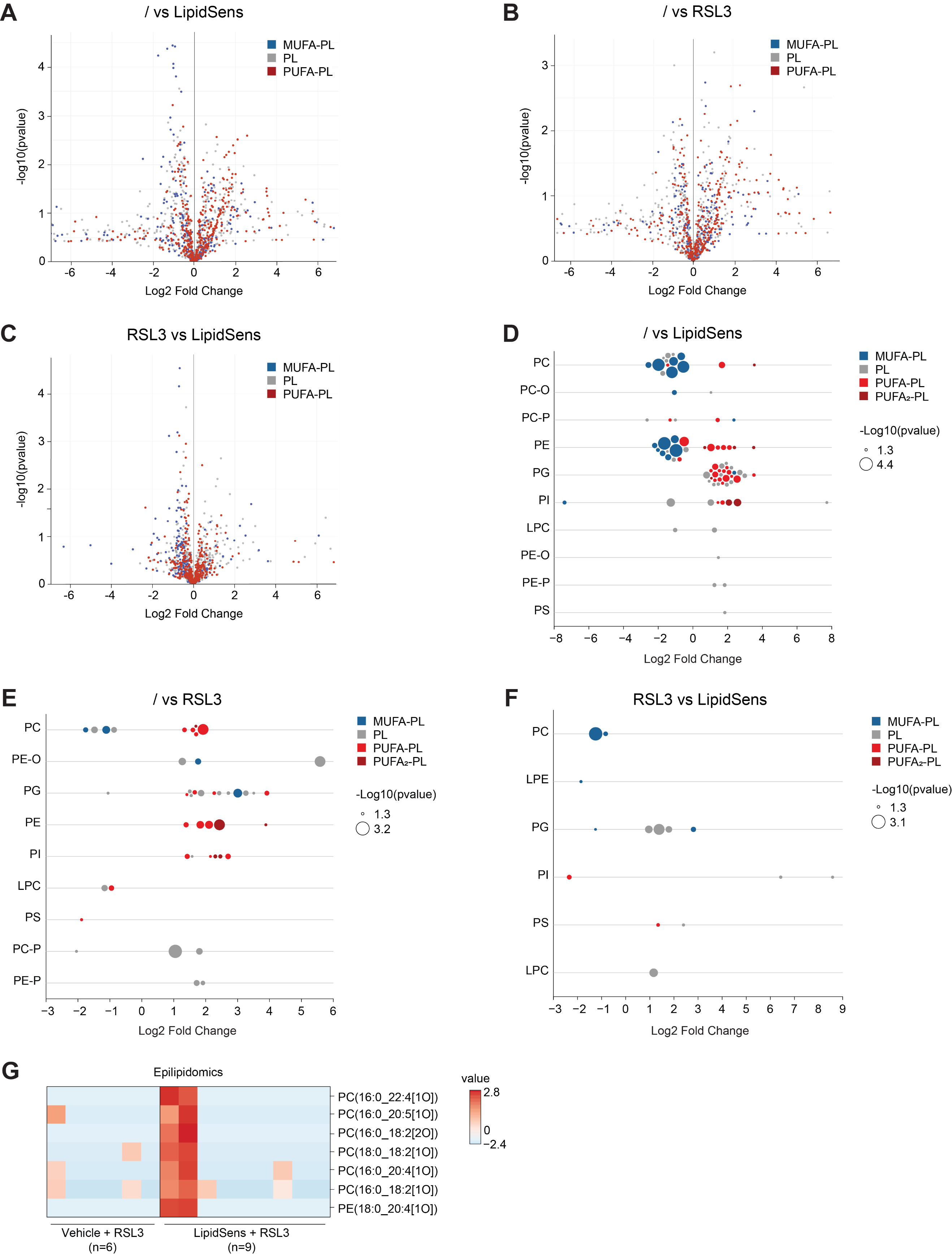
Supplementary Fig. 19 RLS3 increases lipid unsaturation in IMR-32 CDX tumors, enhanced further by co-treatment with AA-LNP and SCD1 inhibition. A-F** Log2 fold change of individual lipid species comparing untreated (n = 5), vehicle + RSL3 (n = 8) or LipidSens + RSL3 (n = 9) treated IMR-32 CDX tumors. Two-tailed unpaired T-test. **G** Heatmap representing normalized peak areas (log-transferred, auto-scaled) of significantly differentiated oxidized PE or PC lipid species. MUFA mono-unsaturated fatty acid, PC phosphatidylcholine, PC-O 1-alkyl,2-acylphosphatidylcholine, PC-P 1-alkenyl,2-acylphosphatidylcholine, PE phosphatidylethanolamine, PE-O 1-alkyl,2-acylphosphatidylethanolamine, PE-P 1-alkenyl,2-acylphosphatidylethanolamine, PG phosphatidylglycerol, PI phosphatidylinositol, PL phospholipid, PUFA poly-unsaturated fatty acid, PUFA_2_-PL phospholipid containing two poly-unsaturated fatty acids, PS phosphatidylserine.

**
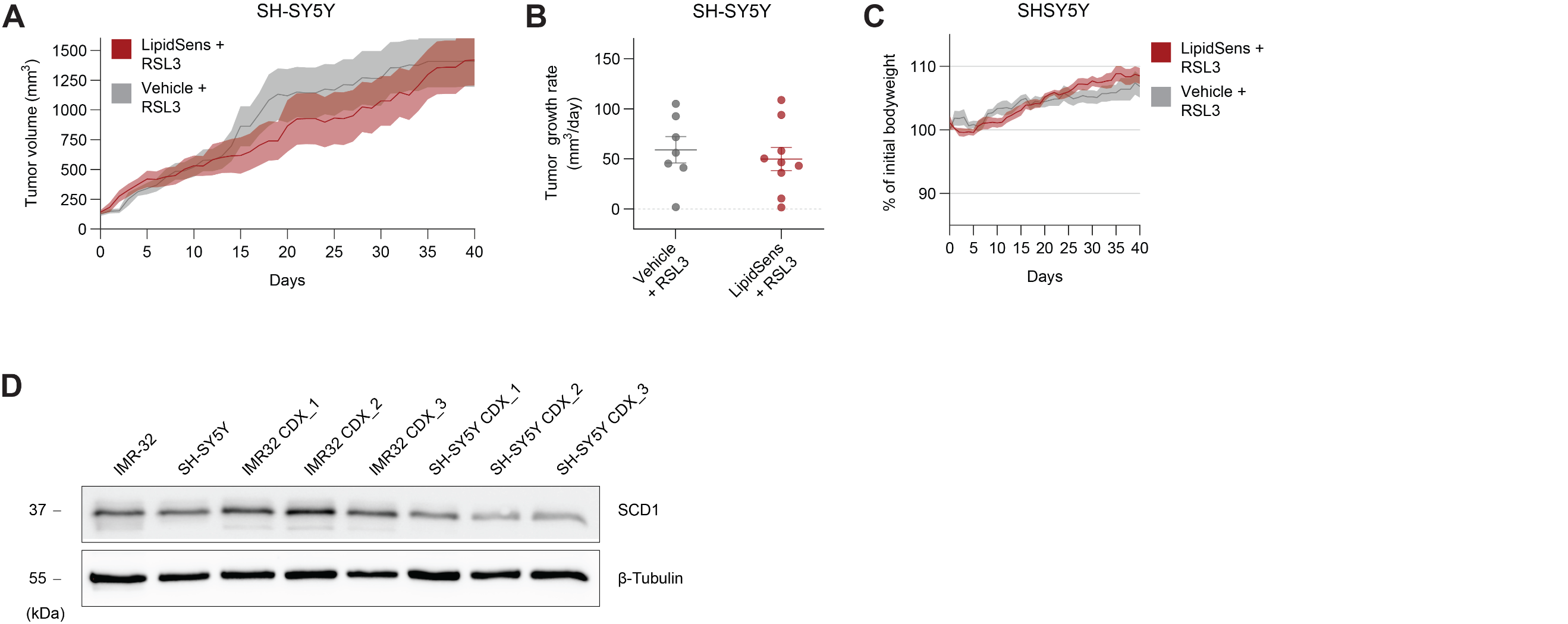
**

**Supplementary Fig. 20 No enhancing effect of AA-LNP and SCD1 targeting treatment in resistant SH-SY5Y CDX model. A-C** Tumor volume (mm^3^), tumor growth rate (mm^3^/day) and % of initial bodyweight of SH-SY5Y CDX mice treated with I.T. vehicle (n = 7, 10%DMSO in DPBS) or LipidSens (n = 9, Cy7-AA-LNP in DPBS containing 13.2 mM AA + 0.5 mM MF-438 reaching 10% DMSO) alternated daily with I.T. RSL3 injections. **D** Western blot detecting SCD1 protein levels in untreated IMR-32 and SH-SY5Y cell lines and matching CDX tumors. Data represented as mean ± SEM. Two-tailed unpaired T-test. (B).

# Supplementary Tables

**Supplementary Table 1: Cell death (%) in high-risk NB cell lines after 24h exposure to different ferroptosis inducers.**

|  | IMR-32 | | | IMR-32 | | | IMR-32 | | | IMR-32 | | |
| --- | --- | --- | --- | --- | --- | --- | --- | --- | --- | --- | --- | --- |
|  | RSL3 | | | ML162 | | | IKE | | | FINO2 | | |
| [nM] | %CD | SEM | N | %CD | SEM | N | %CD | SEM | N | %CD | SEM | N |
| 20000 | 98.07 | 1.32 | 2.00 | 90.85 | 4.09 | 2.00 | 91.87 | 2.41 | 2.00 | 93.75 | 2.23 | 3.00 |
| 10000 | 94.46 | 1.59 | 2.00 | 90.42 | 3.91 | 2.00 | 93.85 | 1.51 | 2.00 | 95.65 | 1.07 | 3.00 |
| 5000 | 92.03 | 3.54 | 2.00 | 94.08 | 1.70 | 2.00 | 92.37 | 2.51 | 2.00 | 86.33 | 2.84 | 3.00 |
| 2500 | 87.27 | 1.88 | 5.00 | 90.81 | 1.50 | 5.00 | 94.93 | 1.65 | 5.00 | 56.01 | 5.46 | 3.00 |
| 1250 | 89.49 | 1.09 | 5.00 | 88.81 | 2.57 | 5.00 | 94.65 | 2.38 | 5.00 | 24.94 | 4.40 | 3.00 |
| 625 | 90.00 | 0.85 | 5.00 | 90.49 | 1.37 | 5.00 | 90.38 | 2.06 | 5.00 | 7.92 | 1.82 | 3.00 |
| 312 | 91.86 | 1.39 | 5.00 | 92.40 | 1.83 | 5.00 | 56.44 | 7.11 | 5.00 | 4.82 | 1.78 | 3.00 |
| 156 | 93.59 | 1.71 | 5.00 | 91.25 | 2.33 | 5.00 | 16.37 | 8.85 | 5.00 | 1.63 | 0.53 | 3.00 |
| 78 | 93.77 | 2.39 | 5.00 | 90.14 | 7.04 | 5.00 | 7.15 | 4.17 | 5.00 | 0.84 | 0.33 | 3.00 |
| 0 | 1.35 | 0.56 | 5.00 | 1.02 | 0.16 | 5.00 | 1.35 | 0.56 | 5.00 | 0.60 | 0.27 | 3.00 |

**Supplementary Table 1 cont.**

|  | SH-EP | | | SH-EP | | | SH-EP | | | SH-EP | | |
| --- | --- | --- | --- | --- | --- | --- | --- | --- | --- | --- | --- | --- |
|  | RSL3 | | | ML162 | | | IKE | | | FINO2 | | |
| [nM] | %CD | SEM | N | %CD | SEM | N | %CD | SEM | N | %CD | SEM | N |
| 20000 | 100.00 | 0.00 | 4.00 | 100.00 | 0.00 | 4.00 | 100.00 | 0.00 | 4.00 | 87.34 | 12.66 | 4.00 |
| 10000 | 100.00 | 0.00 | 4.00 | 100.00 | 0.00 | 4.00 | 100.00 | 0.00 | 4.00 | 66.34 | 19.84 | 4.00 |
| 5000 | 100.00 | 0.00 | 4.00 | 100.00 | 0.00 | 4.00 | 99.32 | 0.68 | 4.00 | 45.48 | 20.50 | 4.00 |
| 2500 | 97.28 | 2.72 | 4.00 | 100.00 | 0.00 | 4.00 | 76.22 | 23.78 | 4.00 | 22.43 | 13.91 | 4.00 |
| 1250 | 90.87 | 8.79 | 4.00 | 98.80 | 1.20 | 4.00 | 34.23 | 22.70 | 4.00 | 5.52 | 3.55 | 4.00 |
| 625 | 73.55 | 15.43 | 4.00 | 83.38 | 9.22 | 4.00 | 5.67 | 5.34 | 4.00 | 0.74 | 0.43 | 4.00 |
| 312 | 54.88 | 20.83 | 4.00 | 52.23 | 16.77 | 4.00 | 0.52 | 0.07 | 4.00 | 0.45 | 0.11 | 4.00 |
| 156 | 44.03 | 19.76 | 4.00 | 41.82 | 16.26 | 4.00 | 0.61 | 0.10 | 4.00 | 0.63 | 0.13 | 4.00 |
| 78 | 38.61 | 18.53 | 4.00 | 34.42 | 18.30 | 4.00 | 0.75 | 0.11 | 4.00 | 0.83 | 0.04 | 4.00 |
| 0 | 0.67 | 0.24 | 4.00 | 0.67 | 0.24 | 4.00 | 0.77 | 0.14 | 4.00 | 0.81 | 0.05 | 4.00 |

**Supplementary Table 1 cont.**

|  | SK-N-BE(2)C | | | SK-N-BE(2)C | | | SK-N-BE(2)C | | | SK-N-BE(2)C | | |
| --- | --- | --- | --- | --- | --- | --- | --- | --- | --- | --- | --- | --- |
|  | RSL3 | | | ML162 | | | IKE | | | FINO2 | | |
| [nM] | %CD | SEM | N | %CD | SEM | N | %CD | SEM | N | %CD | SEM | N |
| 20000 | 98.67 | 0.54 | 3.00 | 93.85 | 2.25 | 3.00 | 56.20 | 12.23 | 3.00 | 94.56 | 2.03 | 3.00 |
| 10000 | 70.86 | 7.79 | 3.00 | 90.20 | 1.65 | 3.00 | 50.10 | 18.83 | 3.00 | 92.23 | 3.40 | 3.00 |
| 5000 | 59.72 | 8.76 | 3.00 | 84.70 | 4.53 | 3.00 | 33.49 | 15.98 | 3.00 | 71.18 | 8.62 | 3.00 |
| 2500 | 53.35 | 7.11 | 3.00 | 67.98 | 6.37 | 3.00 | 2.33 | 0.78 | 3.00 | 27.82 | 14.49 | 3.00 |
| 1250 | 50.22 | 7.92 | 3.00 | 62.48 | 5.40 | 3.00 | 0.94 | 0.52 | 3.00 | 8.37 | 7.12 | 3.00 |
| 625 | 46.55 | 7.89 | 3.00 | 59.62 | 6.10 | 3.00 | 0.23 | 0.26 | 3.00 | 2.34 | 1.86 | 3.00 |
| 312 | 44.84 | 9.05 | 3.00 | 56.04 | 8.54 | 3.00 | 1.00 | 0.48 | 3.00 | 0.84 | 0.63 | 3.00 |
| 156 | 36.34 | 6.65 | 3.00 | 48.98 | 8.84 | 3.00 | 0.67 | 0.24 | 3.00 | 1.20 | 0.52 | 3.00 |
| 78 | 26.28 | 2.72 | 3.00 | 40.24 | 8.21 | 3.00 | 0.23 | 0.15 | 3.00 | 0.43 | 0.15 | 3.00 |
| 0 | 0.78 | 0.32 | 3.00 | 0.99 | 0.52 | 3.00 | 0.78 | 0.32 | 3.00 | 0.45 | 0.11 | 3.00 |

**Supplementary Table 1 cont.**

|  | SH-SY5Y | | | SH-SY5Y | | | SH-SY5Y | | | SH-SY5Y | | |
| --- | --- | --- | --- | --- | --- | --- | --- | --- | --- | --- | --- | --- |
|  | RSL3 | | | ML162 | | | IKE | | | FINO2 | | |
| [nM] | %CD | SEM | N | %CD | SEM | N | %CD | SEM | N | %CD | SEM | N |
| 20000 | 97.36 | 0.55 | 3.00 | 98.94 | 0.41 | 3.00 | 0.70 | 0.28 | 3.00 | 81.49 | 1.50 | 3.00 |
| 10000 | 81.78 | 1.26 | 3.00 | 88.06 | 3.27 | 3.00 | 0.18 | 0.21 | 3.00 | 47.05 | 5.83 | 3.00 |
| 5000 | 57.60 | 1.25 | 3.00 | 64.12 | 8.91 | 3.00 | -0.05 | 0.19 | 3.00 | 13.73 | 2.67 | 3.00 |
| 2500 | 10.77 | 1.82 | 3.00 | 33.50 | 7.51 | 3.00 | -0.27 | 0.19 | 3.00 | 2.93 | 0.56 | 3.00 |
| 1250 | 4.14 | 0.92 | 3.00 | 7.15 | 2.79 | 3.00 | -0.26 | 0.12 | 3.00 | 1.16 | 0.29 | 3.00 |
| 625 | 2.96 | 0.52 | 3.00 | 3.36 | 0.88 | 3.00 | -0.03 | 0.08 | 3.00 | 0.65 | 0.27 | 3.00 |
| 312 | 2.47 | 0.45 | 3.00 | 2.67 | 0.52 | 3.00 | -0.02 | 0.06 | 3.00 | 0.71 | 0.17 | 3.00 |
| 156 | 2.71 | 0.51 | 3.00 | 2.38 | 0.25 | 3.00 | -0.07 | 0.07 | 3.00 | 0.65 | 0.12 | 3.00 |
| 78 | 2.92 | 0.23 | 3.00 | 1.78 | 0.17 | 3.00 | -0.05 | 0.14 | 3.00 | 0.38 | 0.10 | 3.00 |
| 0 | 0.24 | 0.08 | 3.00 | 0.33 | 0.04 | 3.00 | 0.24 | 0.08 | 3.00 | 0.42 | 0.03 | 3.00 |

**Supplementary Table 2: Overview of the custom-designed list of potential ferroptosis-sensitizing compounds.**

| **Compound** | **Nr in Screen** | **Clinical development** | **Target** | **DOI** | **Cat. Nr.** | **Supplier** |
| --- | --- | --- | --- | --- | --- | --- |
| **Lipid metabolism** |  |  |  |  |  |  |
| TVB-3664 | 1 | Phase II | FASN | 10.1158/0008-5472.can-09-3871 | HY-120062 | MedChemExpress |
| ND-646 | 2 | Preclinical | ACACA | 10.1038/s41556-024-01464-1; 10.1038/nm.4181; 10.1016/j.ejps.2019.105010 | HY-101842 | MedChemExpress |
| MF-438 | 6 | Preclinical | SCD1 | 10.1158/0008-5472.can-19-0369.steroyl-coa; 10.3390/cancers11070948 | S6842 | SelleckChem |
| A939572 | 7 | Preclinical |  |  | HY-50709 | MedChemExpress |
| CAY1566 | 8 | Preclinical |  |  | HY-15823 | MedChemExpress |
| MJ33 | 12 | Preclinical | iPLA2 | 10.1073/pnas.2009201117 | 1007476-63-2 | Cayman Chemicals |
| Bromoenol lactone | 9 | Preclinical |  |  | B1552 | Sigma-Aldrich |
| FKGK18 | 10 | Preclinical |  |  | 13943 | Cayman Chemicals |
| PF-04620110 | 5 | Phase I | DAGT1 | 10.1101/417949 | PZ0207 | Sigma-Aldrich |
| PF-06424439 | 4 | Preclinical | DAGT2 |  | PZ0233 | Sigma-Aldrich |
| Avasimibe | 3 | Phase III | SOAT1 | 10.1073/pnas.1603244113; | PZ0190 | Sigma-Aldrich |
| **Iron metabolism** |  |  |  |  |  |  |
| FeCl2 | 14 | FDA approved | Iron loading | 10.1172/jci.insight.90777 | 372870 | Sigma-Aldrich |
| Hemin | 15 | FDA approved | Iron loading | 10.1172/jci99032 | H9039 | Sigma-Aldrich |
| VIT-2763 | 19 | Phase I | Ferroportin | 10.26355/eurrev_201806_15267; 10.1002/ajh.25670 | HY-112220 | MedChemExpress |
| GÖ 6983 | 20 |  | Iron uptake | 10.1038/onc.2015.32.hspb1 | HY-13689 | MedChemExpress |
| Dihydro-artemisinin | 17 | FDA approved | Ferritinophagy | 10.1038/s41418-019-0352-3; 10.1016/j.freeradbiomed.2018.12.011 | HY-N0176 | MedChemExpress |
| (+)- JQ1 | 23 |  | Ferritinophagy | 10.1038/s41419-019-1564-7 | HY-13030 | MedChemExpress |
| Itraconazole | 16 | FDA approved |  | 10.1002/tox.23031 | S2476 | SelleckChem |
| Siramesine | 18 | Phase II discontinued | Iron storage | 10.1038/cddis.2016.208 | SML0976 | Sigma-Aldrich |
| GW4869 | 21 |  | Exosomal iron export | 10.1016/j.devcel.2019.10.007 | S7609 | SelleckChem |
| **Redox metabolism** |  |  |  |  |  |  |
| iFSP | 24 | Preclinical | FSP1 | 10.1038/s41586-019-1707-0 | / | Conrad Laboratory |
| Cerivastatin | 26 | FDA approved | HMG-CoA reductase | 10.1016/j.cell.2017.09.021; 10.1038/nchembio.2079 | 143201-11-0 | Cayman Chemicals |
| Medroxypro-gesteron acetate | 11 | FDA approved | AKR1C | 10.1038/nchembio.1416; 10.1038/s41419-019-2143-7 | S2567 | SelleckChem |
| 2,4-Diamino-6-hydroxypyrimidine | 29 |  | GCH1 | 10.1021/acscentsci.9b01063 | S3688 | SelleckChem |
| APR-246 | 27 | Phase II |  | 10.1038/ncomms14844 | S7724 | SelleckChem |
| BSO | 25 | Phase I completed | GCL | 10.1146/annurev-cancerbio-030518-055844; 10.1016/j.cell.2013.12.010.regulation | S9728 | SelleckChem |
| Triapine | 33 | Phase II | GS | 10.1186/s12935-020-01689-8 | S7470 | SelleckChem |
| ML385 | 22 |  | NRF2 (via Neh1 DNA binding domain) | 10.1021/acschembio.6b00651 | SML1833 | Sigma-Aldrich |
| Haloperidol | 28 | FDA approved | Sigma 1 Receptor | 10.1016/j.bbrc.2017.07.136 | S1920 | SelleckChem |
| Brequinar | 30 | Phase ll | DHODH | 10.1038/s41586-021-03539-7 | S6626 | SelleckChem |
| Vorinostat | 31 | FDA approved | Class 1 HDAC | 10.3390/cancers12113273 | S6626 | SelleckChem |
| Olaparib | 32 | FDA approved | PARP | 10.1016/j.redox.2021.101928 | S1060 | SelleckChem |

**Supplementary Table 3: Cell death (%) in SK-N-BE2C cells pre-treated 24h with a corresponding compound, followed by 24h ML162 (1µM) or IKE (5µM) ferroptosis induction.**

| **Compound** |  | **/** | **+ML162** | **+IKE** |
| --- | --- | --- | --- | --- |
| MF-438 [SCD1] | %CD | 0.80 | 65.34 | 83.75 |
|  | SE | 0.40 | 2.27 | 6.86 |
|  | N | 2.00 | 1.00 | 2.00 |
|  | p value |  | <0.0001 | <0.0001 |
| A939572 [SCD1] | %CD | 0.18 | 80.41 | 71.76 |
|  | SE | 0.04 | 1.42 | 11.89 |
|  | N | 2.00 | 1.00 | 2.00 |
|  | p value |  | <0.0001 | <0.0001 |
| TVB-3664 [FASN] | %CD | 1.74 | 42.23 | 20.33 |
|  | SE | 0.12 | 0.80 | 7.00 |
|  | N | 2.00 | 1.00 | 2.00 |
|  | p value |  | 0.1749 | 0.2886 |
| ML385 [NRF2] | %CD | 1.23 | 95.99 | 84.71 |
|  | SE | 0.07 | 1.22 | 11.71 |
|  | N | 2.00 | 1.00 | 2.00 |
|  | p value |  | <0.0001 | <0.0001 |
| BSO [GCL] | %CD | 3.03 | 81.88 | 25.56 |
|  | SE | 0.32 | 2.15 | 7.42 |
|  | N | 2.00 | 1.00 | 2.00 |
|  | p value |  | <0.0001 | 0.0735 |
| MPA [AKR1C] | %CD | 31.62 | 5.90 | 63.95 |
|  | SE | 6.06 | 0.96 | 8.78 |
|  | N | 2.00 | 1.00 | 2.00 |
|  | p value |  | <0.0001 | <0.0001 |
| iFSP1 [FSP1] | %CD | 1.50 | 25.57 | 2.43 |
|  | SE | 0.41 | 1.63 | 0.14 |
|  | N | 2.00 | 1.00 | 2.00 |
|  | p value |  | <0.0001 | 0.9993 |
| VIT-2763 [FPN] | %CD | 4.89 | 26.97 | 6.71 |
|  | SE | 0.90 | 1.16 | 1.40 |
|  | N | 2.00 | 1.00 | 2.00 |
|  | p value |  | <0.0001 | >0.9999 |
| DHA [Fe] | %CD | 4.23 | 2.49 | 7.68 |
|  | SE | 0.16 | 0.23 | 1.09 |
|  | N | 2.00 | 1.00 | 2.00 |
|  | p value |  | <0.0001 | >0.9999 |
| Siramesine [Fe] | %CD | 5.34 | 39.67 | 5.25 |
|  | SE | 0.60 | 2.54 | 1.97 |
|  | N | 2.00 | 1.00 | 2.00 |
|  | p value |  | 0.9896 | >0.9999 |
| / | %CD |  | 38.51 | 5.90 |
|  | SE |  | 1.32 | 1.15 |
|  | N |  | 1.00 | 2.00 |

SE represent SEM in case N>1, if not it represents SD. Note technical n=3. p value: Two-way Anova with Dunnett’s multiple comparison

**Supplementary Table 4: Cell death (%) of SK-N-BE(2)C treated for 48h with 1µM of compound.**

|  | MF-438 [SCD1] | | | A939572 [SCD1] | | | TVB-3664 [FASN] | | | ML385 [NRF2] | | |
| --- | --- | --- | --- | --- | --- | --- | --- | --- | --- | --- | --- | --- |
| [µM] | %CD | SEM | N | %CD | SEM | N | %CD | SEM | N | %CD | SEM | N |
| 20 | 2.72 | 1.76 | 2.00 | 2.46 | 2.19 | 2.00 | 2.27 | 0.65 | 2.00 | 4.42 | 0.64 | 2.00 |
| 10 | 3.10 | 1.90 | 2.00 | 1.60 | 1.18 | 2.00 | 2.20 | 0.41 | 2.00 | 3.26 | 0.61 | 2.00 |
| 5 | 3.56 | 2.49 | 2.00 | 2.85 | 2.19 | 2.00 | 2.31 | 0.85 | 2.00 | 4.55 | 0.03 | 2.00 |
| 2.5 | 2.50 | 1.52 | 2.00 | 1.93 | 1.35 | 2.00 | 2.15 | 0.25 | 2.00 | 4.15 | 0.25 | 2.00 |
| 1.25 | 2.95 | 2.21 | 2.00 | 2.39 | 1.57 | 2.00 | 2.00 | 0.14 | 2.00 | 6.61 | 2.30 | 2.00 |
| 0.625 | 3.34 | 2.79 | 2.00 | 3.04 | 2.53 | 2.00 | 1.63 | 0.10 | 2.00 | 3.45 | 0.80 | 2.00 |
| 0.3125 | 3.66 | 2.59 | 2.00 | 2.84 | 1.94 | 2.00 | 1.41 | 0.06 | 2.00 | 3.54 | 0.28 | 2.00 |
| 0 | 3.23 | 2.13 | 2.00 | 3.23 | 2.13 | 2.00 | 3.78 | 2.46 | 2.00 | 4.94 | 0.93 | 2.00 |

**Supplementary Table 4 cont.**

|  | BSO [GCL] | | | MPA [AKR1C] | | | iFSP1 [FSP1] | | | VIT-2763 [FPN] | | |
| --- | --- | --- | --- | --- | --- | --- | --- | --- | --- | --- | --- | --- |
| [µM] | %CD | SEM | N | %CD | SEM | N | %CD | SEM | N | %CD | SEM | N |
| 20 | 3.25 | 1.43 | 2.00 | 4.97 | 1.45 | 2.00 | 5.60 | 1.60 | 2.00 | 6.75 | 3.23 | 2.00 |
| 10 | 3.74 | 1.86 | 2.00 | 2.19 | 0.72 | 2.00 | 2.10 | 0.26 | 2.00 | 0.64 | 0.18 | 2.00 |
| 5 | 3.45 | 1.86 | 2.00 | 1.87 | 0.83 | 2.00 | 2.11 | 0.12 | 2.00 | 1.43 | 0.32 | 2.00 |
| 2.5 | 3.17 | 0.83 | 2.00 | 3.01 | 1.87 | 2.00 | 2.83 | 0.98 | 2.00 | 1.70 | 0.23 | 2.00 |
| 1.25 | 3.46 | 1.15 | 2.00 | 3.52 | 1.95 | 2.00 | 2.80 | 1.53 | 2.00 | 2.26 | 0.93 | 2.00 |
| 0.625 | 3.07 | 0.84 | 2.00 | 3.91 | 2.51 | 2.00 | 2.35 | 1.29 | 2.00 | 1.76 | 0.43 | 2.00 |
| 0.3125 | 3.13 | 1.22 | 2.00 | 4.13 | 2.80 | 2.00 | 1.67 | 0.91 | 2.00 | 1.90 | 0.00 | 2.00 |
| 0 | 4.07 | 2.23 | 2.00 | 3.23 | 2.13 | 2.00 | 2.61 | 2.42 | 2.00 | 4.07 | 2.23 | 2.00 |

**Supplementary Table 4 cont.**

| DHA [Fe] | | | Siramesine [Fe] | | |
| --- | --- | --- | --- | --- | --- |
| %CD | SEM | N | %CD | SEM | N |
| 76.12 | 20.97 | 2.00 | 84.72 | 5.91 | 2.00 |
| 44.11 | 6.81 | 2.00 | 97.73 | 0.79 | 2.00 |
| 29.88 | 3.17 | 2.00 | 4.46 | 0.22 | 2.00 |
| 21.84 | 3.56 | 2.00 | 3.12 | 0.59 | 2.00 |
| 16.37 | 2.42 | 2.00 | 3.13 | 1.07 | 2.00 |
| 9.91 | 0.74 | 2.00 | 2.05 | 0.10 | 2.00 |
| 8.09 | 0.25 | 2.00 | 1.77 | 0.00 | 2.00 |
| 4.94 | 0.93 | 2.00 | 4.07 | 2.23 | 2.00 |

**Supplementary Table 5: Cell death (%) in SK-N-BE(2)C cells pre-treated 72h with PUFAs or PUFA-(e)PLs, followed by 24h IKE (10µM) in 2% FBS media conditions.**

|  |  | **/** | | | | | | | | |
| --- | --- | --- | --- | --- | --- | --- | --- | --- | --- | --- |
|  | [µM] | 0 | 0.16 | 0.31 | 0.62 | 1.25 | 2.5 | 5 | 10 | 20 |
| / | %CD | 2.60 | 2.60 | 2.60 | 2.60 | 2.60 | 2.60 | 2.60 | 2.60 | 2.60 |
|  | SEM | 2.31 | 2.31 | 2.31 | 2.31 | 2.31 | 2.31 | 2.31 | 2.31 | 2.31 |
|  | N | 3.00 | 3.00 | 3.00 | 3.00 | 3.00 | 3.00 | 3.00 | 3.00 | 3.00 |
| AA (20:4) | %CD | 2.65 | 1.37 | 1.48 | 2.27 | 3.09 | 5.16 | 9.56 | 20.56 | 25.35 |
|  | SEM | 2.45 | 1.26 | 1.06 | 1.13 | 1.38 | 2.63 | 3.62 | 7.11 | 4.96 |
|  | N | 3.00 | 3.00 | 3.00 | 3.00 | 3.00 | 3.00 | 3.00 | 3.00 | 3.00 |
| AdA (22:4) | %CD | 2.73 | 1.57 | 2.19 | 2.37 | 1.74 | 3.66 | 9.00 | 25.78 | 40.72 |
|  | SEM | 2.28 | 1.09 | 1.28 | 1.30 | 0.91 | 2.27 | 5.23 | 9.05 | 11.55 |
|  | N | 3.00 | 3.00 | 3.00 | 3.00 | 3.00 | 3.00 | 3.00 | 3.00 | 3.00 |
| DHA (22:6) | %CD | 2.60 | 1.14 | 1.39 | 1.74 | 1.03 | 3.08 | 5.26 | 15.13 | 25.27 |
|  | SEM | 2.31 | 1.42 | 1.14 | 0.68 | 0.12 | 0.41 | 2.26 | 5.15 | 7.66 |
|  | N | 3.00 | 3.00 | 3.00 | 3.00 | 3.00 | 3.00 | 3.00 | 3.00 | 3.00 |
| PE(20:4/20:4) | %CD | 2.72 | 0.75 | 0.83 | 0.45 | -0.13 | -0.11 | 0.63 | 2.89 | 17.48 |
|  | SEM | 2.19 | 0.86 | 1.28 | 0.96 | 1.05 | 0.62 | 0.74 | 0.34 | 1.08 |
|  | N | 3.00 | 3.00 | 3.00 | 3.00 | 3.00 | 3.00 | 3.00 | 3.00 | 3.00 |
| PC(20:4/20:4) | %CD | 3.39 | 0.77 | 0.59 | 1.03 | 0.89 | 1.41 | 1.70 | 3.55 | 11.24 |
|  | SEM | 2.98 | 0.65 | 0.73 | 0.44 | 0.86 | 1.36 | 0.53 | 0.81 | 1.81 |
|  | N | 3.00 | 3.00 | 3.00 | 3.00 | 3.00 | 3.00 | 3.00 | 3.00 | 3.00 |
| PE(18:0/20:4) | %CD | 3.05 | 0.35 | 0.68 | 1.13 | 0.41 | 0.21 | -0.21 | -0.09 | 0.81 |
|  | SEM | 2.27 | 0.64 | 0.94 | 0.80 | 0.80 | 0.51 | 0.42 | 1.34 | 2.13 |
|  | N | 3.00 | 3.00 | 3.00 | 3.00 | 3.00 | 3.00 | 3.00 | 3.00 | 3.00 |
| PC(18:0/20:4) | %CD | 3.21 | 1.41 | 0.20 | 0.81 | 0.56 | 1.21 | 0.83 | 1.10 | 2.34 |
|  | SEM | 2.23 | 1.65 | 0.73 | 0.64 | 0.60 | 1.10 | 1.21 | 1.53 | 2.41 |
|  | N | 3.00 | 3.00 | 3.00 | 3.00 | 3.00 | 3.00 | 3.00 | 3.00 | 3.00 |
| PE(18plasm/20:4) | %CD | 4.04 | 3.51 | 2.98 | 3.64 | 3.08 | 2.32 | 1.87 | 2.23 | 0.50 |
|  | SEM | 0.58 | 1.29 | 0.19 | 0.54 | 0.17 | 0.17 | 0.20 | 0.54 | 0.08 |
|  | N | 3.00 | 3.00 | 3.00 | 3.00 | 3.00 | 3.00 | 3.00 | 3.00 | 3.00 |
| PC(18plasm/20:4) | %CD | 2.79 | 3.49 | 2.39 | 3.37 | 1.57 | 1.99 | 1.95 | 1.30 | 3.03 |
|  | SEM | 0.79 | 0.71 | 0.53 | 1.06 | 0.46 | 0.52 | 0.66 | 0.29 | 0.72 |
|  | N | 3.00 | 3.00 | 3.00 | 3.00 | 3.00 | 3.00 | 3.00 | 3.00 | 3.00 |
| PE(18plasm/22:6) | %CD | 1.86 | 2.42 | 2.06 | 1.52 | 2.57 | 2.24 | 1.03 | 1.62 | 2.40 |
|  | SEM | 0.37 | 0.67 | 0.55 | 0.21 | 0.93 | 0.74 | 0.18 | 0.86 | 1.27 |
|  | N | 3.00 | 3.00 | 3.00 | 3.00 | 3.00 | 3.00 | 3.00 | 3.00 | 3.00 |
| PC(18plasm/22:6) | %CD | 4.32 | 3.98 | 3.84 | 4.00 | 3.33 | 3.00 | 2.22 | 1.59 | 2.06 |
|  | SEM | 0.60 | 0.78 | 1.47 | 1.34 | 1.22 | 1.36 | 0.78 | 0.61 | 0.59 |
|  | N | 3.00 | 3.00 | 3.00 | 3.00 | 3.00 | 3.00 | 3.00 | 3.00 | 3.00 |

**Supplementary Table 5 cont.**

|  |  | **IKE** | | | | | | | | |
| --- | --- | --- | --- | --- | --- | --- | --- | --- | --- | --- |
|  | [µM] | 0 | 0.16 | 0.31 | 0.62 | 1.25 | 2.5 | 5 | 10 | 20 |
| / | %CD | 13.26 | 13.26 | 13.26 | 13.26 | 13.26 | 13.26 | 13.26 | 13.26 | 13.26 |
|  | SEM | 1.92 | 1.92 | 1.92 | 1.92 | 1.92 | 1.92 | 1.92 | 1.92 | 1.92 |
|  | N | 3.00 | 3.00 | 3.00 | 3.00 | 3.00 | 3.00 | 3.00 | 3.00 | 3.00 |
| AA (20:4) | %CD | 12.58 | 12.31 | 14.75 | 18.37 | 24.40 | 38.22 | 55.66 | 100.00 | 100.00 |
|  | SEM | 1.99 | 2.64 | 3.62 | 5.35 | 8.31 | 10.17 | 8.79 | 0.00 | 0.00 |
|  | N | 3.00 | 3.00 | 3.00 | 3.00 | 3.00 | 3.00 | 3.00 | 3.00 | 3.00 |
| AdA (22:4) | %CD | 14.00 | 14.49 | 15.85 | 19.94 | 23.84 | 35.76 | 54.47 | 89.99 | 100.00 |
|  | SEM | 2.16 | 2.99 | 3.94 | 6.00 | 6.87 | 10.08 | 11.86 | 9.25 | 0.00 |
|  | N | 3.00 | 3.00 | 3.00 | 3.00 | 3.00 | 3.00 | 3.00 | 3.00 | 3.00 |
| DHA (22:6) | %CD | 13.26 | 13.15 | 13.79 | 16.59 | 20.96 | 31.09 | 45.12 | 88.44 | 100.00 |
|  | SEM | 1.92 | 2.52 | 3.01 | 4.22 | 5.32 | 10.34 | 13.67 | 11.56 | 0.00 |
|  | N | 3.00 | 3.00 | 3.00 | 3.00 | 3.00 | 3.00 | 3.00 | 3.00 | 3.00 |
| PE(20:4/20:4) | %CD | 8.11 | 7.96 | 7.90 | 8.37 | 8.73 | 10.43 | 12.85 | 19.42 | 100.00 |
|  | SEM | 1.02 | 0.81 | 1.42 | 1.46 | 1.30 | 2.07 | 1.76 | 1.50 | 0.00 |
|  | N | 3.00 | 3.00 | 3.00 | 3.00 | 3.00 | 3.00 | 3.00 | 3.00 | 3.00 |
| PC(20:4/20:4) | %CD | 11.10 | 10.87 | 11.52 | 12.27 | 13.90 | 17.03 | 24.48 | 37.16 | 100.00 |
|  | SEM | 1.93 | 2.37 | 2.76 | 3.24 | 3.43 | 4.50 | 6.57 | 6.74 | 0.00 |
|  | N | 3.00 | 3.00 | 3.00 | 3.00 | 3.00 | 3.00 | 3.00 | 3.00 | 3.00 |
| PE(18:0/20:4) | %CD | 12.27 | 11.11 | 12.72 | 13.29 | 13.17 | 14.11 | 16.37 | 17.55 | 18.06 |
|  | SEM | 2.19 | 2.31 | 2.42 | 3.41 | 3.08 | 3.29 | 3.50 | 3.00 | 2.07 |
|  | N | 3.00 | 3.00 | 3.00 | 3.00 | 3.00 | 3.00 | 3.00 | 3.00 | 3.00 |
| PC(18:0/20:4) | %CD | 15.35 | 12.01 | 11.52 | 12.35 | 12.27 | 11.79 | 12.96 | 12.33 | 12.80 |
|  | SEM | 2.17 | 1.35 | 2.09 | 2.43 | 2.77 | 2.27 | 1.55 | 1.48 | 1.23 |
|  | N | 3.00 | 3.00 | 3.00 | 3.00 | 3.00 | 3.00 | 3.00 | 3.00 | 3.00 |
| PE(18plasm/20:4) | %CD | 5.17 | 6.90 | 7.11 | 7.34 | 8.25 | 8.89 | 10.56 | 12.15 | 13.28 |
|  | SEM | 0.46 | 0.80 | 1.58 | 1.63 | 1.90 | 2.50 | 3.23 | 3.38 | 2.79 |
|  | N | 3.00 | 3.00 | 3.00 | 3.00 | 3.00 | 3.00 | 3.00 | 3.00 | 3.00 |
| PC(18plasm/20:4) | %CD | 6.02 | 6.63 | 6.80 | 7.50 | 8.16 | 7.98 | 8.72 | 10.95 | 14.73 |
|  | SEM | 2.07 | 2.62 | 2.38 | 2.71 | 3.25 | 3.20 | 3.60 | 4.74 | 4.74 |
|  | N | 3.00 | 3.00 | 3.00 | 3.00 | 3.00 | 3.00 | 3.00 | 3.00 | 3.00 |
| PE(18plasm/22:6) | %CD | 6.53 | 6.09 | 7.08 | 6.96 | 9.03 | 9.00 | 10.11 | 16.08 | 21.27 |
|  | SEM | 2.31 | 1.69 | 2.11 | 1.78 | 1.56 | 2.76 | 3.22 | 4.54 | 3.96 |
|  | N | 3.00 | 3.00 | 3.00 | 3.00 | 3.00 | 3.00 | 3.00 | 3.00 | 3.00 |
| PC(18plasm/22:6) | %CD | 7.04 | 6.52 | 7.26 | 8.42 | 7.74 | 7.29 | 7.95 | 9.79 | 12.87 |
|  | SEM | 1.66 | 1.83 | 1.89 | 2.89 | 2.41 | 2.56 | 2.98 | 3.36 | 3.38 |
|  | N | 3.00 | 3.00 | 3.00 | 3.00 | 3.00 | 3.00 | 3.00 | 3.00 | 3.00 |

**Supplementary Table 6: Cell death (%) induced in IMR-32 cells treated 72h with different PUFAs or PUFA-PLs, followed by 24h IKE (10µM) exposure at 2% FBS media conditions.**

|  |  | **/** | | | | **IKE** | | | |
| --- | --- | --- | --- | --- | --- | --- | --- | --- | --- |
|  | [µM] | 0 | 1.25 | 2.5 | 5 | 0 | 1.25 | 2.5 | 5 |
| AA (C20:4) | %CD | 2.37 | 2.53 | 7.13 | 24.93 | 20.12 | 16.26 | 20.98 | 36.16 |
|  | SEM | 0.53 | 0.99 | 2.79 | 5.64 | 7.73 | 5.18 | 3.28 | 5.67 |
|  | N | 3.00 | 3.00 | 3.00 | 3.00 | 3.00 | 3.00 | 3.00 | 3.00 |
| AdA (C22:4) | %CD | 4.36 | 15.51 | 31.83 | 52.15 | 22.93 | 39.83 | 61.59 | 75.87 |
|  | SEM | 2.37 | 9.56 | 21.62 | 17.64 | 9.64 | 8.52 | 19.62 | 12.59 |
|  | N | 3.00 | 3.00 | 3.00 | 3.00 | 3.00 | 3.00 | 3.00 | 3.00 |
| DHA (C22:6) | %CD | 1.54 | 5.69 | 11.86 | 43.28 | 20.48 | 24.35 | 36.49 | 66.69 |
|  | SEM | 0.94 | 1.44 | 4.16 | 16.34 | 8.35 | 8.64 | 9.27 | 19.02 |
|  | N | 3.00 | 3.00 | 3.00 | 3.00 | 3.00 | 3.00 | 3.00 | 3.00 |
| PE(C20:4/C20:4) | %CD | 2.28 | 2.32 | 1.57 | 3.18 | 22.88 | 19.16 | 19.07 | 20.41 |
|  | SEM | 1.11 | 1.40 | 0.47 | 0.75 | 6.91 | 5.37 | 5.64 | 4.96 |
|  | N | 3.00 | 3.00 | 3.00 | 3.00 | 3.00 | 3.00 | 3.00 | 3.00 |
| PC(C20:4/C20:4) | %CD | 2.62 | 2.64 | 6.65 | 22.83 | 25.17 | 20.55 | 25.98 | 50.68 |
|  | SEM | 0.74 | 1.50 | 5.16 | 20.75 | 9.44 | 5.69 | 6.63 | 25.09 |
|  | N | 3.00 | 3.00 | 3.00 | 3.00 | 3.00 | 3.00 | 3.00 | 3.00 |
| PE(C18:0/C20:4) | %CD | 5.20 | 3.23 | 3.15 | 1.23 | 30.03 | 25.12 | 23.19 | 21.82 |
|  | SEM | 0.69 | 1.70 | 1.64 | 0.72 | 14.17 | 11.24 | 10.14 | 12.25 |
|  | N | 3.00 | 3.00 | 3.00 | 3.00 | 3.00 | 3.00 | 3.00 | 3.00 |
| PC(C18:0/C20:4) | %CD | 6.56 | 3.12 | 4.52 | 2.19 | 25.91 | 19.91 | 18.36 | 17.30 |
|  | SEM | 0.54 | 1.44 | 0.42 | 0.13 | 10.50 | 5.42 | 5.31 | 6.50 |
|  | N | 3.00 | 3.00 | 3.00 | 3.00 | 3.00 | 3.00 | 3.00 | 3.00 |

**Supplementary Table 7: Cell death (%) induced in SH-EP cells treated 72h with different PUFAs or PUFA-PLs, followed by 24h IKE (0.5µM) exposure at 2% FBS media conditions.**

|  |  | **/** | | | | **IKE** | | | |
| --- | --- | --- | --- | --- | --- | --- | --- | --- | --- |
|  | [µM] | 0 | 1.25 | 2.5 | 5 | 0 | 1.25 | 2.5 | 5 |
| AA (20:4) | %CD | 3.99 | 2.75 | 3.30 | 7.34 | 2.27 | 2.60 | 3.46 | 8.45 |
|  | SEM | 1.38 | 0.84 | 0.62 | 2.97 | 0.43 | 0.30 | 1.04 | 3.65 |
|  | N | 3.00 | 3.00 | 3.00 | 3.00 | 3.00 | 3.00 | 3.00 | 3.00 |
| AdA (22:4) | %CD | 2.67 | 4.60 | 4.41 | 4.25 | 2.93 | 5.03 | 4.31 | 4.03 |
|  | SEM | 0.78 | 2.08 | 1.66 | 1.18 | 1.51 | 1.86 | 1.36 | 1.68 |
|  | N | 3.00 | 3.00 | 3.00 | 3.00 | 3.00 | 3.00 | 3.00 | 3.00 |
| DHA (22:6) | %CD | 2.30 | 3.73 | 3.91 | 3.43 | 2.93 | 4.62 | 3.98 | 4.85 |
|  | SEM | 0.94 | 1.98 | 2.27 | 1.30 | 1.51 | 1.91 | 1.52 | 1.14 |
|  | N | 3.00 | 3.00 | 3.00 | 3.00 | 3.00 | 3.00 | 3.00 | 3.00 |
| PE(20:4/20:4) | %CD | 4.76 | 5.54 | 6.11 | 5.11 | 4.82 | 5.16 | 5.08 | 4.92 |
|  | SEM | 0.92 | 1.36 | 1.09 | 1.92 | 0.92 | 1.23 | 0.91 | 1.72 |
|  | N | 3.00 | 3.00 | 3.00 | 3.00 | 3.00 | 3.00 | 3.00 | 3.00 |
| PC(20:4/20:4) | %CD | 2.87 | 5.71 | 4.92 | 4.40 | 4.82 | 3.77 | 4.62 | 4.29 |
|  | SEM | 0.76 | 2.41 | 1.04 | 0.99 | 0.92 | 1.59 | 1.51 | 0.91 |
|  | N | 3.00 | 3.00 | 3.00 | 3.00 | 3.00 | 3.00 | 3.00 | 3.00 |
| PE(18:0/20:4) | %CD | 2.67 | 5.43 | 4.35 | 4.38 | 2.93 | 2.55 | 2.67 | 3.35 |
|  | SEM | 0.78 | 2.41 | 1.71 | 1.01 | 1.51 | 0.67 | 0.79 | 0.98 |
|  | N | 3.00 | 3.00 | 3.00 | 3.00 | 3.00 | 3.00 | 3.00 | 3.00 |
| PC(18:0/20:4) | %CD | 2.87 | 4.95 | 4.58 | 2.81 | 4.82 | 4.55 | 3.71 | 1.64 |
|  | SEM | 0.76 | 1.82 | 1.55 | 0.72 | 0.92 | 1.60 | 1.49 | 0.13 |
|  | N | 3.00 | 3.00 | 3.00 | 3.00 | 3.00 | 3.00 | 3.00 | 3.00 |

**Supplementary Table 8: Cell death (%) induced in IMR-32 cells after 72h AA treatment, followed by 24h IKE (10µM) exposure 2% media conditions.**

|  | AA [µM] | 0 | 0.16 | 0.31 | 0.62 | 1.25 | 2.5 | 5 | 10 | 20 |
| --- | --- | --- | --- | --- | --- | --- | --- | --- | --- | --- |
| / | %CD | 2.37 | 2.62 | 1.35 | 1.46 | 2.53 | 7.13 | 24.93 | 69.85 | 76.98 |
|  | SEM | 0.53 | 0.43 | 0.65 | 0.35 | 0.99 | 2.79 | 5.64 | 5.23 | 4.71 |
|  | N | 3.00 | 3.00 | 3.00 | 3.00 | 3.00 | 3.00 | 3.00 | 3.00 | 3.00 |
|  | p value |  | >0.9999 | >0.9999 | >0.9999 | >0.9999 | 0.9734 | 0.0082 | <0,0001 | <0,0001 |
| IKE | %CD | 20.12 | 17.59 | 17.58 | 16.71 | 16.26 | 20.98 | 36.16 | 100.00 | 100.00 |
|  | SEM | 7.73 | 7.62 | 7.35 | 6.28 | 5.18 | 3.28 | 5.67 | 0.00 | 0.00 |
|  | N | 3.00 | 3.00 | 3.00 | 3.00 | 3.00 | 3.00 | 3.00 | 3.00 | 3.00 |
|  | p value |  | 0.9996 | 0.9995 | 0.9965 | 0.9922 | >0.9999 | 0.0964 | <0.0001 | <0.0001 |

p value: Two-way Anova with Dunnett’s multiple comparison.

**Supplementary Table 9: Cell death (%) induced in SH-EP cells after 72h AA treatment, followed by 24h IKE (0.5µM) exposure 2% media conditions.**

|  | AA [µM] | 0 | 0.16 | 0.31 | 0.62 | 1.25 | 2.5 | 5 | 10 | 20 |
| --- | --- | --- | --- | --- | --- | --- | --- | --- | --- | --- |
| / | %CD | 3.99 | 5.32 | 3.30 | 3.08 | 2.75 | 3.30 | 7.34 | 29.52 | 65.99 |
|  | SEM | 1.38 | 2.82 | 1.26 | 0.57 | 0.84 | 0.62 | 2.97 | 13.84 | 18.65 |
|  | N | 3.00 | 3.00 | 3.00 | 3.00 | 3.00 | 3.00 | 3.00 | 3.00 | 3.00 |
|  | p value |  | >0.9999 | >0.9999 | >0.9999 | >0.9999 | >0.9999 | >0.9999 | 0.22 | <0.0001 |
| IKE | %CD | 2.27 | 2.91 | 3.90 | 2.52 | 2.60 | 3.46 | 8.45 | 41.21 | 69.73 |
|  | SEM | 0.43 | 0.66 | 0.96 | 0.57 | 0.30 | 1.04 | 3.65 | 19.85 | 19.16 |
|  | N | 3.00 | 3.00 | 3.00 | 3.00 | 3.00 | 3.00 | 3.00 | 3.00 | 3.00 |
|  | p value |  | >0.9999 | >0.9999 | >0.9999 | >0.9999 | >0.9999 | 0.9974 | 0.02 | <0.0001 |

p value: Two-way Anova with Dunnett’s multiple comparison.

**Supplementary Table 10: Cell death (%) induced after combination pre-treatment of AA and MF-438 at different concentrations in SH-SY5Y cells, followed by 24h RSL3 (5µM) exposure.**

|  | **MF-438 [nM]** | 10000 | | | 5000 | | | 2500 | | | 1250 | | | 625 | | |
| --- | --- | --- | --- | --- | --- | --- | --- | --- | --- | --- | --- | --- | --- | --- | --- | --- |
|  | **AA [µM] ↓** | %CD | SEM | N | %CD | SEM | N | %CD | SEM | N | %CD | SEM | N | %CD | SEM | N |
| +RSL3 | 100 | 100.00 | 0.00 | 3.00 | 99.33 | 0.67 | 3.00 | 100.00 | 0.00 | 3.00 | 99.67 | 0.33 | 3.00 | 98.67 | 1.33 | 3.00 |
|  | 50 | 97.33 | 2.67 | 3.00 | 99.33 | 0.67 | 3.00 | 99.67 | 0.33 | 3.00 | 99.00 | 1.00 | 3.00 | 98.67 | 1.33 | 3.00 |
|  | 25 | 96.67 | 3.33 | 3.00 | 100.00 | 0.00 | 3.00 | 98.33 | 1.67 | 3.00 | 98.33 | 1.67 | 3.00 | 96.00 | 4.00 | 3.00 |
|  | 12.5 | 96.33 | 3.67 | 3.00 | 98.00 | 2.00 | 3.00 | 97.67 | 2.33 | 3.00 | 89.48 | 2.18 | 3.00 | 87.11 | 2.48 | 3.00 |
|  | 6.25 | 95.67 | 4.33 | 3.00 | 89.65 | 1.71 | 3.00 | 90.48 | 2.52 | 3.00 | 87.88 | 2.26 | 3.00 | 83.96 | 1.46 | 3.00 |
|  | 0 | 95.67 | 4.33 | 3.00 | 82.08 | 3.88 | 3.00 | 86.21 | 4.01 | 3.00 | 81.13 | 3.99 | 3.00 | 76.58 | 3.96 | 3.00 |
| / | 100 | 36.38 | 7.18 | 3.00 | 32.31 | 9.54 | 3.00 | 24.24 | 8.35 | 3.00 | 18.00 | 6.60 | 3.00 | 12.15 | 4.52 | 3.00 |
|  | 50 | 30.63 | 6.49 | 3.00 | 17.47 | 1.93 | 3.00 | 11.20 | 1.50 | 3.00 | 7.43 | 0.97 | 3.00 | 4.46 | 0.31 | 3.00 |
|  | 25 | 26.02 | 4.76 | 3.00 | 15.53 | 1.04 | 3.00 | 9.99 | 0.59 | 3.00 | 6.63 | 0.03 | 3.00 | 3.73 | 0.18 | 3.00 |
|  | 12.5 | 23.76 | 3.88 | 3.00 | 14.73 | 0.88 | 3.00 | 9.21 | 0.73 | 3.00 | 5.97 | 0.66 | 3.00 | 3.21 | 0.17 | 3.00 |
|  | 6.25 | 25.11 | 4.91 | 3.00 | 15.64 | 1.72 | 3.00 | 10.98 | 1.07 | 3.00 | 6.83 | 0.37 | 3.00 | 3.63 | 0.12 | 3.00 |
|  | 0 | 35.11 | 8.91 | 3.00 | 26.61 | 6.56 | 3.00 | 18.97 | 5.37 | 3.00 | 13.06 | 4.33 | 3.00 | 8.55 | 2.83 | 3.00 |

**Supplementary Table 10 cont.**

|  | **MF-438 [nM]** | 312 | | | 156 | | | 78 | | | 39 | | | 0 | | |
| --- | --- | --- | --- | --- | --- | --- | --- | --- | --- | --- | --- | --- | --- | --- | --- | --- |
|  | **AA [µM] ↓** | %CD | SEM | N | %CD | SEM | N | %CD | SEM | N | %CD | SEM | N | %CD | SEM | N |
| +RSL3 | 100 | 97.33 | 2.67 | 3.00 | 92.67 | 7.33 | 3.00 | 97.33 | 2.67 | 3.00 | 97.33 | 2.67 | 3.00 | 97.33 | 2.67 | 3.00 |
|  | 50 | 97.67 | 2.33 | 3.00 | 95.33 | 4.67 | 3.00 | 94.00 | 6.00 | 3.00 | 95.67 | 4.33 | 3.00 | 89.00 | 11.00 | 3.00 |
|  | 25 | 90.93 | 3.17 | 3.00 | 88.41 | 3.15 | 3.00 | 83.94 | 1.66 | 3.00 | 81.30 | 2.14 | 3.00 | 71.88 | 5.94 | 3.00 |
|  | 12.5 | 83.58 | 2.79 | 3.00 | 78.47 | 2.41 | 3.00 | 75.03 | 1.02 | 3.00 | 72.31 | 1.90 | 3.00 | 58.58 | 1.12 | 3.00 |
|  | 6.25 | 78.39 | 1.94 | 3.00 | 78.11 | 3.94 | 3.00 | 71.05 | 3.52 | 3.00 | 68.74 | 3.40 | 3.00 | 52.74 | 2.17 | 3.00 |
|  | 0 | 70.37 | 2.24 | 3.00 | 71.65 | 7.68 | 3.00 | 62.71 | 2.65 | 3.00 | 59.55 | 2.23 | 3.00 | 49.09 | 0.51 | 3.00 |
| / | 100 | 8.61 | 3.66 | 3.00 | 10.03 | 4.76 | 3.00 | 5.95 | 2.76 | 3.00 | 6.40 | 2.90 | 3.00 | 10.97 | 5.23 | 3.00 |
|  | 50 | 2.00 | 0.34 | 3.00 | 1.81 | 0.07 | 3.00 | 0.83 | 0.03 | 3.00 | 0.78 | 0.18 | 3.00 | 1.04 | 0.40 | 3.00 |
|  | 25 | 2.06 | 0.21 | 3.00 | 1.54 | 0.17 | 3.00 | 0.79 | 0.26 | 3.00 | 0.35 | 0.16 | 3.00 | 0.68 | 0.23 | 3.00 |
|  | 12.5 | 1.88 | 0.13 | 3.00 | 1.19 | 0.23 | 3.00 | 0.39 | 0.08 | 3.00 | 0.20 | 0.20 | 3.00 | 0.61 | 0.23 | 3.00 |
|  | 6.25 | 2.07 | 0.31 | 3.00 | 1.10 | 0.35 | 3.00 | 0.75 | 0.38 | 3.00 | 0.25 | 0.24 | 3.00 | 1.04 | 0.36 | 3.00 |
|  | 0 | 5.75 | 2.22 | 3.00 | 4.18 | 1.59 | 3.00 | 3.30 | 1.67 | 3.00 | 3.39 | 1.72 | 3.00 | 5.06 | 2.21 | 3.00 |

**Supplementary Table 11: Cell death (%) induced after combination pre-treatment of AA and TVB-3664 at different concentrations in SH-SY5Y cells, followed by 24h RSL3 (5µM) exposure.**

|  | **TVB-3664 [nM]** | 10000 | | | 5000 | | | 2500 | | | 1250 | | | 625 | | |
| --- | --- | --- | --- | --- | --- | --- | --- | --- | --- | --- | --- | --- | --- | --- | --- | --- |
|  | **AA [µM] ↓** | %CD | SEM | N | %CD | SEM | N | %CD | SEM | N | %CD | SEM | N | %CD | SEM | N |
| +RSL3 | 100 | 100.00 | 0.00 | 3.00 | 100.00 | 0.00 | 3.00 | 100.00 | 0.00 | 3.00 | 100.00 | 0.00 | 3.00 | 100.00 | 0.00 | 3.00 |
|  | 50 | 100.00 | 0.00 | 3.00 | 97.34 | 2.66 | 3.00 | 98.87 | 1.13 | 3.00 | 96.82 | 1.62 | 3.00 | 98.97 | 1.03 | 3.00 |
|  | 25 | 100.00 | 0.00 | 3.00 | 99.02 | 0.98 | 3.00 | 93.38 | 1.11 | 3.00 | 94.15 | 0.34 | 3.00 | 96.08 | 1.73 | 3.00 |
|  | 12.5 | 78.10 | 3.27 | 3.00 | 80.15 | 2.10 | 3.00 | 83.11 | 1.76 | 3.00 | 82.74 | 0.69 | 3.00 | 80.23 | 2.83 | 3.00 |
|  | 6.25 | 65.91 | 4.34 | 3.00 | 69.56 | 4.52 | 3.00 | 68.63 | 3.73 | 3.00 | 70.40 | 1.85 | 3.00 | 70.81 | 2.48 | 3.00 |
|  | 0 | 52.67 | 4.20 | 3.00 | 51.15 | 5.79 | 3.00 | 50.40 | 7.08 | 3.00 | 53.24 | 5.55 | 3.00 | 50.61 | 5.90 | 3.00 |
| / | 100 | 29.72 | 11.06 | 3.00 | 17.13 | 4.09 | 3.00 | 14.15 | 2.52 | 3.00 | 14.52 | 3.63 | 3.00 | 10.44 | 0.68 | 3.00 |
|  | 50 | 9.59 | 1.66 | 3.00 | 6.35 | 0.51 | 3.00 | 5.54 | 0.53 | 3.00 | 4.59 | 0.12 | 3.00 | 4.69 | 0.88 | 3.00 |
|  | 25 | 7.94 | 1.28 | 3.00 | 5.60 | 0.79 | 3.00 | 4.34 | 0.78 | 3.00 | 4.62 | 0.50 | 3.00 | 3.78 | 0.76 | 3.00 |
|  | 12.5 | 7.74 | 0.96 | 3.00 | 5.29 | 0.85 | 3.00 | 4.67 | 0.75 | 3.00 | 4.84 | 1.00 | 3.00 | 4.22 | 0.71 | 3.00 |
|  | 6.25 | 7.25 | 1.39 | 3.00 | 5.41 | 0.57 | 3.00 | 4.98 | 0.69 | 3.00 | 4.20 | 0.22 | 3.00 | 4.55 | 0.44 | 3.00 |
|  | 0 | 11.32 | 3.91 | 3.00 | 8.13 | 1.66 | 3.00 | 6.81 | 1.74 | 3.00 | 6.68 | 1.21 | 3.00 | 5.89 | 1.09 | 3.00 |

**Supplementary Table 11 cont.**

|  | **TVB-3664 [nM]** | 312 | | | 156 | | | 78 | | | 39 | | | 0 | | |
| --- | --- | --- | --- | --- | --- | --- | --- | --- | --- | --- | --- | --- | --- | --- | --- | --- |
|  | **AA [µM] ↓** | %CD | SEM | N | %CD | SEM | N | %CD | SEM | N | %CD | SEM | N | %CD | SEM | N |
| +RSL3 | 100 | 100.00 | 0.00 | 3.00 | 100.00 | 0.00 | 3.00 | 100.00 | 0.00 | 3.00 | 100.00 | 0.00 | 3.00 | 100.00 | 0.00 | 3.00 |
|  | 50 | 94.22 | 0.40 | 3.00 | 97.04 | 1.51 | 3.00 | 98.66 | 1.34 | 3.00 | 99.04 | 0.96 | 3.00 | 100.00 | 0.00 | 3.00 |
|  | 25 | 94.08 | 1.44 | 3.00 | 93.10 | 0.98 | 3.00 | 92.67 | 1.46 | 3.00 | 94.52 | 1.97 | 3.00 | 81.25 | 2.72 | 3.00 |
|  | 12.5 | 81.65 | 0.37 | 3.00 | 74.89 | 2.05 | 3.00 | 79.59 | 1.44 | 3.00 | 78.45 | 2.36 | 3.00 | 63.48 | 2.24 | 3.00 |
|  | 6.25 | 70.69 | 1.69 | 3.00 | 67.69 | 2.68 | 3.00 | 69.06 | 2.47 | 3.00 | 64.39 | 1.72 | 3.00 | 49.95 | 5.00 | 3.00 |
|  | 0 | 48.38 | 4.47 | 3.00 | 49.37 | 4.04 | 3.00 | 49.62 | 4.87 | 3.00 | 47.61 | 2.22 | 3.00 | 41.45 | 5.52 | 3.00 |
| / | 100 | 11.41 | 2.10 | 3.00 | 12.71 | 4.63 | 3.00 | 10.39 | 3.35 | 3.00 | 14.68 | 8.00 | 3.00 | 11.17 | 7.63 | 3.00 |
|  | 50 | 4.57 | 0.26 | 3.00 | 4.32 | 0.33 | 3.00 | 3.02 | 0.45 | 3.00 | 2.30 | 0.28 | 3.00 | 0.68 | 0.40 | 3.00 |
|  | 25 | 3.86 | 0.60 | 3.00 | 3.87 | 0.49 | 3.00 | 2.94 | 0.57 | 3.00 | 1.56 | 0.27 | 3.00 | 0.04 | 0.04 | 3.00 |
|  | 12.5 | 3.82 | 0.62 | 3.00 | 3.11 | 0.34 | 3.00 | 2.75 | 0.43 | 3.00 | 1.91 | 0.65 | 3.00 | 0.57 | 0.22 | 3.00 |
|  | 6.25 | 4.58 | 0.46 | 3.00 | 3.28 | 0.25 | 3.00 | 3.12 | 0.40 | 3.00 | 2.22 | 0.33 | 3.00 | 0.68 | 0.10 | 3.00 |
|  | 0 | 6.86 | 1.09 | 3.00 | 5.60 | 1.36 | 3.00 | 5.08 | 1.14 | 3.00 | 4.46 | 1.22 | 3.00 | 3.20 | 1.49 | 3.00 |

**Supplementary Table 12: Cell death (%) induced after combination pre-treatment of AA and ND-646 at different concentrations in SH-SY5Y cells, followed by 24h RSL3 (5µM) exposure.**

|  | **ND-646 [nM]** | 10000 | | | 5000 | | | 2500 | | | 1250 | | | 625 | | |
| --- | --- | --- | --- | --- | --- | --- | --- | --- | --- | --- | --- | --- | --- | --- | --- | --- |
|  | **AA [µM] ↓** | %CD | SEM | N | %CD | SEM | N | %CD | SEM | N | %CD | SEM | N | %CD | SEM | N |
| +RSL3 | 100 | 97.02 | 2.98 | 3.00 | 94.65 | 5.35 | 3.00 | 91.61 | 4.32 | 3.00 | 89.57 | 5.51 | 3.00 | 90.96 | 4.54 | 3.00 |
|  | 50 | 76.02 | 2.69 | 3.00 | 86.85 | 2.76 | 3.00 | 90.52 | 2.17 | 3.00 | 90.12 | 1.01 | 3.00 | 94.48 | 2.83 | 3.00 |
|  | 25 | 78.64 | 2.53 | 3.00 | 83.10 | 4.71 | 3.00 | 84.51 | 2.81 | 3.00 | 87.04 | 2.73 | 3.00 | 89.77 | 3.04 | 3.00 |
|  | 12.5 | 66.78 | 4.50 | 3.00 | 78.50 | 5.35 | 3.00 | 80.64 | 3.45 | 3.00 | 81.01 | 4.62 | 3.00 | 83.65 | 5.37 | 3.00 |
|  | 6.25 | 66.77 | 4.37 | 3.00 | 68.40 | 2.84 | 3.00 | 72.82 | 4.60 | 3.00 | 75.03 | 3.27 | 3.00 | 75.82 | 3.59 | 3.00 |
|  | 0 | 34.84 | 8.91 | 3.00 | 43.35 | 9.46 | 3.00 | 48.30 | 5.50 | 3.00 | 55.22 | 0.91 | 3.00 | 55.70 | 0.40 | 3.00 |
| / | 100 | 16.09 | 2.37 | 3.00 | 13.35 | 1.28 | 3.00 | 10.98 | 1.81 | 3.00 | 11.37 | 2.40 | 3.00 | 10.28 | 2.66 | 3.00 |
|  | 50 | 10.06 | 1.36 | 3.00 | 7.51 | 2.34 | 3.00 | 7.07 | 2.66 | 3.00 | 6.83 | 1.89 | 3.00 | 7.80 | 2.29 | 3.00 |
|  | 25 | 7.26 | 0.63 | 3.00 | 6.62 | 1.73 | 3.00 | 5.48 | 2.28 | 3.00 | 6.37 | 1.78 | 3.00 | 7.64 | 1.48 | 3.00 |
|  | 12.5 | 5.29 | 0.38 | 3.00 | 5.23 | 0.80 | 3.00 | 5.23 | 1.67 | 3.00 | 5.13 | 1.50 | 3.00 | 5.83 | 1.70 | 3.00 |
|  | 6.25 | 6.66 | 0.23 | 3.00 | 4.20 | 0.48 | 3.00 | 4.63 | 1.19 | 3.00 | 4.69 | 1.16 | 3.00 | 5.02 | 1.43 | 3.00 |
|  | 0 | 8.99 | 0.70 | 3.00 | 7.48 | 0.54 | 3.00 | 7.69 | 0.69 | 3.00 | 6.98 | 1.08 | 3.00 | 7.73 | 1.05 | 3.00 |

**Supplementary Table 12 cont.**

|  | **ND-646 [nM]** | 312 | | | 156 | | | 78 | | | 39 | | | 0 | | |
| --- | --- | --- | --- | --- | --- | --- | --- | --- | --- | --- | --- | --- | --- | --- | --- | --- |
|  | **AA [µM] ↓** | %CD | SEM | N | %CD | SEM | N | %CD | SEM | N | %CD | SEM | N | %CD | SEM | N |
| +RSL3 | 100 | 90.75 | 5.65 | 3.00 | 89.48 | 5.84 | 3.00 | 91.07 | 4.49 | 3.00 | 90.75 | 5.44 | 3.00 | 100.00 | 0.00 | 3.00 |
|  | 50 | 92.09 | 2.62 | 3.00 | 90.64 | 2.14 | 3.00 | 91.02 | 2.58 | 3.00 | 90.44 | 4.29 | 3.00 | 100.00 | 0.00 | 3.00 |
|  | 25 | 87.27 | 3.31 | 3.00 | 86.16 | 2.57 | 3.00 | 84.21 | 3.94 | 3.00 | 85.78 | 4.56 | 3.00 | 80.43 | 1.38 | 3.00 |
|  | 12.5 | 82.39 | 3.27 | 3.00 | 81.94 | 4.82 | 3.00 | 78.54 | 3.93 | 3.00 | 80.34 | 4.45 | 3.00 | 54.87 | 4.18 | 3.00 |
|  | 6.25 | 74.55 | 1.51 | 3.00 | 72.49 | 2.52 | 3.00 | 71.58 | 0.95 | 3.00 | 71.78 | 2.62 | 3.00 | 46.99 | 1.93 | 3.00 |
|  | 0 | 57.80 | 0.78 | 3.00 | 54.22 | 1.86 | 3.00 | 50.66 | 1.93 | 3.00 | 47.12 | 7.65 | 3.00 | 38.05 | 9.09 | 3.00 |
| / | 100 | 10.77 | 2.26 | 3.00 | 9.79 | 2.80 | 3.00 | 10.91 | 2.45 | 3.00 | 10.91 | 2.62 | 3.00 | 7.60 | 2.71 | 3.00 |
|  | 50 | 7.24 | 2.19 | 3.00 | 6.76 | 2.48 | 3.00 | 6.76 | 2.36 | 3.00 | 6.28 | 1.70 | 3.00 | 1.08 | 0.76 | 3.00 |
|  | 25 | 5.41 | 1.58 | 3.00 | 5.57 | 2.04 | 3.00 | 6.07 | 1.69 | 3.00 | 4.90 | 1.53 | 3.00 | 0.13 | 0.13 | 3.00 |
|  | 12.5 | 5.62 | 0.78 | 3.00 | 4.86 | 0.42 | 3.00 | 4.59 | 1.65 | 3.00 | 4.43 | 1.24 | 3.00 | 0.72 | 0.48 | 3.00 |
|  | 6.25 | 4.94 | 0.91 | 3.00 | 5.23 | 0.67 | 3.00 | 4.56 | 1.48 | 3.00 | 3.64 | 1.40 | 3.00 | 0.66 | 0.25 | 3.00 |
|  | 0 | 7.62 | 1.61 | 3.00 | 6.98 | 1.63 | 3.00 | 6.17 | 2.12 | 3.00 | 6.36 | 0.83 | 3.00 | 3.83 | 1.26 | 3.00 |

**Supplementary Table 13: Cell death (%) induced after combination pre-treatment of AA and iFSP1 at different concentrations in SH-SY5Y cells, followed by 24h RSL3 (5µM) exposure.**

|  | **iFSP1 [nM]** | 10000 | 5000 | 2500 | 1250 | 625 | 312 | 156 | 78 | 39 | 0 |
| --- | --- | --- | --- | --- | --- | --- | --- | --- | --- | --- | --- |
|  | **AA [µM] ↓** | %CD | %CD | %CD | %CD | %CD | %CD | %CD | %CD | %CD | %CD |
| +RSL3 | 100 | 100 | 100 | 100 | 100 | 100 | 100 | 100 | 100 | 100 | 100 |
|  | 50 | 100 | 100 | 100 | 100 | 100 | 100 | 100 | 100 | 100 | 100 |
|  | 25 | 47.32 | 85.19 | 86.95 | 84.18 | 87.89 | 87.10 | 86.67 | 86.34 | 81.51 | 79.50 |
|  | 12,5 | 38.35 | 73.98 | 65.08 | 71.04 | 71.45 | 64.29 | 63.62 | 63.62 | 65.56 | 61.20 |
|  | 6,25 | 33.72 | 55.69 | 58.57 | 56.49 | 59.17 | 59.51 | 53.77 | 52.67 | 51.24 | 49.30 |
|  | 0 | 27.85 | 40.26 | 44.10 | 46.43 | 48.55 | 44.93 | 44.57 | 40.81 | 41.15 | 40.44 |
| / | 100 | 42.06 | 10.37 | 21.45 | 7.31 | 13.96 | 20.89 | 15.84 | 18.10 | 18.42 | 13.76 |
|  | 50 | 11.35 | 1.17 | 1.61 | 2.22 | 1.94 | 3.66 | 1.82 | 2.11 | 3.28 | 2.01 |
|  | 25 | 8.67 | 0.89 | 1.10 | 1.36 | 1.30 | 0.83 | 1.29 | 0.83 | 0.98 | 0.72 |
|  | 12,5 | 17.79 | 0.36 | 1.48 | 1.18 | 1.27 | 1.36 | 1.72 | 1.36 | 1.11 | 1.22 |
|  | 6,25 | 19.49 | 0.03 | 0.00 | 0.69 | 1.25 | 1.30 | 1.21 | 0.76 | 2.15 | 0.50 |
|  | 0 | 22.98 | 1.56 | 1.51 | 2.17 | 2.63 | 2.66 | 1.77 | 2.09 | 2.12 | 2.69 |

**Supplementary Table 14: Cell death (%) induced after combination pre-treatment of AA and icFSP1 at different concentrations in SH-SY5Y cells, followed by 24h RSL3 (5µM) exposure.**

|  | **icFSP1 [nM]** | 10000 | 5000 | 2500 | 1250 | 625 | 312 | 156 | 78 | 39 | 0 |
| --- | --- | --- | --- | --- | --- | --- | --- | --- | --- | --- | --- |
|  | **AA [µM] ↓** | %CD | %CD | %CD | %CD | %CD | %CD | %CD | %CD | %CD | %CD |
| +RSL3 | 100 | 100 | 100 | 100 | 100 | 100 | 100 | 100 | 100 | 100 | 100 |
|  | 50 | 100 | 100 | 100 | 100 | 100 | 100 | 100 | 100 | 100 | 100 |
|  | 25 | 81.70 | 95.59 | 93.88 | 97.48 | 94.95 | 88.89 | 92.02 | 89.96 | 90.34 | 89.11 |
|  | 12,5 | 65.33 | 69.58 | 75.35 | 79.38 | 71.41 | 70.98 | 67.78 | 64.94 | 64.28 | 65.50 |
|  | 6,25 | 50.20 | 56.83 | 59.25 | 54.71 | 58.12 | 54.26 | 53.33 | 51.39 | 50.94 | 49.05 |
|  | 0 | 50.61 | 51.28 | 49.28 | 45.30 | 44.76 | 43.49 | 42.41 | 41.31 | 38.84 | 38.83 |
| / | 100 | 15.52 | 7.49 | 6.45 | 9.70 | 9.07 | 11.43 | 28.92 | 10.92 | 15.55 | 22.48 |
|  | 50 | 5.12 | 1.96 | 2.27 | 1.52 | 2.10 | 3.07 | 1.66 | 0.76 | 1.79 | 2.67 |
|  | 25 | 2.88 | 1.61 | 1.47 | 1.36 | 0.92 | 2.86 | 0.92 | 0.96 | 0.86 | 0.31 |
|  | 12,5 | 2.88 | 2.27 | 1.04 | 1.22 | 1.18 | 1.94 | 0.79 | 0.51 | 0.61 | 0.57 |
|  | 6,25 | 2.81 | 1.70 | 1.22 | 1.07 | 0.19 | 0.81 | 1.64 | 1.12 | 0.00 | 1.30 |
|  | 0 | 5.82 | 2.52 | 2.71 | 2.73 | 2.20 | 2.67 | 1.58 | 1.86 | 1.42 | 2.35 |

**Supplementary Table 15: Cell death (%) induced after combination pre-treatment of AA and viFSP1 at different concentrations in SH-SY5Y cells, followed by 24h RSL3 (5µM) exposure.**

|  | **viFSP1 [nM]** | 10000 | 5000 | 2500 | 1250 | 625 | 312 | 156 | 78 | 39 | 0 |
| --- | --- | --- | --- | --- | --- | --- | --- | --- | --- | --- | --- |
|  | **AA [µM] ↓** | %CD | %CD | %CD | %CD | %CD | %CD | %CD | %CD | %CD | %CD |
| +RSL3 | 100 | 100 | 100 | 100 | 100 | 100 | 100 | 100 | 100 | 100 | 100 |
|  | 50 | 100 | 100 | 100 | 100 | 100 | 100 | 100 | 100 | 100 | 100 |
|  | 25 | 83.22 | 87.78 | 90.50 | 85.56 | 91.80 | 88.12 | 86.97 | 85.35 | 80.49 | 76.23 |
|  | 12,5 | 60.25 | 64.83 | 73.12 | 62.96 | 71.96 | 66.05 | 60.61 | 61.77 | 57.99 | 60.30 |
|  | 6,25 | 49.66 | 51.11 | 50.79 | 50.06 | 52.71 | 49.72 | 50.06 | 52.05 | 45.49 | 48.27 |
|  | 0 | 40.84 | 40.82 | 39.56 | 40.14 | 40.05 | 38.73 | 40.52 | 37.65 | 36.83 | 35.37 |
| / | 100 | 49.68 | 22.11 | 13.81 | 52.05 | 17.88 | 20.63 | 42.00 | 23.54 | 46.00 | 42.80 |
|  | 50 | 4.30 | 0.87 | 1.32 | 1.86 | 2.15 | 2.59 | 1.99 | 1.92 | 1.93 | 1.27 |
|  | 25 | 4.48 | 1.61 | 2.39 | 2.76 | 2.48 | 2.54 | 2.15 | 1.38 | 1.61 | 2.13 |
|  | 12,5 | 2.42 | 1.77 | 1.49 | 0.73 | 1.10 | 0.98 | 0.82 | 0.69 | 0.29 | 0.44 |
|  | 6,25 | 2.01 | 1.31 | 0.81 | 1.80 | 0.77 | 2.00 | 0.88 | 0.75 | 0.00 | 0.27 |
|  | 0 | 5.03 | 1.47 | 2.90 | 2.69 | 2.44 | 2.94 | 2.86 | 2.83 | 1.96 | 2.24 |

**Supplementary Table 16: Cell death (%) induced after combination pre-treatment of Cy7-AA-LNP and MF-438 at different concentrations in SH-SY5Y cells, followed by 24h RSL3 (2.5µM) exposure.**

|  | MF-438 [nM] | 10000 | | | 5000 | | | 2500 | | | 1250 | | | 625 | | |
| --- | --- | --- | --- | --- | --- | --- | --- | --- | --- | --- | --- | --- | --- | --- | --- | --- |
|  | Cy-7-AA-LNP [µM] ↓ | %CD | SEM | N | %CD | SEM | N | %CD | SEM | N | %CD | SEM | N | %CD | SEM | N |
| +RSL3 | 100 | 105.53 | 5.08 | 3.00 | 101.92 | 3.90 | 3.00 | 101.15 | 3.45 | 3.00 | 98.77 | 4.85 | 3.00 | 100.68 | 6.48 | 3.00 |
|  | 50 | 103.13 | 4.22 | 3.00 | 102.42 | 3.60 | 3.00 | 101.47 | 3.62 | 3.00 | 100.58 | 3.29 | 3.00 | 99.96 | 3.70 | 3.00 |
|  | 25 | 103.34 | 3.25 | 3.00 | 101.47 | 3.33 | 3.00 | 100.41 | 2.28 | 3.00 | 98.00 | 3.04 | 3.00 | 97.77 | 2.42 | 3.00 |
|  | 12.5 | 100.96 | 3.56 | 3.00 | 99.61 | 3.09 | 3.00 | 97.39 | 3.19 | 3.00 | 93.40 | 2.76 | 3.00 | 90.48 | 3.87 | 3.00 |
|  | 6.25 | 99.26 | 3.54 | 3.00 | 98.81 | 2.46 | 3.00 | 93.39 | 2.84 | 3.00 | 87.53 | 4.59 | 3.00 | 82.55 | 4.48 | 3.00 |
|  | 0 | 99.74 | 2.74 | 3.00 | 90.12 | 2.86 | 3.00 | 77.25 | 11.22 | 3.00 | 69.75 | 13.41 | 3.00 | 64.04 | 15.72 | 3.00 |
| / | 100 | 16.33 | 11.37 | 3.00 | 10.18 | 8.08 | 3.00 | 8.21 | 5.74 | 3.00 | 5.41 | 3.30 | 3.00 | 3.61 | 2.12 | 3.00 |
|  | 50 | 11.55 | 9.44 | 3.00 | 8.79 | 6.06 | 3.00 | 6.33 | 3.72 | 3.00 | 4.95 | 2.31 | 3.00 | 4.27 | 1.40 | 3.00 |
|  | 25 | 19.49 | 18.13 | 3.00 | 8.56 | 5.69 | 3.00 | 6.96 | 3.66 | 3.00 | 5.13 | 1.78 | 3.00 | 2.81 | 0.85 | 3.00 |
|  | 12.5 | 22.70 | 19.69 | 3.00 | 9.61 | 5.96 | 3.00 | 6.98 | 3.52 | 3.00 | 5.68 | 2.51 | 3.00 | 4.89 | 1.43 | 3.00 |
|  | 6.25 | 19.48 | 16.29 | 3.00 | 9.39 | 5.77 | 3.00 | 7.01 | 3.44 | 3.00 | 6.13 | 2.65 | 3.00 | 4.78 | 1.55 | 3.00 |
|  | 0 | 27.71 | 24.53 | 3.00 | 12.31 | 9.12 | 3.00 | 10.18 | 6.55 | 3.00 | 5.74 | 2.32 | 3.00 | 3.58 | 1.10 | 3.00 |

**Supplementary Table 16 cont.**

|  | MF-438 [nM] | 312 | | | 156 | | | 78 | | | 39 | | | 0 | | |
| --- | --- | --- | --- | --- | --- | --- | --- | --- | --- | --- | --- | --- | --- | --- | --- | --- |
|  | Cy-7-AA-LNP [µM] ↓ | %CD | SEM | N | %CD | SEM | N | %CD | SEM | N | %CD | SEM | N | %CD | SEM | N |
| +RSL3 | 100 | 96.51 | 4.43 | 3.00 | 95.85 | 6.84 | 3.00 | 95.13 | 7.11 | 3.00 | 95.34 | 6.13 | 3.00 | 98.87 | 5.61 | 3.00 |
|  | 50 | 98.62 | 3.16 | 3.00 | 98.10 | 4.23 | 3.00 | 97.36 | 4.30 | 3.00 | 96.61 | 4.24 | 3.00 | 97.33 | 3.59 | 3.00 |
|  | 25 | 94.80 | 3.21 | 3.00 | 92.63 | 3.97 | 3.00 | 92.31 | 4.39 | 3.00 | 90.84 | 3.53 | 3.00 | 92.17 | 4.86 | 3.00 |
|  | 12.5 | 87.18 | 3.32 | 3.00 | 83.54 | 3.72 | 3.00 | 82.88 | 4.33 | 3.00 | 81.89 | 3.80 | 3.00 | 71.41 | 5.76 | 3.00 |
|  | 6.25 | 78.36 | 3.62 | 3.00 | 75.62 | 3.59 | 3.00 | 75.81 | 4.04 | 3.00 | 70.94 | 3.24 | 3.00 | 58.11 | 6.51 | 3.00 |
|  | 0 | 53.70 | 11.87 | 3.00 | 49.50 | 11.38 | 3.00 | 47.95 | 10.41 | 3.00 | 46.67 | 10.51 | 3.00 | 37.03 | 4.15 | 3.00 |
| / | 100 | 3.83 | 1.42 | 3.00 | 3.63 | 0.72 | 3.00 | 3.17 | 0.54 | 3.00 | 3.09 | 0.45 | 3.00 | 1.91 | 0.08 | 3.00 |
|  | 50 | 3.33 | 0.68 | 3.00 | 3.74 | 0.66 | 3.00 | 3.36 | 0.77 | 3.00 | 3.13 | 0.70 | 3.00 | 3.64 | 0.93 | 3.00 |
|  | 25 | 4.25 | 1.22 | 3.00 | 3.83 | 0.68 | 3.00 | 3.58 | 0.56 | 3.00 | 3.48 | 1.09 | 3.00 | 2.61 | 1.88 | 3.00 |
|  | 12.5 | 3.84 | 0.88 | 3.00 | 3.90 | 0.76 | 3.00 | 3.98 | 0.94 | 3.00 | 3.67 | 0.88 | 3.00 | 4.43 | 1.72 | 3.00 |
|  | 6.25 | 4.38 | 0.76 | 3.00 | 4.26 | 0.74 | 3.00 | 4.00 | 0.78 | 3.00 | 3.19 | 0.84 | 3.00 | 4.51 | 1.82 | 3.00 |
|  | 0 | 3.85 | 0.74 | 3.00 | 4.41 | 0.91 | 3.00 | 4.28 | 1.21 | 3.00 | 3.99 | 0.96 | 3.00 | 3.84 | 1.79 | 3.00 |

# Epilipidomics Inclusion List

| **Compound** | **Formula** | **Adduct** | **m/z** | **z** |
| --- | --- | --- | --- | --- |
| 17:0-14:1 PC-d5 | C_39_H_71_D_5_NO_8_P | +HCOO | 767,5598929 | 1 |
| 17:0-14:1 PE-d5 | C_36_H_65_D_5_NO_8_P | -H | 679,5074634 | 1 |
| 17:0-16:1 PC-d5 | C_41_H_75_D_5_NO_8_P | +HCOO | 795,5911931 | 1 |
| 17:0-16:1 PE-d5 | C_38_H_69_D_5_NO_8_P | -H | 707,5387636 | 1 |
| 17:0-18:1 PC-d5 | C_43_H_79_D_5_NO_8_P | +HCOO | 823,6224932 | 1 |
| 17:0-18:1 PE-d5 | C_40_H_73_D_5_NO_8_P | -H | 735,5700637 | 1 |
| 17:0-20:3 PC-d5 | C_45_H_79_D_5_NO_8_P | +HCOO | 847,6224932 | 1 |
| 17:0-20:3 PE-d5 | C_42_H_73_D_5_NO_8_P | -H | 759,5700637 | 1 |
| 17:0-22:4 PC-d5 | C_47_H_81_D_5_NO_8_P | +HCOO | 873,6381433 | 1 |
| 17:0-22:4 PE-d5 | C_44_H_75_D_5_NO_8_P | -H | 785,5857138 | 1 |
| PC(14:0_11:1<2oxo>) | C33H60O10NP | +HCOO | 706.393138 | 1 |
| PC(14:0_11:1<OH,oxo>) | C33H62O10NP | +HCOO | 708.408788 | 1 |
| PC(14:0_19:4<COOH>) | C41H72O10NP | -H | 768.481559 | 1 |
| PC(14:0_19:4<OH,oxo>) | C41H72O10NP | +HCOO | 814.487038 | 1 |
| PC(14:0_19:4<oxo>) | C41H72O9NP | +HCOO | 798.492124 | 1 |
| PC(14:0_8:0<oxo>) | C30H58O9NP | +HCOO | 652.382573 | 1 |
| PC(16:0_10:1<2oxo>) | C34H62O10NP | +HCOO | 720.408788 | 1 |
| PC(16:0_10:1<COOH>) | C34H64O10NP | -H | 676.418959 | 1 |
| PC(16:0_10:1<OH,oxo>) | C34H64O10NP | +HCOO | 722.424438 | 1 |
| PC(16:0_10:1<oxo>) | C34H64O9NP | +HCOO | 706.429523 | 1 |
| PC(16:0_10:2<2oxo>) | C34H60O10NP | +HCOO | 718.393138 | 1 |
| PC(16:0_10:2<COOH>) | C34H62O10NP | -H | 674.403309 | 1 |
| PC(16:0_10:2<oxo>) | C34H62O9NP | +HCOO | 704.413873 | 1 |
| PC(16:0_11:1<COOH>) | C35H66O10NP | -H | 690.434609 | 1 |
| PC(16:0_11:1<OH,oxo>) | C35H66O10NP | +HCOO | 736.440088 | 1 |
| PC(16:0_11:1<oxo>) | C35H66O9NP | +HCOO | 720.445174 | 1 |
| PC(16:0_11:2<2oxo>) | C35H62O10NP | +HCOO | 732.408788 | 1 |
| PC(16:0_11:2<COOH>) | C35H64O10NP | -H | 688.418959 | 1 |
| PC(16:0_11:2<OH,oxo>) | C35H64O10NP | +HCOO | 734.424438 | 1 |
| PC(16:0_11:2<oxo>) | C35H64O9NP | +HCOO | 718.429523 | 1 |
| PC(16:0_12:1<2oxo>) | C36H66O10NP | +HCOO | 748.440088 | 1 |
| PC(16:0_12:1<COOH>) | C36H68O10NP | -H | 704.450259 | 1 |
| PC(16:0_12:1<OH,oxo>) | C36H68O10NP | +HCOO | 750.455738 | 1 |
| PC(16:0_12:1<oxo>) | C36H68O9NP | +HCOO | 734.460824 | 1 |
| PC(16:0_13:0<COOH>) | C37H72O10NP | -H | 720.481559 | 1 |
| PC(16:0_13:0<oxo>) | C37H72O9NP | +HCOO | 750.492124 | 1 |
| PC(16:0_13:2<2oxo>) | C37H66O10NP | +HCOO | 760.440088 | 1 |
| PC(16:0_13:2<COOH>) | C37H68O10NP | -H | 716.450259 | 1 |
| PC(16:0_13:2<OH,oxo>) | C37H68O10NP | +HCOO | 762.455738 | 1 |
| PC(16:0_13:2<oxo>) | C37H68O9NP | +HCOO | 746.460824 | 1 |
| PC(16:0_13:3<2oxo>) | C37H64O10NP | +HCOO | 758.424438 | 1 |
| PC(16:0_13:3<COOH>) | C37H66O10NP | -H | 714.434609 | 1 |
| PC(16:0_13:3<oxo>) | C37H66O9NP | +HCOO | 744.445174 | 1 |
| PC(16:0_14:2<COOH>) | C38H70O10NP | -H | 730.465909 | 1 |
| PC(16:0_14:2<OH,oxo>) | C38H70O10NP | +HCOO | 776.471388 | 1 |
| PC(16:0_14:2<oxo>) | C38H70O9NP | +HCOO | 760.476474 | 1 |
| PC(16:0_14:3<2oxo>) | C38H66O10NP | +HCOO | 772.440088 | 1 |
| PC(16:0_14:3<COOH>) | C38H68O10NP | -H | 728.450259 | 1 |
| PC(16:0_14:3<OH,oxo>) | C38H68O10NP | +HCOO | 774.455738 | 1 |
| PC(16:0_14:3<oxo>) | C38H68O9NP | +HCOO | 758.460824 | 1 |
| PC(16:0_16:1<2oxo>) | C40H74O10NP | +HCOO | 804.502688 | 1 |
| PC(16:0_16:1<COOH>) | C40H76O10NP | -H | 760.512859 | 1 |
| PC(16:0_16:1<OH,oxo>) | C40H76O10NP | +HCOO | 806.518338 | 1 |
| PC(16:0_16:1<oxo>) | C40H76O9NP | +HCOO | 790.523424 | 1 |
| PC(16:0_16:3<2oxo>) | C40H70O10NP | +HCOO | 800.471388 | 1 |
| PC(16:0_16:3<COOH>) | C40H72O10NP | -H | 756.481559 | 1 |
| PC(16:0_16:3<OH,oxo>) | C40H72O10NP | +HCOO | 802.487038 | 1 |
| PC(16:0_16:3<oxo>) | C40H72O9NP | +HCOO | 786.492124 | 1 |
| PC(16:0_16:4<2oxo>) | C40H68O10NP | +HCOO | 798.455738 | 1 |
| PC(16:0_16:4<COOH>) | C40H70O10NP | -H | 754.465909 | 1 |
| PC(16:0_16:4<oxo>) | C40H70O9NP | +HCOO | 784.476474 | 1 |
| PC(16:0_18:2<2OH>) | C42H80O10NP | +HCOO | 834.549639 | 1 |
| PC(16:0_18:2<OH,oxo>) | C42H78O10NP | +HCOO | 832.533989 | 1 |
| PC(16:0_18:2<OH>) | C42H80O9NP | +HCOO | 818.554724 | 1 |
| PC(16:0_18:2<oxo>) | C42H78O9NP | +HCOO | 816.539074 | 1 |
| PC(16:0_19:5<2oxo>) | C43H72O10NP | +HCOO | 838.487038 | 1 |
| PC(16:0_19:5<COOH>) | C43H74O10NP | -H | 794.497209 | 1 |
| PC(16:0_19:5<OH,oxo>) | C43H74O10NP | +HCOO | 840.502688 | 1 |
| PC(16:0_19:5<oxo>) | C43H74O9NP | +HCOO | 824.507774 | 1 |
| PC(16:0_20:2<2OH>) | C44H84O10NP | +HCOO | 862.580939 | 1 |
| PC(16:0_20:2<OH,oxo>) | C44H82O10NP | +HCOO | 860.565289 | 1 |
| PC(16:0_20:2<OH>) | C44H84O9NP | +HCOO | 846.586024 | 1 |
| PC(16:0_20:2<oxo>) | C44H82O9NP | +HCOO | 844.570374 | 1 |
| PC(16:0_20:4<2OH>) | C44H80O10NP | +HCOO | 858.549639 | 1 |
| PC(16:0_20:4<OH,oxo>) | C44H78O10NP | +HCOO | 856.533989 | 1 |
| PC(16:0_20:4<OH>) | C44H80O9NP | +HCOO | 842.554724 | 1 |
| PC(16:0_20:4<oxo>) | C44H78O9NP | +HCOO | 840.539074 | 1 |
| PC(16:0_22:2<2OH>) | C46H88O10NP | +HCOO | 890.612239 | 1 |
| PC(16:0_22:2<OH,oxo>) | C46H86O10NP | +HCOO | 888.596589 | 1 |
| PC(16:0_22:2<OH>) | C46H88O9NP | +HCOO | 874.617324 | 1 |
| PC(16:0_22:2<oxo>) | C46H86O9NP | +HCOO | 872.601674 | 1 |
| PC(16:0_22:4<2OH>) | C46H84O10NP | +HCOO | 886.580939 | 1 |
| PC(16:0_22:4<OH,oxo>) | C46H82O10NP | +HCOO | 884.565289 | 1 |
| PC(16:0_22:4<OH>) | C46H84O9NP | +HCOO | 870.586024 | 1 |
| PC(16:0_22:4<oxo>) | C46H82O9NP | +HCOO | 868.570374 | 1 |
| PC(16:0_22:6<2OH>) | C46H80O10NP | +HCOO | 882.549639 | 1 |
| PC(16:0_22:6<OH,oxo>) | C46H78O10NP | +HCOO | 880.533989 | 1 |
| PC(16:0_22:6<OH>) | C46H80O9NP | +HCOO | 866.554724 | 1 |
| PC(16:0_22:6<oxo>) | C46H78O9NP | +HCOO | 864.539074 | 1 |
| PC(16:0_4:0<COOH>) | C28H54O10NP | -H | 594.340708 | 1 |
| PC(16:0_4:0<oxo>) | C28H54O9NP | +HCOO | 624.351273 | 1 |
| PC(16:0_5:0<COOH>) | C29H56O10NP | -H | 608.356359 | 1 |
| PC(16:0_5:0<oxo>) | C29H56O9NP | +HCOO | 638.366923 | 1 |
| PC(16:0_7:0<COOH>) | C31H60O10NP | -H | 636.387659 | 1 |
| PC(16:0_7:0<oxo>) | C31H60O9NP | +HCOO | 666.398223 | 1 |
| PC(16:0_7:1<2oxo>) | C31H56O10NP | +HCOO | 678.361838 | 1 |
| PC(16:0_7:1<COOH>) | C31H58O10NP | -H | 634.372009 | 1 |
| PC(16:0_7:1<OH,oxo>) | C31H58O10NP | +HCOO | 680.377488 | 1 |
| PC(16:0_7:1<oxo>) | C31H58O9NP | +HCOO | 664.382573 | 1 |
| PC(16:0_8:0<COOH>) | C32H62O10NP | -H | 650.403309 | 1 |
| PC(16:0_8:0<oxo>) | C32H62O9NP | +HCOO | 680.413873 | 1 |
| PC(16:0_8:1<2oxo>) | C32H58O10NP | +HCOO | 692.377488 | 1 |
| PC(16:0_8:1<COOH>) | C32H60O10NP | -H | 648.387659 | 1 |
| PC(16:0_8:1<OH,oxo>) | C32H60O10NP | +HCOO | 694.393138 | 1 |
| PC(16:0_8:1<oxo>) | C32H60O9NP | +HCOO | 678.398223 | 1 |
| PC(16:0_9:0<COOH>) | C33H64O10NP | -H | 664.418959 | 1 |
| PC(16:0_9:0<oxo>) | C33H64O9NP | +HCOO | 694.429523 | 1 |
| PC(16:1_11:2<2oxo>) | C35H60O10NP | +HCOO | 730.393138 | 1 |
| PC(16:1_11:2<COOH>) | C35H62O10NP | -H | 686.403309 | 1 |
| PC(16:1_11:2<oxo>) | C35H62O9NP | +HCOO | 716.413873 | 1 |
| PC(16:1_12:1<2oxo>) | C36H64O10NP | +HCOO | 746.424438 | 1 |
| PC(16:1_12:1<COOH>) | C36H66O10NP | -H | 702.434609 | 1 |
| PC(16:1_12:1<oxo>) | C36H66O9NP | +HCOO | 732.445174 | 1 |
| PC(16:1_14:3<2oxo>) | C38H64O10NP | +HCOO | 770.424438 | 1 |
| PC(16:1_14:3<COOH>) | C38H66O10NP | -H | 726.434609 | 1 |
| PC(16:1_14:3<oxo>) | C38H66O9NP | +HCOO | 756.445174 | 1 |
| PC(16:1_17:4<2oxo>) | C41H68O10NP | +HCOO | 810.455738 | 1 |
| PC(16:1_17:4<COOH>) | C41H70O10NP | -H | 766.465909 | 1 |
| PC(16:1_17:4<OH,oxo>) | C41H70O10NP | +HCOO | 812.471388 | 1 |
| PC(16:1_17:4<oxo>) | C41H70O9NP | +HCOO | 796.476474 | 1 |
| PC(16:1_18:2<OH,oxo>) | C42H76O10NP | +HCOO | 830.518338 | 1 |
| PC(16:1_18:2<oxo>) | C42H76O9NP | +HCOO | 814.523424 | 1 |
| PC(16:1_20:4<OH,oxo>) | C44H76O10NP | +HCOO | 854.518338 | 1 |
| PC(16:1_20:4<oxo>) | C44H76O9NP | +HCOO | 838.523424 | 1 |
| PC(16:1_20:5<OH,oxo>) | C44H74O10NP | +HCOO | 852.502688 | 1 |
| PC(16:1_20:5<oxo>) | C44H74O9NP | +HCOO | 836.507774 | 1 |
| PC(16:1_5:0<COOH>) | C29H54O10NP | -H | 606.340708 | 1 |
| PC(16:1_5:0<oxo>) | C29H54O9NP | +HCOO | 636.351273 | 1 |
| PC(16:1_8:1<2oxo>) | C32H56O10NP | +HCOO | 690.361838 | 1 |
| PC(16:1_8:1<COOH>) | C32H58O10NP | -H | 646.372009 | 1 |
| PC(16:1_8:1<oxo>) | C32H58O9NP | +HCOO | 676.382573 | 1 |
| PC(16:1_9:0<COOH>) | C33H62O10NP | -H | 662.403309 | 1 |
| PC(16:1_9:0<oxo>) | C33H62O9NP | +HCOO | 692.413873 | 1 |
| PC(18:0_10:0<COOH>) | C36H70O10NP | -H | 706.465909 | 1 |
| PC(18:0_10:0<oxo>) | C36H70O9NP | +HCOO | 736.476474 | 1 |
| PC(18:0_12:1<COOH>) | C38H72O10NP | -H | 732.481559 | 1 |
| PC(18:0_12:1<OH,oxo>) | C38H72O10NP | +HCOO | 778.487038 | 1 |
| PC(18:0_12:1<oxo>) | C38H72O9NP | +HCOO | 762.492124 | 1 |
| PC(18:0_13:1<2oxo>) | C39H72O10NP | +HCOO | 790.487038 | 1 |
| PC(18:0_13:1<COOH>) | C39H74O10NP | -H | 746.497209 | 1 |
| PC(18:0_13:1<OH,oxo>) | C39H74O10NP | +HCOO | 792.502688 | 1 |
| PC(18:0_13:1<oxo>) | C39H74O9NP | +HCOO | 776.507774 | 1 |
| PC(18:0_16:2<COOH>) | C42H78O10NP | -H | 786.528509 | 1 |
| PC(18:0_22:3<2OH>) | C48H90O10NP | +HCOO | 916.627889 | 1 |
| PC(18:0_22:3<OH,oxo>) | C48H88O10NP | +HCOO | 914.612239 | 1 |
| PC(18:0_22:3<OH>) | C48H90O9NP | +HCOO | 900.632974 | 1 |
| PC(18:0_22:3<oxo>) | C48H88O9NP | +HCOO | 898.617324 | 1 |
| PC(18:0_9:0<COOH>) | C35H68O10NP | -H | 692.450259 | 1 |
| PC(18:0_9:0<oxo>) | C35H68O9NP | +HCOO | 722.460824 | 1 |
| PC(18:2_11:2<2oxo>) | C37H62O10NP | +HCOO | 756.408788 | 1 |
| PC(18:2_11:2<COOH>) | C37H64O10NP | -H | 712.418959 | 1 |
| PC(18:2_11:2<oxo>) | C37H64O9NP | +HCOO | 742.429523 | 1 |
| PC(18:2_14:3<2oxo>) | C40H66O10NP | +HCOO | 796.440088 | 1 |
| PC(18:2_14:3<COOH>) | C40H68O10NP | -H | 752.450259 | 1 |
| PC(18:2_14:3<oxo>) | C40H68O9NP | +HCOO | 782.460824 | 1 |
| PC(18:2_5:0<COOH>) | C31H56O10NP | -H | 632.356359 | 1 |
| PC(18:2_5:0<oxo>) | C31H56O9NP | +HCOO | 662.366923 | 1 |
| PC(18:2_8:1<2oxo>) | C34H58O10NP | +HCOO | 716.377488 | 1 |
| PC(18:2_8:1<COOH>) | C34H60O10NP | -H | 672.387659 | 1 |
| PC(18:2_8:1<oxo>) | C34H60O9NP | +HCOO | 702.398223 | 1 |
| PC(20:0_13:2<2oxo>) | C41H74O10NP | +HCOO | 816.502688 | 1 |
| PC(20:0_13:2<COOH>) | C41H76O10NP | -H | 772.512859 | 1 |
| PC(20:0_13:2<OH,oxo>) | C41H76O10NP | +HCOO | 818.518338 | 1 |
| PC(20:0_13:2<oxo>) | C41H76O9NP | +HCOO | 802.523424 | 1 |
| PC(20:0_16:3<COOH>) | C44H80O10NP | -H | 812.544159 | 1 |
| PC(20:0_22:4<2OH>) | C50H92O10NP | +HCOO | 942.643539 | 1 |
| PC(20:0_22:4<OH,oxo>) | C50H90O10NP | +HCOO | 940.627889 | 1 |
| PC(20:0_22:4<OH>) | C50H92O9NP | +HCOO | 926.648624 | 1 |
| PC(20:0_22:4<oxo>) | C50H90O9NP | +HCOO | 924.632974 | 1 |
| PC(20:4_11:2<2oxo>) | C39H62O10NP | +HCOO | 780.408788 | 1 |
| PC(20:4_11:2<COOH>) | C39H64O10NP | -H | 736.418959 | 1 |
| PC(20:4_11:2<OH,oxo>) | C39H64O10NP | +HCOO | 782.424438 | 1 |
| PC(20:4_11:2<oxo>) | C39H64O9NP | +HCOO | 766.429523 | 1 |
| PC(20:4_14:3<2oxo>) | C42H66O10NP | +HCOO | 820.440088 | 1 |
| PC(20:4_14:3<COOH>) | C42H68O10NP | -H | 776.450259 | 1 |
| PC(20:4_14:3<OH,oxo>) | C42H68O10NP | +HCOO | 822.455738 | 1 |
| PC(20:4_14:3<oxo>) | C42H68O9NP | +HCOO | 806.460824 | 1 |
| PC(20:4_20:4<2OH>) | C48H80O10NP | +HCOO | 906.549639 | 1 |
| PC(20:4_20:4<OH,oxo>) | C48H78O10NP | +HCOO | 904.533989 | 1 |
| PC(20:4_20:4<OH>) | C48H80O9NP | +HCOO | 890.554724 | 1 |
| PC(20:4_20:4<oxo>) | C48H78O9NP | +HCOO | 888.539074 | 1 |
| PC(20:4_5:0<COOH>) | C33H56O10NP | -H | 656.356359 | 1 |
| PC(20:4_5:0<oxo>) | C33H56O9NP | +HCOO | 686.366923 | 1 |
| PC(20:4_8:1<2oxo>) | C36H58O10NP | +HCOO | 740.377488 | 1 |
| PC(20:4_8:1<COOH>) | C36H60O10NP | -H | 696.387659 | 1 |
| PC(20:4_8:1<OH,oxo>) | C36H60O10NP | +HCOO | 742.393138 | 1 |
| PC(20:4_8:1<oxo>) | C36H60O9NP | +HCOO | 726.398223 | 1 |
| PC(O-16:0_11:2<COOH>) | C35H66O9NP | -H | 674.439694 | 1 |
| PC(O-16:0_14:3<COOH>) | C38H70O9NP | -H | 714.470994 | 1 |
| PC(O-16:0_14:3<oxo>) | C38H70O8NP | +HCOO | 744.481559 | 1 |
| PC(O-16:0_20:4<OH>) | C44H82O8NP | +HCOO | 828.575459 | 1 |
| PC(O-16:0_20:4<oxo>) | C44H80O8NP | +HCOO | 826.559809 | 1 |
| PC(O-16:0aa_5:0<COOH>) | C29H58O9NP | -H | 594.377094 | 1 |
| PC(O-16:0_5:0<oxo>) | C29H58O8NP | +HCOO | 624.387659 | 1 |
| PC(O-16:0_8:1<COOH>) | C32H62O9NP | -H | 634.408394 | 1 |
| PC(P-16:0_11:2<COOH>) | C35H64O9NP | -H | 672.424044 | 1 |
| PC(P-16:0_13:0<COOH>) | C37H72O9NP | -H | 704.486644 | 1 |
| PC(P-16:0_13:0<oxo>) | C37H72O8NP | +HCOO | 734.497209 | 1 |
| PC(P-16:0_14:3<COOH>) | C38H68O9NP | -H | 712.455344 | 1 |
| PC(P-16:0_14:3<oxo>) | C38H68O8NP | +HCOO | 742.465909 | 1 |
| PC(P-16:0_16:1<2oxo>) | C40H74O9NP | +HCOO | 788.507774 | 1 |
| PC(P-16:0_16:1<COOH>) | C40H76O9NP | -H | 744.517945 | 1 |
| PC(P-16:0_16:1<oxo>) | C40H76O8NP | +HCOO | 774.528509 | 1 |
| PC(P-16:0_20:4<oxo>) | C44H78O8NP | +HCOO | 824.544159 | 1 |
| PC(P-16:0_22:2<OH>) | C46H88O8NP | +HCOO | 858.62241 | 1 |
| PC(P-16:0_22:2<oxo>) | C46H86O8NP | +HCOO | 856.60676 | 1 |
| PC(P-16:0_5:0<COOH>) | C29H56O9NP | -H | 592.361444 | 1 |
| PC(P-16:0_5:0<oxo>) | C29H56O8NP | +HCOO | 622.372009 | 1 |
| PC(P-16:0_8:1<COOH>) | C32H60O9NP | -H | 632.392744 | 1 |
| PE(16:0_10:1<oxo>) | C31H58O9NP | -H | 618.377094 | 1 |
| PE(16:0_11:2<2oxo>) | C32H56O10NP | -H | 644.356359 | 1 |
| PE(16:0_11:2<oxo>) | C32H58O9NP | -H | 630.377094 | 1 |
| PE(16:0_12:1<2oxo>) | C33H60O10NP | -H | 660.387659 | 1 |
| PE(16:0_12:1<oxo>) | C33H62O9NP | -H | 646.408394 | 1 |
| PE(16:0_13:2<oxo>) | C34H62O9NP | -H | 658.408394 | 1 |
| PE(16:0_14:3<2oxo>) | C35H60O10NP | -H | 684.387659 | 1 |
| PE(16:0_14:3<oxo>) | C35H62O9NP | -H | 670.408394 | 1 |
| PE(16:0_16:3<oxo>) | C37H66O9NP | -H | 698.439694 | 1 |
| PE(16:0_18:2<OH>) | C39H74O9NP | -H | 730.502294 | 1 |
| PE(16:0_18:2<oxo>) | C39H72O9NP | -H | 728.486644 | 1 |
| PE(16:0_20:4<2OH>) | C41H74O10NP | -H | 770.497209 | 1 |
| PE(16:0_20:4<OH>) | C41H74O9NP | -H | 754.502294 | 1 |
| PE(16:0_20:4<oxo>) | C41H72O9NP | -H | 752.486644 | 1 |
| PE(16:0_22:4<2OH>) | C43H78O10NP | -H | 798.528509 | 1 |
| PE(16:0_22:4<OH,oxo>) | C43H76O10NP | -H | 796.512859 | 1 |
| PE(16:0_22:4<OH>) | C43H78O9NP | -H | 782.533595 | 1 |
| PE(16:0_22:4<oxo>) | C43H76O9NP | -H | 780.517945 | 1 |
| PE(16:0_5:0<COOH>) | C26H50O10NP | -H | 566.309408 | 1 |
| PE(16:0_5:0<oxo>) | C26H50O9NP | -H | 550.314494 | 1 |
| PE(16:0_7:0<oxo>) | C28H54O9NP | -H | 578.345794 | 1 |
| PE(16:0_8:1<2oxo>) | C29H52O10NP | -H | 604.325058 | 1 |
| PE(16:0_8:1<oxo>) | C29H54O9NP | -H | 590.345794 | 1 |
| PE(16:0_9:0<oxo>) | C30H58O9NP | -H | 606.377094 | 1 |
| PE(18:0_10:2<2oxo>) | C33H58O10NP | -H | 658.372009 | 1 |
| PE(18:0_10:2<oxo>) | C33H60O9NP | -H | 644.392744 | 1 |
| PE(18:0_11:1<oxo>) | C34H64O9NP | -H | 660.424044 | 1 |
| PE(18:0_13:2<2oxo>) | C36H64O10NP | -H | 700.418959 | 1 |
| PE(18:0_13:2<oxo>) | C36H66O9NP | -H | 686.439694 | 1 |
| PE(18:0_13:3<2oxo>) | C36H62O10NP | -H | 698.403309 | 1 |
| PE(18:0_13:3<oxo>) | C36H64O9NP | -H | 684.424044 | 1 |
| PE(18:0_14:2<oxo>) | C37H68O9NP | -H | 700.455344 | 1 |
| PE(18:0_16:3<2oxo>) | C39H68O10NP | -H | 740.450259 | 1 |
| PE(18:0_16:3<oxo>) | C39H70O9NP | -H | 726.470994 | 1 |
| PE(18:0_16:4<2oxo>) | C39H66O10NP | -H | 738.434609 | 1 |
| PE(18:0_16:4<oxo>) | C39H68O9NP | -H | 724.455344 | 1 |
| PE(18:0_18:2<OH>) | C41H78O9NP | -H | 758.533595 | 1 |
| PE(18:0_18:2<oxo>) | C41H76O9NP | -H | 756.517945 | 1 |
| PE(18:0_19:5<2oxo>) | C42H70O10NP | -H | 778.465909 | 1 |
| PE(18:0_19:5<COOH>) | C42H72O10NP | -H | 780.481559 | 1 |
| PE(18:0_19:5<oxo>) | C42H72O9NP | -H | 764.486644 | 1 |
| PE(18:0_20:3<2OH>) | C43H80O10NP | -H | 800.544159 | 1 |
| PE(18:0_20:3<OH>) | C43H80O9NP | -H | 784.549245 | 1 |
| PE(18:0_22:4<OH>) | C45H82O9NP | -H | 810.564895 | 1 |
| PE(18:0_22:4<oxo>) | C45H80O9NP | -H | 808.549245 | 1 |
| PE(18:0_22:6<2OH>) | C45H78O10NP | -H | 822.528509 | 1 |
| PE(18:0_22:6<OH,oxo>) | C45H76O10NP | -H | 820.512859 | 1 |
| PE(18:0_22:6<OH>) | C45H78O9NP | -H | 806.533595 | 1 |
| PE(18:0_22:6<oxo>) | C45H76O9NP | -H | 804.517945 | 1 |
| PE(18:0_4:0<COOH>) | C27H52O10NP | -H | 580.325058 | 1 |
| PE(18:0_4:0<oxo>) | C27H52O9NP | -H | 564.330144 | 1 |
| PE(18:0_7:1<2oxo>) | C30H54O10NP | -H | 618.340708 | 1 |
| PE(18:0_7:1<COOH>) | C30H56O10NP | -H | 620.356359 | 1 |
| PE(18:0_7:1<oxo>) | C30H56O9NP | -H | 604.361444 | 1 |
| PE(18:0_8:0<oxo>) | C31H60O9NP | -H | 620.392744 | 1 |
| PE(18:1_11:2<2oxo>) | C34H58O10NP | -H | 670.372009 | 1 |
| PE(18:1_11:2<oxo>) | C34H60O9NP | -H | 656.392744 | 1 |
| PE(18:1_14:3<2oxo>) | C37H62O10NP | -H | 710.403309 | 1 |
| PE(18:1_14:3<oxo>) | C37H64O9NP | -H | 696.424044 | 1 |
| PE(18:1_20:4<oxo>) | C43H74O9NP | -H | 778.502294 | 1 |
| PE(18:1_5:0<COOH>) | C28H52O10NP | -H | 592.325058 | 1 |
| PE(18:1_5:0<oxo>) | C28H52O9NP | -H | 576.330144 | 1 |
| PE(18:1_8:1<2oxo>) | C31H54O10NP | -H | 630.340708 | 1 |
| PE(18:1_8:1<oxo>) | C31H56O9NP | -H | 616.361444 | 1 |
| PE(20:1_22:4<2OH>) | C47H84O10NP | -H | 852.575459 | 1 |
| PE(20:1_22:4<OH,oxo>) | C47H82O10NP | -H | 850.559809 | 1 |
| PE(20:1_22:4<OH>) | C47H84O9NP | -H | 836.580545 | 1 |
| PE(20:1_22:4<oxo>) | C47H82O9NP | -H | 834.564895 | 1 |
| PE(P-16:0_10:1<oxo>) | C31H58O8NP | -H | 602.382179 | 1 |
| PE(P-16:0_11:2<2oxo>) | C32H56O9NP | -H | 628.361444 | 1 |
| PE(P-16:0_11:2<oxo>) | C32H58O8NP | -H | 614.382179 | 1 |
| PE(P-16:0_13:2<oxo>) | C34H62O8NP | -H | 642.413479 | 1 |
| PE(P-16:0_14:3<2oxo>) | C35H60O9NP | -H | 668.392744 | 1 |
| PE(P-16:0_14:3<oxo>) | C35H62O8NP | -H | 654.413479 | 1 |
| PE(P-16:0_16:3<oxo>) | C37H66O8NP | -H | 682.44478 | 1 |
| PE(P-16:0_20:4<OH>) | C41H74O8NP | -H | 738.50738 | 1 |
| PE(P-16:0_20:4<oxo>) | C41H72O8NP | -H | 736.49173 | 1 |
| PE(P-16:0_22:4<OH>) | C43H78O8NP | -H | 766.53868 | 1 |
| PE(P-16:0_22:4<oxo>) | C43H76O8NP | -H | 764.52303 | 1 |
| PE(P-16:0_5:0<oxo>) | C26H50O8NP | -H | 534.319579 | 1 |
| PE(P-16:0_7:0<oxo>) | C28H54O8NP | -H | 562.350879 | 1 |
| PE(P-16:0_8:1<2oxo>) | C29H52O9NP | -H | 588.330144 | 1 |
| PE(P-16:0_8:1<oxo>) | C29H54O8NP | -H | 574.350879 | 1 |

# References

1. Grootjans S, Hassannia B, Delrue I, Goossens V, Wiernicki B, Dondelinger Y, et al. A real-time fluorometric method for the simultaneous detection of cell death type and rate. Nat Protoc [Internet]. 2016;11(8):1444–54. Available from: <http://dx.doi.org/10.1038/nprot.2016.085>

2. Toyokuni S, Miyake N, Hiai H, Hagiwara M, Kawakishi S, Osawa T, et al. The monoclonal antibody specific for the 4‐hydroxy‐2‐nonenal histidine adduct. FEBS Lett. 1995;359(2–3):189–91.

3. Ozeki M, Miyagawa-Hayashino A, Akatsuka S, Shirase T, Lee W hua, Uchida K, et al. Susceptibility of actin to modification by 4-hydroxy-2-nonenal. J Chromatogr B. 2005;827(1):119–26.

4. Zheng H, Jiang L, Tsuduki T, Conrad M, Toyokuni S. Embryonal erythropoiesis and aging exploit ferroptosis. Redox Biol. 2021;48:102175.

5. Ni Z, Angelidou G, Hoffmann R, Fedorova M. LPPtiger software for lipidome-specific prediction and identification of oxidized phospholipids from LC-MS datasets. Sci Rep. 2017;7(1):15138.

6. Criscuolo A, Nepachalovich P, Rio DFG del, Lange M, Ni Z, Baroni M, et al. Analytical and computational workflow for in-depth analysis of oxidized complex lipids in blood plasma. Nat Commun. 2022;13(1):6547.

7. MacLean B, Tomazela DM, Shulman N, Chambers M, Finney GL, Frewen B, et al. Skyline: an open source document editor for creating and analyzing targeted proteomics experiments. Bioinformatics. 2010;26(7):966–8.

# 
