# Supplementary material for: Dual lipid modulation overcomes ferroptosis resistance in high-risk neuroblastoma
Source: Cell Death Differ. 2025 Nov 26;33(5):903–13. doi: 10.1038/s41418-025-01623-3 (PMC13156318; doi:10.1038/s41418-025-01623-3)

**Full Western Blot Fig. 1M**

Membrane 1

Contrast used for B-tubulin, includes SCD1 at lower intensities.

**
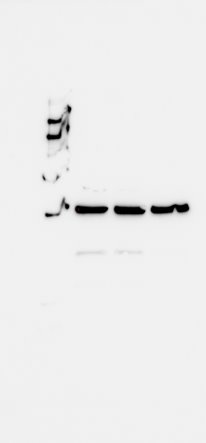
**

Contrast used for SCD1, did not contain Beta-Tubulin yet.

**
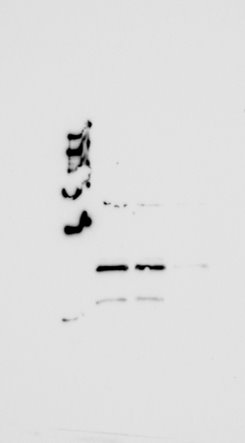
**

Contrast used for GPX4, also contains B-tub and SCD1.

 
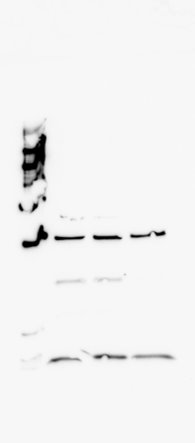


Membrane 2

Contrast used for ACSL4, not treated with B-tubulin antibody yet.

 
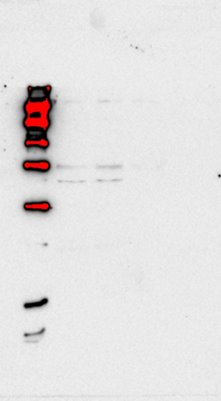


Contrast used B-tubulin, also contains ACSL4 at lower intensities.

**
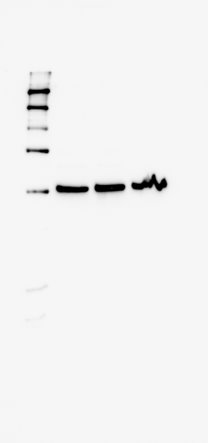
**

Membrane 3

Contrast used for FASN, not treated with B-tubulin antibody yet.

 
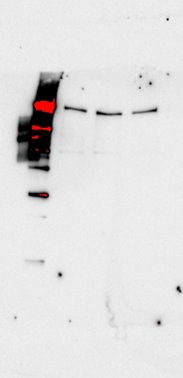


Contrast used for B-tubulin, also contains FASN signal at lower intensities.

 
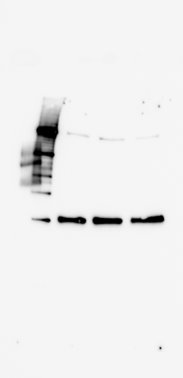


**Full Western Blot Fig. S20D**

Contrast used for B-tubulin, includes SCD1 at lower intensities.

**
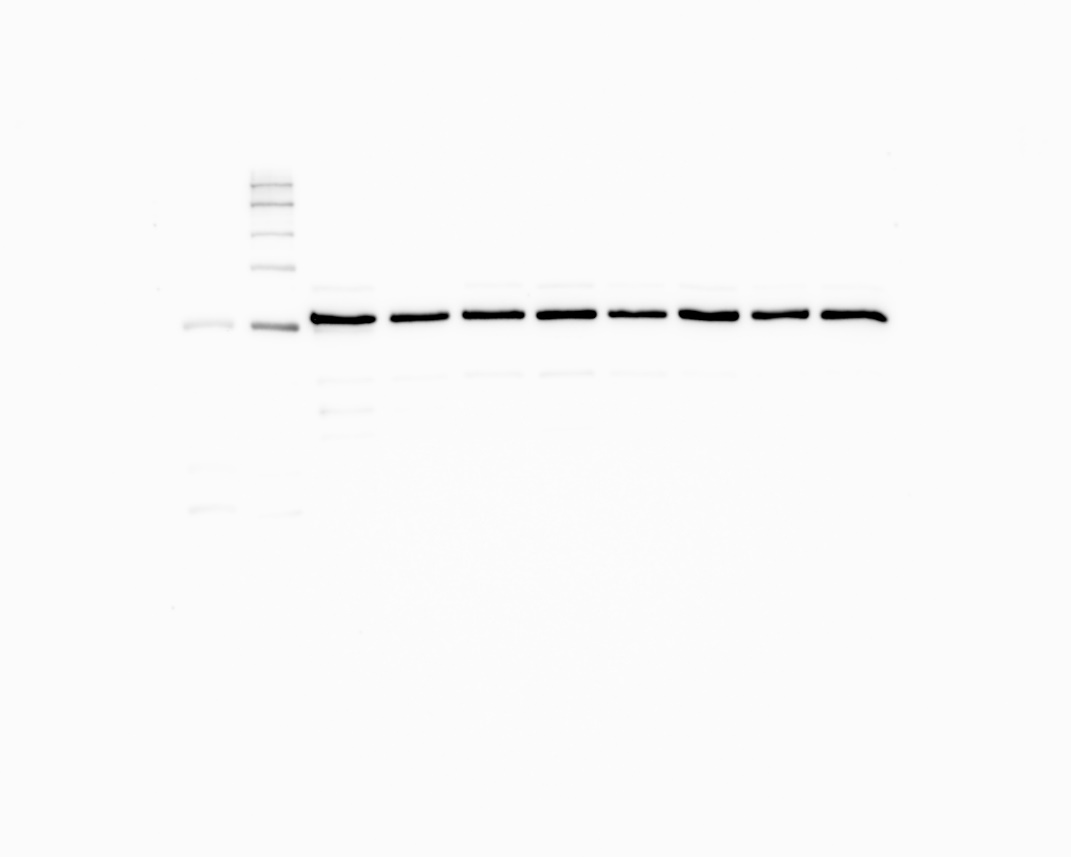
**

Contrast used for SCD1, not treated with B-tubulin antibody yet.
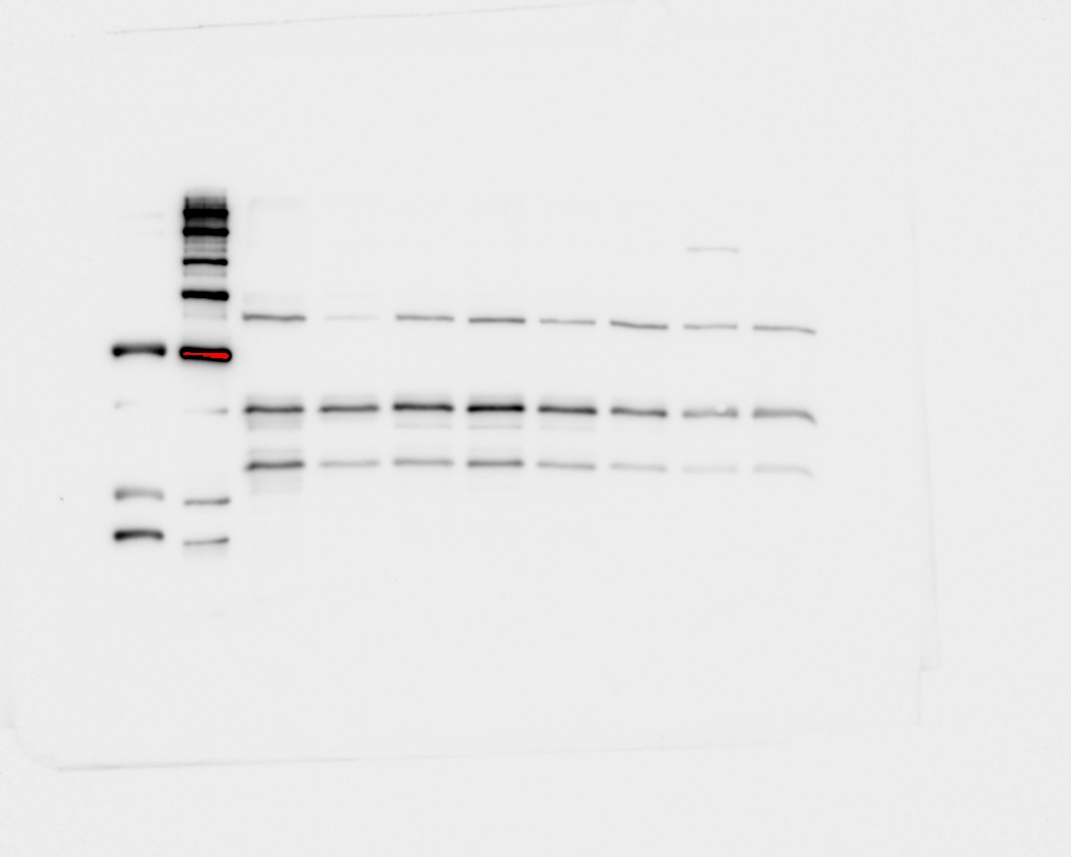

Supplement: Supplementary file 2 — Supplementary Western Blots [file 41418_2025_1623_MOESM2_ESM.docx]
